# Global Burden of Prostate Cancer and Association with Socioeconomic Status, 1990–2019: A Systematic Analysis from the Global Burden of Disease Study

**DOI:** 10.1007/s44197-023-00103-6

**Published:** 2023-05-06

**Authors:** Weiyu Zhang, Guiying Cao, Feng Wu, Yuliang Wang, Zheng Liu, Hao Hu, Kexin Xu

**Affiliations:** 1 Department of Urology, Peking University People’s Hospital, Beijing, 100044 China; 2 Department of Epidemiology and Biostatistics, School of Public Health, Peking University, Beijing, 100191 China; 3 Medical Informatics Center, Peking University, Beijing, 100191 China; 4 Institute for Disease Control and Prevention, Chinese PLA, Beijing, 100071 China; 5 School of Biomedical Sciences, Faculty of Medicine, The Chinese University of Hong Kong, Hong Kong, 999077 China; 6 Department of Science, Peking University People’s Hospital, Beijing, 100044 China

**Keywords:** Prostate cancer, Worldwide, Incidence, Mortality, Disability-adjusted life years, Trends

## Abstract

**Importance:**

Both the morbidity and mortality of prostate cancer are increasing worldwide. Updated evaluations of prostate cancer burden and its global, regional and national patterns are essential for formulating effective preventive strategies.

**Objective:**

To investigate prostate cancer incidence, mortality, and disability-adjusted life years (DALYs) between 1990 and 2019 to facilitate preventive measures and control planning.

**Methods:**

Annual incident cases, deaths, DALYs, age-standardized incidence rates (ASIRs), age-standardized mortality rates (ASMRs), and age-standardized DALYs rates (ASDRs) of prostate cancer between 1990 and 2019 were derived from the Global Burden of Diseases study 2019. Percentage changes in incident cases, deaths and DALYs and estimated annual percentage changes (EAPCs) in ASIRs, ASMRs and ASDRs were calculated to quantify temporal trends. Correlations between EAPCs and socio-demographic index (SDI) and universal health coverage index (UHCI) were evaluated by Pearson correlation analyses.

**Results:**

Globally, the number of incident cases, deaths, and DALYs of prostate cancer increased by 116.11%, 108.94%, and 98.25% from 1990 to 2019, respectively. The ASIR increased by an average of 0.26% (95% CI: 0.14%, 0.37%) per year between 1990 and 2019, while the ASMR and ASDR decreased by an average of – 0.75% (95% CI: – 0.84%, – 0.67%) and – 0.71% (95% CI: – 0.78%, – 0.63%) per year in this period, respectively. Epidemic trends in the burdens of prostate cancer were not uniform throughout different groups of SDI or geography. The burdens of prostate cancer varied across SDI regions, with an increasing trend in ASIR, ASMR, and ASDR in low and low-middle SDI regions between 1990 and 2019. A significant positive correlation between the EAPC in ASIR and UHCI was observed in countries with a UHCI < 70 (ρ = 0.37, p < 0.001).

**Interpretation:**

Prostate cancer remains a major global health burden due to the increase in incident cases, deaths, and DALYs in the past three decades. These increases are likely to continue as the population ages, which indicates a potential talent gap in the trained healthcare workforce. The diversity of prostate cancer development models implies the importance of specific local strategies tailored for each country’s risk factor profile. Prevention, early detection and more effective treatment strategies for prostate cancer are essential.

**Supplementary Information:**

The online version contains supplementary material available at 10.1007/s44197-023-00103-6.

## Introduction

Millions of men around world are affected by prostate cancer [1, 2]. The disease ranked second among common men cancers after lung cancer and accounted for 14.1% of newly diagnosed cancers in men globally [2]. In 112 countries, prostate cancer is the most frequently diagnosed cancer in men [2]. There were an estimated 1.41 million new cases and 375,304 prostate cancer deaths worldwide in 2020 [2]. Both high incidence and mortality rates make prostate cancer one of the leading threats of cancer-related death in men. The risk of prostate cancer increased strongly with age. Over 85% of newly diagnosed patients are aged > 60 years [1, 2]. In addition to age, a wide variety of modifiable behavioral, prostate-specific antigen (PSA) screening, metabolic, and environmental risk factors have been proposed to justify differences in the epidemiological burden of the disease [3, 4]. Furthermore, the global incidence and mortality rates of prostate cancer varied by ethnicity and geography. Prostate cancer was strikingly more common in developed countries (e.g., North America, Western and Northern Europe, and Australia) than in developing countries, since the former were characterized by a higher human development index (HDI) and gross domestic product, and consequently, higher life expectancy [5]. African descent men have highest prostate cancer mortality, including Afro-Caribbean, Sub-Saharan African, and African American [3, 5].

However, most current epidemiological studies on prostate cancer have focused on incidence, mortality and related trends [1–3, 5]. In addition to the cancer disease itself, the consequent complications, including metastasis, cardiovascular disease, lower urinary tract symptoms, cancer pain, and shortage of life expectancy, influence the physical and mental health of patients [3–5]. Social and family need to provide specific medical care, expenditures, time and energy to take care of prostate cancer patients. However, the burden of prostate cancer at the global, regional, and national levels is waiting to be updated. Thus, the current study used data provided by the Global Burden of Diseases (GBD) study, which is an ongoing global collaboration using all available epidemiological data to provide a comparative assessment of health loss from 328 diseases across 204 countries and territories [6]. The aims of this present study were to describe the most up-to-date trends in the rates of incidence, mortality, and disability-adjusted life years (DALYs) of prostate cancer at the global, regional and national levels as well as the associations with socioeconomic status at the national level from 1990 to 2019 based on the GBD study 2019. Knowing the burdens of prostate cancer as well as the temporal trends facilitates the initiation of more targeted prevention strategies, thereby promoting the precise prevention of prostate cancer.

## Methods

### Data source

We extracted annual data on incident cases, deaths, DALYs, age-standardized incidence rates (ASIRs), age-standardized mortality rates (ASMRs), and age-standardized DALYs rates (ASDRs) of prostate cancer from 1990 to 2019, by location, collected from the Global Health Data Exchange (GHDx) query tool (http://ghdx.healthdata.org/gbd-results-tool) [7]. Data were available from a total of 204 countries and territories, and these were categorized into 5 regions in terms of socio-demographic index (SDI) and 21 GBD regions according to geographical contiguity. In GBD study, the incident case and deaths of prostate cancer were identified according to the International Code of Diseases 10th, with codes C61-C61.9, D07.5, D29.1, D40.0 for mapping death, and C61-C61.9, Z12.5, Z80.42, Z85.46 for mapping new cases [8, 9]. Specific methods of GBD study 2019 estimation process for the incidence, mortality, and DALYs of prostate cancer were described elsewhere [6]. Briefly, the GBD study estimates the incidence of prostate cancer using DisMod-MR version 2.1, a meta-analysis tool that uses a compartmental model structure with a series of differential equations that synthesize sparse and heterogeneous epidemiological data for non-fatal diseases [10]. The Cause of Death Ensemble modelling model, a tool that selects models and covariates on the basis of out-of-sample performance, was applied to estimate mortality of prostate cancer in the GBD study [11]. DALYs of prostate cancer were calculated by summing years of life lost and years lived with disability [9].

Data of socioeconomic status, including SDI and universal health coverage index (UHCI) values in 2019 in 204 countries and territories were also collected from the GHDx query tool [7]. The SDI is a composite indicator of development status strongly correlated with health outcomes [7]. It is the geometric mean of 0 to 1 indices of lag distributed income per capita, average years of schooling for those ages 15 and older, and total fertility rate under the age of 25. A location with an SDI of 0 indicates a theoretical minimum level of development status relevant to health outcomes, while a location with an SDI of 1 indicates a theoretical maximum level [7]. The UHCI is developed following GBD 2019 and is comprised of 23 indicators drawn across a range of health service areas and is meant to represent healthcare needs over the life course [12]. The indicators of UHCI involved either direct measures of intervention coverage or outcome-based indicators to approximate access to quality care [13]. The UHCI indicators are reported on a scale of 0–100 [13]. The values of SDI and UHCI of 204 countries and terroirs in 2019 are shown in Table S1.

## Statistical analysis

To compare the incidence, mortality, and DALYs rates of prostate cancer across different populations, the ASIRs, ASMRs, and ASDRs were carried out by applying the age-specific rates for each location and year to a GBD World Standard Population to adjust for potential confounding of age structure [14]. The percentage change in incident cases of prostate cancer from 1990 to 2019 was calculated by the equation: Percentage change=$\frac{\mathrm{Incident\, cases\, in }\,2019 -\mathrm{ Incident\, cases\, in }\,1990}{\mathrm{Incident\, cases\, in\, }1990}\times$ 100%. The percentage changes in deaths and DALYs of prostate cancer were calculated using a similar equation. Estimated annual percentage change (EAPC) is a summary and widely used measure of age-standardized rates (ASRs) tend over a specified time interval. A regression line was fitted to the natural logarithm of the ASR, i.e., y = α + βx + ε, where y = ln (ASR) and x = calendar year. EAPC was calculated as $100\times {(e}^{\beta }-1)$ and its 95% confidence interval (CI) was calculated to reflect the temporal trend in ASR. The trend in ASRs was reflected in the EAPC value and its 95% CI: ASR is in an upward trend when the EAPC and the lower boundary of the 95% CI are positive; conversely, ASR is in a downward trend when EAPC and the upper boundary of the 95% CI are negative. We used this method to calculate the EAPCs in ASIR, ASMR, and ASDR of prostate cancer. Moreover, we used Pearson correlation analyses to evaluate the correlations between EAPCs and SDI values (2019) and UHCI values (2019) in 204 countries and territories, with polynomial curves modelled. All analyses were conducted with SAS 9.4 (SAS Institute, Inc., Cary, NC) and Origin 2019b. ArcGis 10.7 (ESRI, Redlands, CA, USA) was applied for the visualization of the geographical distribution of the burden of prostate cancer. A two-tailed *p* value less than 0.05 was considered statistically significant.

## Results

### Global trends in incidence, mortality, and DALYs rates of prostate cancer

Globally, the absolute number of incident cases, deaths, and DALYs of prostate cancer increased by 169.11% from 524.11 thousand in 1990 to 1.41 million in 2019, 108.94% from 233.00 thousand in 1990 to 486.84 thousand in 2019, and 98.25% from 4.36 million in 1990 to 8.64 million in 2019, respectively (Table 1). The overall ASIR of prostate cancer increased between 1990 and 2019 (EAPC = 0.26, 95% CI: 0.14, 0.37) from 34.13 per 100,000 in 1990 to 38.63 per 100,000 in 2019 (Table 2). The overall ASMR of prostate cancer decreased (EAPC = – 0.75, 95% CI: – 0.84, – 0.67) from 18.13 per 100,000 in 1990 to 15.28 per 100,000 in 2019 and the overall ASDR of prostate cancer decreased (EAPC = – 0.71; 95% CI: – 0.78, – 0.63) (from 186.30 per 100,000 in 1990 to 244.07 per 100,000 in 2019) in this period (Table 2).

**Table 1:** The incident cases, deaths, and DALYs of prostate cancer in 1990 and 2019 and their change trends from 1990 to 2019

Characteristics	Incident cases			Deaths			DALYs		
	1990 No. × 10^3^ (95% UI)	2019 No. × 10^3^ (95% UI)	Percentage change (%)	1990 No. × 10^3^ (95% UI)	2019 No. × 10^3^ (95% UI)	Percentage change (%)	1990 No. × 10^3^ (95% UI)	2019 No. × 10^3^ (95% UI)	Percentage change (%)
Overall	524.11 (409.13, 613.01)	1410.45 (1227.90, 1825.77)	169.11	233.00 (191.40, 268.88)	486.84 (420.50, 593.69)	108.94	4360.51 (3528.03, 4951.01)	8644.87 (7548.02, 10,559.87)	98.25
SDI									
Low	14.67 (10.95, 17.31)	35.45 (27.19, 41.73)	141.68	14.40 (10.57, 17.04)	35.22 (25.88, 41.60)	144.49	291.43 (213.45, 346.28)	681.26 (501.51, 806.49)	133.76
Low-middle	27.67 (23.40, 33.00)	100.39 (86.42, 117.90)	262.83	24.29 (19.95, 28.87)	66.63 (56.33, 78.82)	174.35	467.41 (384.21, 546.83)	1215.48 (1018.74, 1427.26)	160.04
Middle	53.50 (45.51, 61.37)	227.65 (195.37, 271.99)	325.49	39.01 (32.79, 44.65)	112.21 (94.00, 133.29)	187.68	743.44 (618.73, 838.43)	2026.39 (1710.33, 2399.59)	172.57
Middle-high	100.95 (87.17, 131.57)	317.96 (274.78, 403.99)	214.97	55.15 (48.23, 70.50)	114.28 (97.03, 142.02)	107.23	1047.29 (905.99, 1326.21)	2025.33 (1759.18, 2485.49)	93.39
High	326.93 (233.50, 380.99)	696.29 (588.17, 994.36)	112.98	99.94 (74.33, 116.05)	157.99 (131.25, 215.13)	58.09	1807.07 (1326.67, 2106.49)	2687.71 (2297.89, 3708.19)	48.73
GBD region									
High-income Asia Pacific	14.63 (12.46, 20.06)	67.98 (52.43, 91.27)	364.76	6.54 (5.61, 8.72)	19.38 (14.97, 23.66)	196.3	117.49 (101.23, 156.07)	298.74 (243.71, 380.29)	154.26
Central Asia	2.02 (1.62, 2.43)	4.77 (3.84, 5.73)	136.33	1.43 (1.19, 1.75)	2.57 (2.06, 3.06)	79.46	30.09 (24.32, 35.95)	53.79 (43.94, 64.79)	78.75
East Asia	28.09 (21.68, 33.59)	161.97 (126.34, 213.69)	476.66	21.20 (16.62, 25.55)	57.21 (45.38, 74.04)	169.86	420.16 (321.44, 505.20)	1053.23 (840.97, 1368.07)	150.67
South Asia	16.21 (12.38, 19.17)	53.95 (44.20, 69.73)	232.85	15.63 (11.72, 18.55)	42.22 (35.17, 54.87)	170.13	306.20 (230.99, 361.22)	763.89 (633.04, 986.04)	149.47
Southeast Asia	10.74 (8.58, 12.64)	44.47 (34.33, 52.60)	314.18	9.10 (7.31, 10.87)	27.21 (20.47, 32.07)	199	176.15 (141.73, 207.24)	510.04 (381.79, 602.06)	189.55
Australasia	8.75 (6.63, 10.65)	25.70 (19.14, 37.96)	193.79	2.70 (2.06, 3.28)	5.41 (4.43, 7.64)	100.37	50.54 (38.74, 61.89)	93.66 (77.67, 133.44)	85.33
Caribbean	7.17 (6.22, 9.10)	22.76 (17.70, 28.12)	217.37	4.02 (3.53, 5.10)	9.83 (7.85, 12.06)	144.49	69.88 (61.56, 87.70)	166.52 (133.66, 205.11)	138.3
Central Europe	14.86 (13.15, 20.13)	43.82 (32.06, 51.72)	194.79	9.56 (8.56, 13.07)	18.83 (14.10, 22.05)	96.98	178.29 (159.58, 242.30)	329.83 (243.66, 384.77)	84.99
Eastern Europe	21.99 (19.45, 32.51)	64.66 (45.77, 79.26)	194.06	10.52 (9.34, 16.07)	20.46 (15.20, 25.07)	94.39	228.15 (202.25, 336.36)	429.03 (310.36, 522.73)	88.05
Western Europe	141.08 (107.04, 171.38)	325.49 (267.13, 469.92)	130.71	62.77 (48.01, 75.26)	95.77 (79.23, 133.05)	52.58	1066.61 (804.34, 1282.69)	1499.83 (1261.99, 2106.39)	40.62
Andean Latin America	2.29 (1.95, 3.00)	12.09 (9.19, 15.59)	427.51	1.93 (1.65, 2.50)	6.26 (4.85, 7.86)	224.75	32.71 (28.06, 42.26)	101.03 (78.16, 129.24)	208.88
Central Latin America	11.83 (9.99, 15.69)	65.12 (51.32, 86.14)	450.62	6.54 (5.59, 8.99)	21.67 (16.87, 28.46)	231.58	117.54 (101.04, 157.57)	378.32 (297.27, 494.09)	221.87
Southern Latin America	6.71 (5.85, 9.41)	19.52 (14.60, 27.16)	191.11	4.98 (4.38, 7.06)	10.10 (8.38, 13.38)	102.9	88.66 (77.35, 123.51)	165.74 (139.82, 220.45)	86.94
Tropical Latin America	13.26 (11.82, 20.14)	56.94 (49.55, 84.10)	329.28	8.33 (7.33, 12.53)	23.90 (20.37, 34.73)	186.92	158.29 (142.31, 239.56)	423.13 (367.76, 615.83)	167.31
North Africa and Middle East	9.55 (7.68, 11.52)	47.47 (36.99, 55.85)	397	6.65 (5.38, 8.23)	19.09 (15.24, 22.50)	187.14	125.76 (102.03, 153.14)	345.76 (270.78, 404.98)	174.94
High-income North America	193.28 (132.82, 215.04)	331.89 (262.39, 494.58)	71.71	40.33 (28.44, 45.05)	54.85 (46.83, 79.75)	36.02	782.05 (543.04, 873.67)	1033.62 (897.37, 1515.66)	32.17
Oceania	0.21 (0.16, 0.29)	0.66 (0.48, 0.84)	205.12	0.18 (0.13, 0.24)	0.51 (0.38, 0.65)	180.99	3.73 (2.76, 5.02)	10.10 (7.47, 12.93)	170.68
Central Sub-Saharan Africa	1.75 (1.26, 2.28)	4.53 (3.18, 5.80)	158.69	1.75 (1.26, 2.27)	4.04 (2.81, 5.17)	130.47	35.80 (25.90, 46.41)	81.22 (57.40, 104.03)	126.84
Eastern Sub-Saharan Africa	6.06 (4.63, 7.24)	16.32 (12.86, 20.22)	169.56	5.93 (4.49, 7.07)	13.81 (10.91, 16.67)	132.61	122.57 (92.69, 146.32)	281.94 (220.71, 345.22)	130.01
Southern Sub-Saharan Africa	3.70 (2.89, 4.83)	10.11 (8.08, 11.91)	173.52	3.27 (2.58, 4.24)	7.41 (5.82, 8.41)	127.02	61.81 (48.45, 82.11)	143.41 (113.29, 165.24)	132.01
Western Sub-Saharan Africa	9.93 (6.13, 13.20)	30.23 (16.78, 40.61)	204.26	9.64 (5.88, 12.69)	26.30 (14.73, 35.42)	172.79	188.01 (113.97, 251.03)	482.04 (271.56, 654.18)	156.38

DALYs disability-adjusted life years, GBD global burden of disease, SDI socio-demographic index, UI uncertainty interval

**Table 2:** **The** ASIRs, ASMRs, and ASDRs of prostate cancer in 1990 and 2019 and their change trends from 1990 to 2019

Characteristics	ASIR per 100,000			ASMR per 100,000			ASDR per 100,000		
	1990 No. (95% UI)	2019 No. (95% UI)	EAPC No. (95% CI)	1990 No. (95% UI)	2019 No. (95% UI)	EAPC No. (95% CI)	1990 No. (95% UI)	2019 No. (95% UI)	EAPC No. (95% CI)
Overall	34.13 (26.83, 39.65)	38.63 (33.63, 49.83)	0.26 (0.14, 0.37)	18.13 (14.68, 21.19)	15.28 (13.00, 18.57)	– 0.75 (– 0.84, – 0.67)	286.30 (232.78, 326.21)	244.07 (211.78, 297.72)	– 0.71 (– 0.78, – 0.63)
SDI									
Low	16.25 (12.03, 19.15)	20.25 (15.11, 23.86)	0.87 (0.80, 0.94)	17.41 (12.75, 20.58)	19.47 (14.25, 22.91)	0.50 (0.42, 0.58)	290.94 (213.57, 344.65)	320.37 (235.13, 378.15)	0.41 (0.35, 0.47)
Low-middle	12.39 (10.42, 15.01)	18.53 (15.91, 21.75)	1.31 (1.17, 1.44)	12.28 (10.15, 14.86)	13.49 (11.41, 16.02)	0.23 (0.07, 0.38)	193.10 (159.11, 229.08)	212.95 (179.28, 250.81)	0.24 (0.13, 0.35)
Middle	14.48 (12.47, 16.91)	23.64 (20.30, 28.14)	1.67 (1.58, 1.75)	12.57 (10.77, 14.77)	12.81 (10.64, 15.21)	0.03 (– 0.07, 0.12)	191.17 (160.77, 220.20)	197.23 (165.56, 234.83)	0.05 (– 0.03, 0.14)
Middle-high	25.32 (21.89, 32.95)	35.74 (30.47, 45.13)	1.17 (0.96, 1.38)	16.42 (14.25, 21.16)	14.78 (12.45, 18.29)	– 0.51 (– 0.69, – 0.32)	263.48 (227.98, 337.21)	235.16 (202.66, 288.04)	– 0.53 (– 0.69, – 0.36)
High	75.24 (53.88, 87.91)	79.70 (67.26, 113.75)	– 0.07 (– 0.20, 0.07)	25.72 (19.16, 30.05)	17.65 (14.66, 23.94)	– 1.62 (– 1.72, – 1.52)	426.27 (313.79, 496.35)	303.49 (259.14, 417.27)	– 1.46 (– 1.55, – 1.38)
GBD region									
High-income Asia Pacific	19.80 (16.52, 26.86)	31.64 (24.54, 42.35)	2.13 (1.73, 2.53)	9.76 (8.18, 12.87)	8.60 (6.67, 10.53)	– 0.46 (– 0.55, – 0.36)	153.56 (131.87, 204.91)	138.04 (112.79, 177.07)	– 0.32 (– 0.43, – 0.20)
Central Asia	12.50 (10.17, 15.23)	18.62 (14.69, 22.13)	1.90 (1.70, 2.10)	9.78 (8.16, 12.22)	12.43 (9.75, 14.81)	1.24 (1.09, 1.39)	179.01 (146.39, 218.82)	209.81 (167.55, 250.27)	0.96 (0.80, 1.12)
East Asia	9.05 (7.34, 10.97)	17.72 (14.09, 23.07)	2.55 (2.45, 2.64)	8.21 (6.67, 10.24)	7.88 (6.36, 9.91)	– 0.16 (– 0.21, – 0.11)	126.37 (100.68, 151.44)	120.64 (96.98, 153.79)	– 0.19 (– 0.23, – 0.15)
South Asia	7.86 (5.94, 9.54)	9.26 (7.62, 11.98)	0.44 (0.36, 0.52)	8.45 (6.45, 10.32)	8.12 (6.67, 10.64)	– 0.28 (– 0.38, – 0.18)	132.99 (100.45, 158.71)	127.05 (105.59, 164.67)	– 0.28 (– 0.36, – 0.20)
Southeast Asia	11.66 (9.44, 13.87)	19.31 (14.80, 22.85)	1.71 (1.66, 1.75)	11.01 (8.94, 13.21)	13.56 (10.27, 15.97)	0.70 (0.65, 0.75)	179.32 (144.35, 214.50)	218.62 (164.66, 257.14)	0.65 (0.60, 0.69)
Australasia	85.99 (65.21, 104.97)	108.35 (80.76, 159.34)	0.30 (– 0.05, 0.64)	30.18 (23.06, 36.71)	22.32 (18.28, 31.42)	– 1.50 (– 1.65, – 1.35)	504.89 (386.32, 617.67)	390.42 (323.54, 552.32)	– 1.32 (– 1.49, – 1.16)
Caribbean	60.49 (52.62, 76.99)	95.83 (74.43, 118.13)	1.55 (1.37, 1.72)	36.55 (31.95, 46.57)	42.75 (34.14, 52.35)	0.49 (0.30, 0.67)	588.18 (518.30, 739.15)	704.84 (565.44, 866.14)	0.57 (0.42, 0.73)
Central Europe	25.62 (22.95, 34.73)	46.76 (34.28, 55.20)	2.31 (2.10, 2.52)	18.27 (16.29, 25.11)	21.56 (16.05, 25.11)	0.60 (0.44, 0.77)	304.61 (273.64, 413.55)	357.74 (264.07, 417.95)	0.60 (0.44, 0.75)
Eastern Europe	24.07 (21.23, 36.63)	48.80 (34.95, 59.51)	2.94 (2.74, 3.14)	13.75 (12.16, 21.54)	17.53 (12.95, 21.71)	0.90 (0.83, 0.98)	254.24 (224.80, 386.72)	336.41 (244.51, 407.66)	1.03 (0.94, 1.12)
Western Europe	59.45 (45.12, 72.27)	78.67 (64.34, 113.34)	0.73 (0.41, 1.06)	28.99 (22.04, 34.78)	21.60 (17.88, 29.78)	– 1.35 (– 1.49, – 1.22)	454.88 (343.09, 545.66)	349.18 (293.71, 489.54)	– 1.22 (– 1.37, – 1.08)
Andean Latin America	26.83 (22.78, 35.21)	47.52 (36.13, 61.32)	2.21 (2.05, 2.37)	23.67 (20.22, 30.89)	25.50 (19.68, 32.03)	0.45 (0.32, 0.58)	372.20 (318.37, 481.34)	397.17 (307.58, 507.19)	0.39 (0.28, 0.50)
Central Latin America	33.23 (28.20, 44.47)	62.29 (49.43, 82.24)	1.81 (1.48, 2.15)	20.43 (17.42, 28.26)	22.11 (17.32, 29.24)	– 0.06 (– 0.27, 0.14)	332.79 (286.32, 450.35)	369.29 (289.00, 482.77)	0.03 (– 0.16, 0.23)
Southern Latin America	35.74 (31.11, 50.09)	53.97 (40.42, 74.92)	1.23 (0.92, 1.55)	28.87 (25.17, 41.26)	29.53 (24.42, 38.78)	– 0.12 (– 0.37, 0.14)	463.56 (408.64, 652.45)	462.58 (387.30, 614.38)	– 0.21 (– 0.46, 0.04)
Tropical Latin America	36.65 (32.31, 55.18)	54.94 (47.67, 81.28)	1.25 (0.91, 1.59)	26.64 (23.00, 40.04)	25.48 (21.50, 37.14)	– 0.24 (– 0.48, 0.00)	431.89 (382.77, 650.59)	417.24 (360.53, 605.72)	– 0.26 (– 0.52, 0.00)
North Africa and Middle East	13.36 (10.76, 16.42)	23.71 (18.52, 27.88)	2.06 (1.93, 2.20)	11.05 (8.95, 13.86)	11.71 (9.39, 13.95)	0.24 (0.08, 0.40)	176.19 (142.47, 217.67)	186.81 (147.72, 219.52)	0.22 (0.11, 0.33)
High-income North America	127.03 (88.19, 141.59)	113.02 (89.54, 168.18)	– 0.89 (– 1.04, – 0.73)	28.68 (20.47, 31.96)	19.08 (16.25, 27.72)	– 1.83 (– 1.99, – 1.67)	524.78 (366.74, 586.04)	355.25 (308.31, 519.22)	– 1.77 (– 1.92, – 1.61)
Oceania	20.82 (15.89, 27.76)	26.48 (19.89, 33.17)	0.90 (0.86, 0.94)	20.55 (15.44, 27.64)	24.18 (18.15, 30.53)	0.68 (0.63, 0.74)	323.04 (240.50, 436.00)	375.63 (279.62, 475.23)	0.64 (0.59, 0.69)
Central Sub-Saharan Africa	24.39 (17.16, 32.17)	29.16 (20.09, 37.48)	0.56 (0.50, 0.62)	27.17 (18.82, 35.80)	29.92 (20.54, 38.38)	0.28 (0.25, 0.31)	434.47 (306.86, 565.54)	469.77 (325.09, 602.73)	0.21 (0.16, 0.25)
Eastern Sub-Saharan Africa	20.80 (15.78, 24.49)	26.56 (20.98, 32.42)	0.87 (0.83, 0.91)	22.01 (16.54, 25.79)	24.80 (19.56, 29.64)	0.41 (0.39, 0.44)	381.44 (288.81, 450.51)	432.87 (342.49, 523.92)	0.44 (0.42, 0.47)
Southern Sub-Saharan Africa	38.72 (30.36, 49.67)	53.16 (41.87, 60.83)	1.11 (0.96, 1.27)	37.58 (29.77, 47.83)	45.08 (34.35, 50.53)	0.62 (0.38, 0.86)	607.11 (478.76, 792.88)	726.31 (574.70, 822.50)	0.63 (0.37, 0.90)
Western Sub-Saharan Africa	29.66 (18.77, 38.84)	43.52 (24.40, 57.94)	1.58 (1.46, 1.70)	31.39 (19.42, 40.49)	41.74 (23.48, 55.68)	1.22 (1.12, 1.32)	510.71 (312.45, 669.18)	661.04 (370.00, 891.73)	1.11 (1.02, 1.20)

ASDR age-standardized DALYs rate, *ASIR* age-standardized incidence rate, *ASMR* age-standardized mortality rate, *CI* confidence interval, *EAPC* estimated annual percentage change, *GBD* global burden of disease, *SDI* socio-demographic index; *UI* uncertainty interval

### Regional trends in incidence, mortality, and DALYS rates of prostate cancer

For 5 SDI regions, the number of incident cases, deaths, and DALYs of prostate cancer increased in all regions, with the largest increase in middle SDI region (incident cases: 325.49%; deaths: 187.68%; DALYs: 172.57%) from 1990 to 2019 (Table 1). The ASIR of prostate cancer increased in low, low-middle, middle, and middle-high SDI regions, with the largest increase in middle SDI regions (EAPC = 1.67; 95% CI: 1.58, 1.75), while the ASIR remained stable in high SDI region in this period (Table 2). The ASMR of prostate cancer increased in low (EAPC = 0.50; 95% CI: 0.42, 0.58) and low-middle (EAPC = 0.23, 9% CI: 0.07, 0.38) SDI regions but decreased in middle-high (EAPC = -0.51, 95% CI: -0.69, -0.32) and high SDI regions (EAPC = – 1.61, 95% CI: – 1.72, – 1.52) between 1990 and 2019 (Table 2). Similarly, an increasing trend of the ASDR of prostate cancer was observed in low (EAPC = 0.41; 95% CI:0.35, 0.47) and low-middle (EAPC = 0.24, 9% CI: 0.13, 0.35) SDI regions but a decreasing trend was observed in middle-high (EAPC = – 0.53, 95% CI: – 0.69, – 0.36) and high SDI regions (EAPC = – 1.46, 95% CI: – 1.55, – 1.38) in this period (Table 2). The ASMR and ASDR of prostate cancer remained stable in middle SDI region (Table 2).

For the 21 GBD regions, the number of incident cases, deaths, and DALYs of prostate cancer increased in all regions, with the largest increase of incident cases in East Asia (476.66%) and the largest increase of deaths (231.58%) and DALYs (221.87%) in Central Latin America (Table 1). The ASIR of prostate cancer increased in nearly all regions (19 GBD regions), with the largest increase in Eastern Europe (EAPC = 2.94, 95% CI: 2.74, 3.14), while the ASIR remained stable in Australasia and decreased in High-income North America (EAPC = – 0.89, 95% CI: – 1.04, – 0.73) (Table 2). The ASMR of prostate cancer increased in more than half of regions (12 GBD regions) with the largest increase in Central Asia (EAPC = 1.24; 95% CI: 1.09, 1.39), but decreased in 7 GBD regions with the largest decrease in High-income North America (EAPC = -1.83; 95% CI: – 1.99, – 1.67) (Table 2). The ASMR remained stable in the rest 2 GBD regions. Similar to the ASMR of prostate cancer, we observed an increasing trend in ASDR in 12 GBD regions with the largest increase in Western Sub-Saharan Africa (EAPC = 1.11; 95% CI: 1.02, 1.20) but a decreasing trend in 6 GBD regions with the largest decrease in High-income North America (EAPC = – 1.77; 95% CI: – 1.92, 1.61) (Table 2).

### National trends in incidence, mortality, and DALYS rates of prostate cancer

**Fig. 1:**
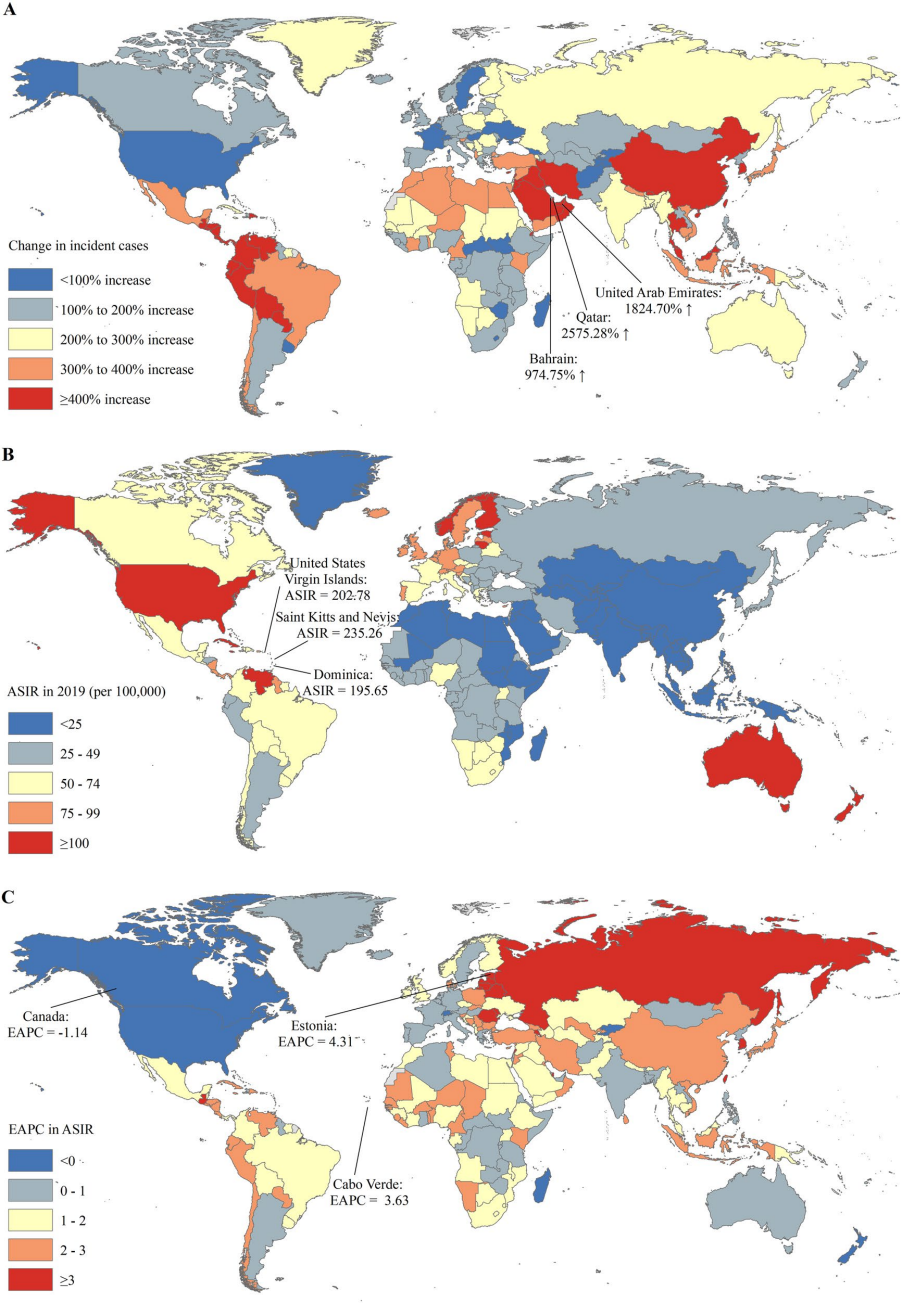
The global trends in the incidence of prostate cancer in 204 countries and territories. **A** The relative change in incident cases of prostate cancer between 1990 and 2019; **B** The ASIRs of prostate cancer in 2019; **C** The EAPCs of ASIRs of prostate cancer from 1990 to 2019. Note: *ASIR* age-standardized incidence rate; *EAPC* estimated annual percentage change

For 204 countries or territories, the absolute number of incident cases of prostate cancer in the United States (0.31 million) and China (0.15 million) accounted for approximately one third of global incident cases (1.41 million) in 2019 (Table S2). The countries with the most pronounced increase of incident cases of prostate cancer were Qatar (2575.28%) and United Arab Emirates (1824.70%) (Table S2 and Fig. 1A). The ASIR varies considerably across the world, with the largest ASIR in Saint Kitts and Nevis (235.26 per 100,000), followed by United States Virgin Islands (208.78 per 100,000) and Dominica (195.65 per 100,000) in 2019 (Table S2 and Fig. 1B). The ASIRs of prostate cancer were deemed to be in an increasing trend in 188 countries or territories, with the largest increase in Estonia (EAPC = 4.31; 95% CI: 3.92, 4.71), followed by Carbo Verde (EAPC = 3.63; 95% CI: 2.86, 4.42) (Table S2 and Fig. 1C). The ASIRs remained stable in 9 countries or territories, such as Samoa, Luxembourg, and Madagascar (Table S2). The ASIRs were deemed to be in a decreasing trend in 7 countries or territories, with the largest decrease in Canada (EAPC = – 1.44; 95% CI: – 1.25, – 0.77) (Table S2 and Fig. 1C).

**Fig. 2:**
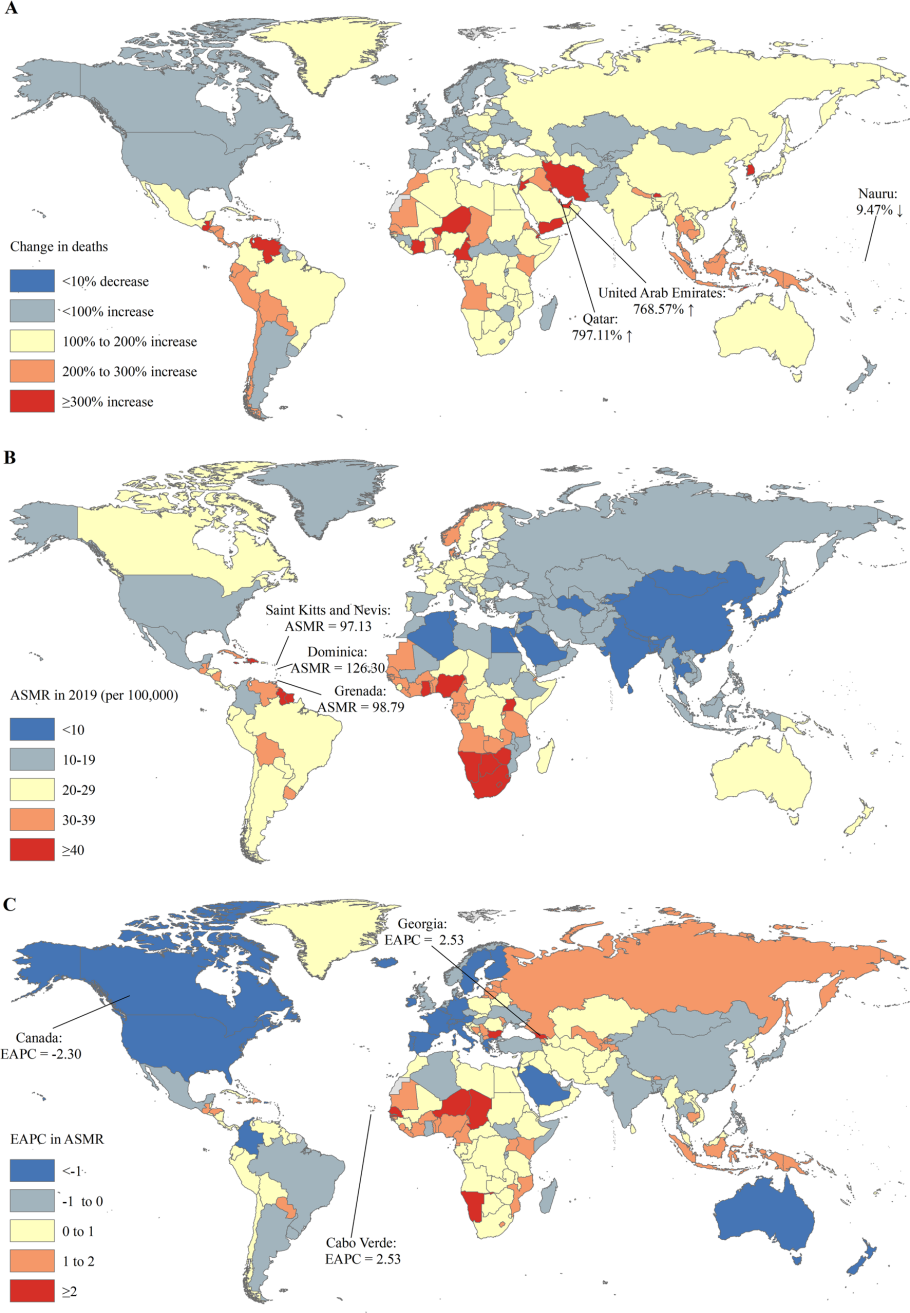
The global trends in the mortality of prostate cancer in 204 countries and territories. **A** The relative change in mortality of prostate cancer between 1990 and 2019; **B** The ASMRs of prostate cancer in 2019; **C** The EAPCs of ASIRs of prostate cancer from 1990 to 2019. Note: *ASMR* age-standardized mortality rate; *EAPC* estimated annual percentage change

The largest number of prostate cancer deaths was observed in China (54.39 thousand), followed by the United States (48.32 thousand) and India (32.11 thousand) in 2019 (Table S3). The most pronounced increase in incident cases of prostate cancer was observed in Qatar (797.11%) and the United Arab Emirates (768.57%) (Table S3 and Fig. 2A). The ASMR of prostate cancer varied significantly across the world, with the largest ASMR observed in Dominica (126.30 per 100,000), Grenada (98.79 per 100,000), and Saint Kitts and Nevis (97.13 per 100,000) (Table S4 and Fig. 2B). The ASMRs were deemed to be increasing in 119 countries or territories, with the largest increase in Georgia (EAPC = 2.53; 95% CI: 1.92, 3.14), followed by Cape Verde (EAPC = 2.53; 95% CI: 1.71, 3.37). The ASMRs were deemed to be in a decreasing trend in 57 countries or territories, with the largest decrease in Canada (EAPC = – 2.30; 95% CI: – 2.53, − 2.07) (Table S3 and Fig. 2C). The ASMRs remained stable in 28 countries or territories, such as Argentina, Poland, and the United Arab Emirates (Table S3).

**Fig. 3:**
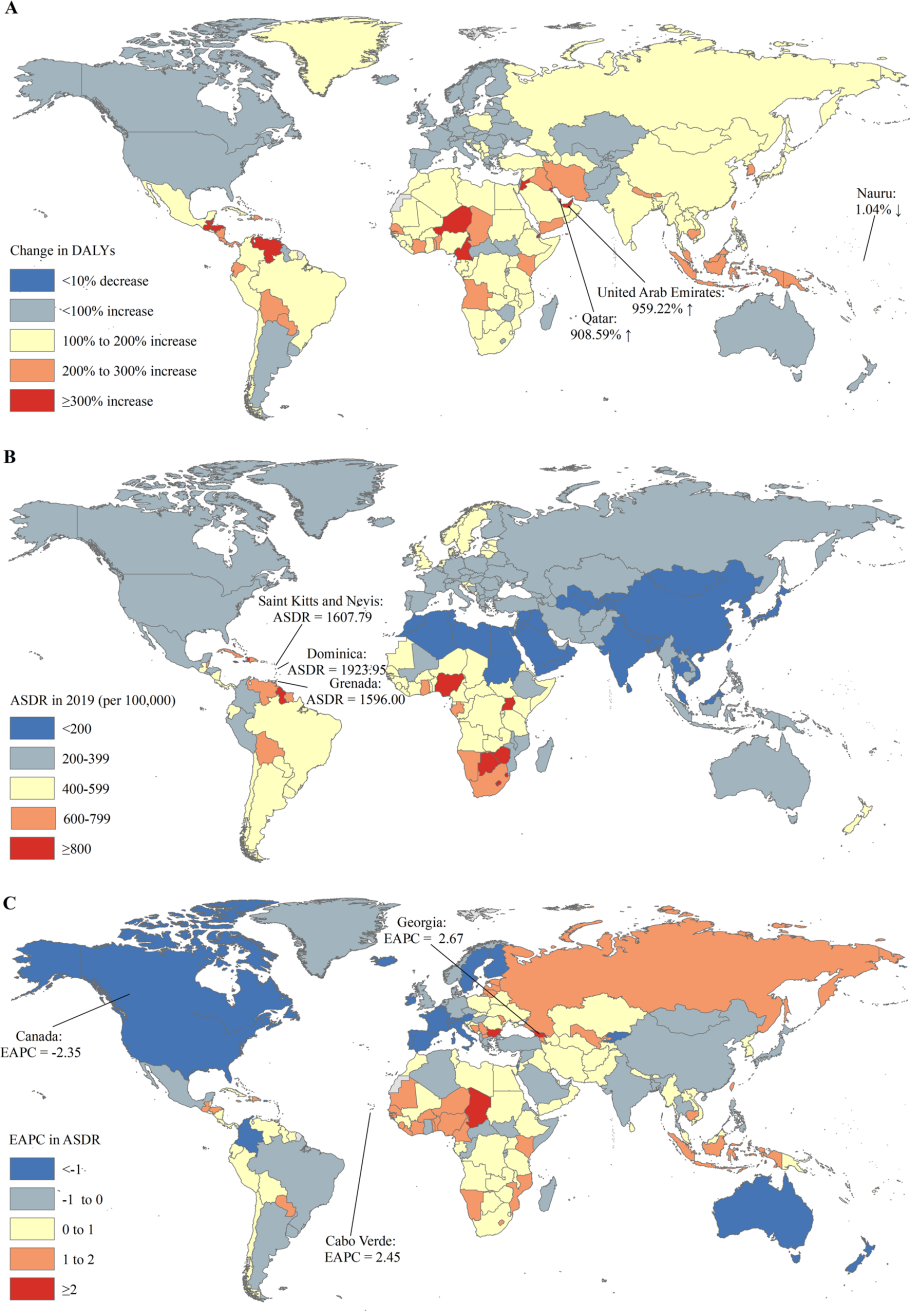
The global trends in the DALYs of prostate cancer in 204 countries and territories. **A** The relative change in DALYs of prostate cancer between 1990 and 2019; **B** The ASDRs of prostate cancer in 2019; **C** The EAPCs of ASDRs of prostate cancer from 1990 to 2019. Note: *ASR* age-standardized rate; DALYs, disability-adjusted life years; EAPC estimated annual percentage change

Across all countries or territories, China had the largest number of DALYs of prostate cancer (1.00 million), followed by the United States (0.93 million) in 2019 (Table S4). The United Arab Emirates (959.22%) and Qatar (908.59%) had the most pronounced increase of DALYs of prostate cancer (Table S4 and Fig. 3A). The ASDR varies considerably across the world, with the largest ASDR in Dominica (1923.95 per 100,000), Saint Kitts and Nevis (1607.79 per 100,000), and Grenada (1596.00 per 100,000) (Table S4 and Fig. 3B). The ASDRs of prostate cancer were deemed to be in an increasing trend in 119 countries or territories, with the largest increase in Georgia (EAPC = 2.67; 95% CI: 2.04, 3.30) and Cape Verde (EAPC = 2.45; 95% CI: 1.61, 3.30). The ASDRs were deemed to be in a decreasing trend in 61 countries or territories, with the largest decrease in Canada (EAPC = – 2.35; 95% CI: -2.63, – 2.07) (Table S4 and Fig. 3C), while the ASDRs remained stable in 24 countries or territories (Table S3).

### Association between EAPC and socioeconomic status

**Fig. 4:**
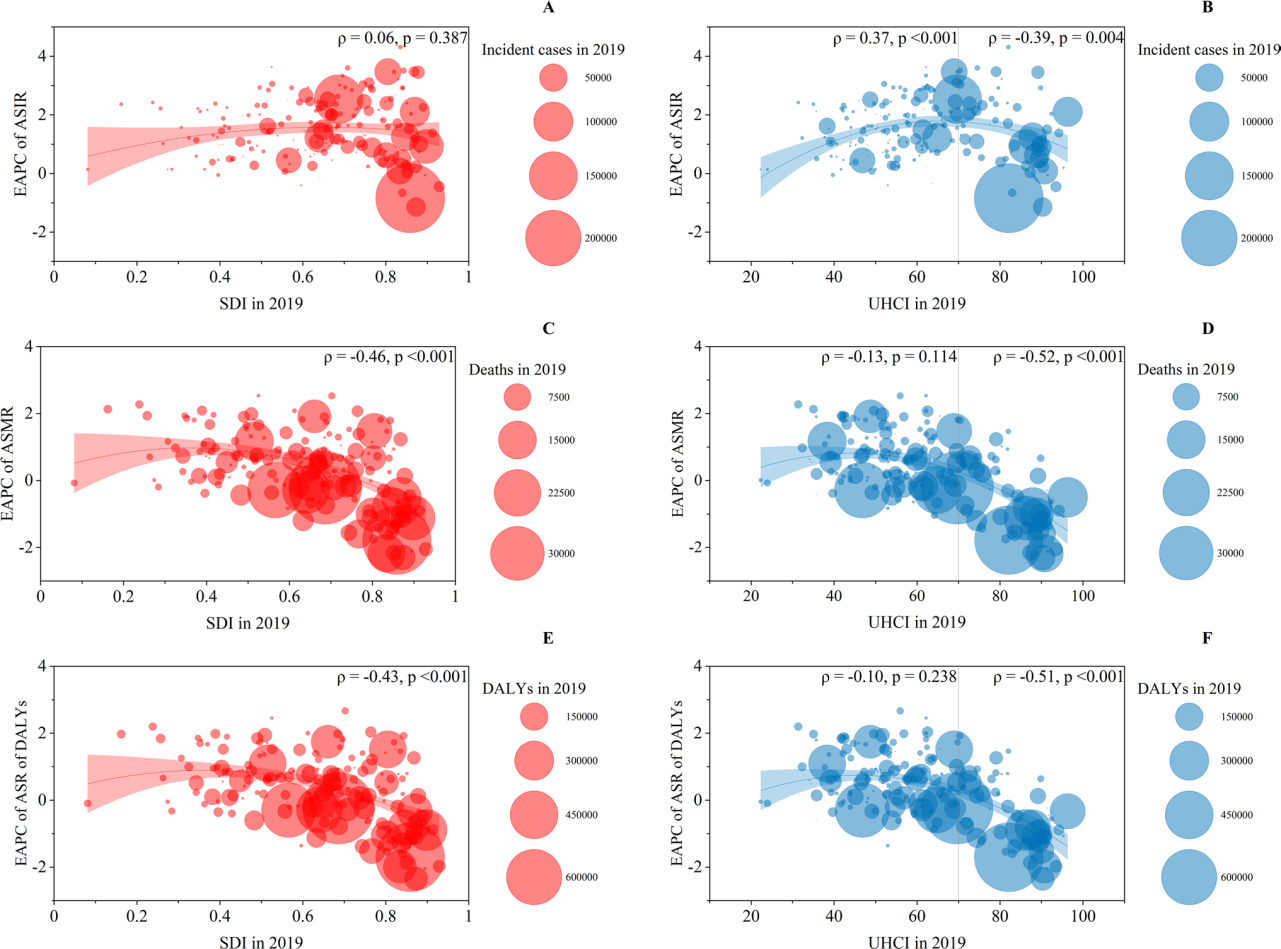
The EAPCs of ASIRs, ASMRs, and ASDRs of prostate cancer at the country and territorial levels. **A**, **C**, **E** The correlation between EAPC and SDI in 2019. **B**, **D**, **F** The correlation between EAPC and UHCI in 2019. The numbers of incident cases, deaths, and DALYS from 204 countries and territories in 2019 are represented by circles. The size of the circles increased with the incident cases, deaths, and DALYs. The ρ indices and p values were derived from Pearson correlation analysis

A significant negative correlation was detected between SDI in 2019 and EAPC in ASMRs (ρ = – 0.46, p < 0.001) and ASDRs (ρ = – 0.43, p < 0.001) of prostate cancer (Fig. 4C, E), while a nonsignificant correlation was observed between SDI in 2019 and EAPC in ASIRs of prostate cancer (ρ = 0.06, p = 0.387) (Fig. 4A).

For the correlation of EAPC in ASIRs of prostate cancer and UHCI in 2019, we found a significant positive correlation for ASIRs (ρ = 0.37, p < 0.001) in the countries and territories with a UHCI < 70, while a significant negative correlation was observed in the countries and territories with a UHCI ≥ 70 (ρ = – 0.39, p = 0.004) (Fig. 4B). A significant negative correlation was detected between the UHCI in 2019 and EAPCs in ASMRs and ASDRs of prostate cancer in countries or territories with a UHC index < 70 or UHCI ≥ 70 (Fig. 4D, F).

## Discussion

Prostate cancer was presented as the second most common cancer among the male population globally, accounting for 14.1% of all incident cancer cases and 6.8% of all deaths in men in 2020 [2]. With regard to geographical location, incidence and mortality rates differed. Our findings reported a global increase in the absolute number of incident cases, deaths and DALYs of prostate cancer by 169.11%, 108.94% and 98.25% from 1990 to 2019, respectively. The ASIR increased (EAPC = 0.26, 95% CI: 0.14, 0.37), while the ASMR decreased (EAPC = – 0.75, 95% CI: – 0.84, – 0.67) and the ASDR (EAPC = – 0.71, 95% CI: – 0.78, – 0.63) of prostate cancer decreased over the same period. According to the most recent GLOBOCAN report in 2020, the ASIR of prostate cancer was 31 per 100 000 (life-time cumulative risk: 3.9%) [15]. Northern Europe had the highest ASIR at all ages (83 per 100 000), followed by Western Europe (78), Caribbean (76), and Australia and New-Zealand (76), while the lowest ASIR at all ages was reported in South-Central Asia (6.3 per 100 000), South-Eastern Asia (14), and Northern Africa (17) [15]. The overall ASMR for 2020 was 7.7 per 100 000, with the highest ASMR in the Caribbean and the lowest in South-Central Asia [15]. Our findings were generally consistent with the GLOBOCAN report. Partly due to the different data sources and estimation models, our estimates presented higher results than the GLOBOCAN report. Continuing large population with prostate cancer, consistently high expenditure on prostate cancer and related health issues, and heavy disease burden of prostate cancer were exposed in the present study. Manifold diversities on disease burden of prostate cancer were shown in demography, socioeconomics, geography.

The overall global increased percentage change in incident cases was shown in the present study, which was mainly attributable to population growth. An increase in ASIR with a concomitant increase in absolute numbers was observed in most regions and nations. The increase in absolute numbers of prostate cancer juxtaposed with a significant increase in the ASIR indicated that changes in population age structure and population growth had little influence on this increase. Based on the present findings, a high ASIR in 2019 was accompanied by a low EAPC of ASIR in GBD regions of High-income North America, Australasia, Western Europe and the Caribbean, indicating that the increasing absolute incident number of prostate cancer cases depended on population growth. Differing from the stabilizing incidence trends in developed regions, heterogeneous trends were shown among other regions. Sophisticated screening and diagnosis strategies played roles in addition to the well-established risk factors for prostate cancer, such as family history, germline mutations, race, and individual, environmental and occupational risk factors. PSA has been widely adopted for the detection of prostate cancer and has been recommended by almost all authorized clinical guidelines for more than 40 years [16]. The global increased detection led to the followed increased incidence of prostate cancer. The EAPC of ASIR was highest in middle SDI regions, indicating a new and gradual promotion of screening programs there. A low EAPC of ASIR was found in low SDI regions accompanied by the highest EAPC of ASMR and EAPC of ASDR. This was potentially due to an inefficient diffusion of early detection policies, and the prostate cancer patients had been diagnosed at a late stage. The lowest EAPC of ASIR was present in high SDI regions, where early detection was conducted two or three decades ago. Men at the late stage of prostate cancer were predicted to have a poor overall survival of only 30% at 5 years [17].

Life expectancy for individuals with localized prostate cancer at an early stage could be as high as 99% over 10 years [18]. The Cluster Randomized Trial of PSA Testing for Prostate Cancer (CAP) [19], reporting data at 10 years, and the randomized Prostate, Lung, Colorectal and Ovarian screening trial (PLCO) [20], reporting data at 16 years, did show an increase in the detection of low-risk prostate cancer. Early detection for localized or indolent prostate cancer, via PSA testing plus digital rectal examination (DRE) and multiparameter magnetic resonance imaging (mp-MRI), played a pivotal role in increasing the incidence and potentially reducing mortality, and consequently extending the life expectancy of men with prostate cancer. This hypothesis aligned with our findings. However, overdiagnosis and overtreatment for clinically indolent prostate cancer exist [21]. No survival benefit in favor of PSA-based screening was concluded, according to the results from CAP in the UK [19], PLCO in the US [20], and the Prostate Cancer Intervention VS Observation Trial (PIVOT) [22] in the US. The European Randomized Trial of Screening for Prostate Cancer (ERSPC) in Europe [23] was the only screening study to report a benefit in favor of screening, but at a high cost of overdiagnosis and subsequent overtreatment. The ERSPC study showed a 20% reduction in prostate cancer-specific mortality, but 570 men needed to be screened by serum PSA testing for the prevention of one prostate cancer-related death [23]. Based on evidence from global randomized clinical trials, the US Preventive Services Task Force (USPSTF) made recommendations against population- and PSA-based screening for prostate cancer [16]. Clinical guidelines released by public health organizations in developed countries and regions, such as the American Urological Association, Canadian Task Force on Prevention Health Care, Japanese Urological Association, and European Association of Urology, provided more accurate prostate cancer screening recommendations suggesting that men at an average risk, over certain years of age, with long life expectancy decide about PSA testing [16]. Some kinds of risk calculators applied for access to PSA-based prostate cancer screening could be foreseen. In addition, the decline in the ASIR of prostate cancer in the study period was also supposed to be a so-called “backlog” after initial detection of prevalent prostate cancer that had accumulated as a result of incidence in previous years after initiation of PSA screening. Application of mp-MRI in prostate cancer screening and evaluation developed in recent years. Callender T. et al. introduced an age-based and risk-stratified MRI-first strategy that would not only result in fewer prostate cancer deaths, biopsies, and overdiagnoses, but also be cost-effective [24]. Florian A Distler et al. suggested the combination of PSA density and PI-RADS for the accuracy of prostate cancer prediction and avoidance of prostate biopsies [25].

Varied patterns of EAPC of ASIR, ASMR and ASDR have been observed around the world. It was found that the trend of ASIR did not necessarily parallel to the ASMR and ASDR. The high SDI regions showed a decreasing trend in the ASIR, ASMR and ASDR, while the low SDI regions showed a slowly increasing trend in ASIR but the highest increase in the ASMR and ASDR. The middle SDI regions presented the highest increasing speed in ASIR but remained intermediate in ASMR and ASDR. In High-income Asia Pacific and East Asia, the ASIR increased, while the ASMR and ASDR decreased. The GBD regions of High-income North America, Australasia, South Asia, and Western Europe represented a stable or decreased ASIR, along with decreased ASMR and ASDR. What stood out was the slow-growing ASIR and relatively high ASMR and ASDR in Africa, which consisted the main of low SDI regions. The rapidly developing economies during the late twentieth and early twenty-first century provided equitable access to affordable, good-quality care with financial sustainability [26]. The economic base determined the superstructure. In pace with economic growth, treatments for prostate cancer, including deferred treatment, radical prostatectomy, radiotherapy, hormonal therapy, investigational therapies, and enhancement of access to medical care, have been promoted.

Some traditional views about racial disparities were challenged by the present results, especially for Africa and Asia. It was widely accepted that men of African and Caribbean descent were more likely to be diagnosed with prostate cancer and present with distant metastases [27]. Men of Asian descent were less susceptible to prostate cancer [27]. In our findings, the mortality-to-incidence ratio (MIR) in GBD regions of Africa, Caribbean and Asia was broadly in line with the literatures. This was the so-called racial disparities. However, the viewpoint has been challenged. Resulting from the Surveillance, Epidemiology and End Results (SEER) databases, Rober T Dess et al. concluded that the black race didn’t appear to be associated with inferior stage-for-stage prostate cancer specific mortality [28]. After full adjustment, black men with prostate cancer turned out to be socioeconomic barriers to timely and quality care, and inadequateness of standardized and evidence-based treatment. Rober T Dess et al. also drew attention to the increased hazard of other-cause mortality among black men with prostate cancer, commonly cardiovascular and cerebrovascular disease [28]. Asian men are traditionally considered to have a low-incidence of prostate cancer. With a wide geographical span, significant variations existed within Asia. Based on our findings, geographic South Asia presented a much higher MIR, while geographic East Asia present a much lower MIR, indicating distinct differences among Asian regions in survival and early diagnosis of prostate cancer. Several groups have raised the hypothesis that genomic differences made vary epidemiology in Asia. Genetic mutations were thought to be a primary driver of prostate cancer. The accumulation of genetic mutations in a certain population explained the varied regional ASIR and ASMR. In geographic East Asia, including China, Japan and Republic of Korea, the rates of Erythroblast transformation-specific-related gene (ERG) oncoprotein in-positive prostate cancer were low (13–22%) [29]. Occupying the largest population in geographic East Asia, the Chinese population showed a low mutation burden, with *FOXA1* more frequently (41%) mutated, increased copy number alterations and chromosomal rearrangements [29]. Global discrepancies in lifestyle and diet explained the varied ASIRs and ASMRs among regions and nations. The Westernized diet and sedentary lifestyle were in accordance with a doubling of prostate cancer incidence (43.3% and 20.8%) of latent prostate cancer in Japanese residents from 1983 to 2013 [30].

The GBD study provides a better understanding of the trends in the incidence, mortality, and DALY rates of prostate cancer over the last couple decades globally. Some limitations in this study should also be acknowledged. First, the accuracy and robustness of GBD estimates largely depend on the quality and quantity of data used in the modeling [10]. Second, due to the GBD study taking the country as its basic unit, the incidence, mortality, and DALYs of prostate cancer might be a margin of bias in countries in lack of national systematic surveillance and population-based studies of prostate cancer. Third, although the standardization makes the incidence, mortality, and DALYs rates of prostate cancer comparable at the global, regional and national levels, the ASIR, ASMR, and ASDR of prostate cancer only reflect the burden of prostate cancer for one region or country under the age structure of a GBD World Standard Population.

## Conclusions

As the population aged, prostate cancer incident cases and deaths continue to rise year by year. The disease burden of prostate cancer is increasing. Benefitting from fast improvement in diagnostics and intervention strategies, the overall ASMR and ASDR of prostate cancer decreased at the global level. The present study found large heterogeneities in incidence and mortality within regions and nations, indicating discrepancies in racial differences, genetic predisposition, socioeconomic factors and so on. Further study is required to expand knowledge on the tumorigenesis, progression, invasion and metastasis of prostate cancer and to improve the prognosis, especially in developing countries. Absolutely, prostate cancer remains a noteworthy public health challenge across the globe. The results from the present study may be a reference for policy makers to develop effective prevention and treatment strategies, further achieve global targets and improve equity in prostate cancer care.

## Supplementary Information

Below is the link to the electronic supplementary material.

Supplementary file1 (DOCX 184 KB)

## Data Availability

The datasets analyzed during the current study are publicly available.

## Abbreviations

CI: Confidence interval
EAPCs: Estimated annual percentage changes
GBD: Global burden of diseases
DALYs: Disability-adjusted life years
ASDRs: Age-standardized DALYs rates
ASIRs: Age-standardized incidence rates
ASMRs: Age-standardized mortality rates
ASRs: Age-standardized rates
SDI: Socio-demographic index
UHCI: Universal health coverage index
PSA: Prostate-specific antigen
