# Supplementary material for: Global Burden of Prostate Cancer and Association with Socioeconomic Status, 1990–2019: A Systematic Analysis from the Global Burden of Disease Study
Source: J Epidemiol Glob Health. 2023 May 6;13(3):407–21. doi: 10.1007/s44197-023-00103-6 (PMC10469111; doi:10.1007/s44197-023-00103-6)
Supplement: Supplementary file 1 — Supplementary file1 (DOCX 184 KB) [file 44197_2023_103_MOESM1_ESM.docx]

**Table S1. The values of SDI and UHCI of 204 countries and territories in 2019.**

| Region | SDI | UHCI |
| --- | --- | --- |
| Afghanistan | 0.343 | 39.295 |
| Albania | 0.681 | 69.626 |
| Algeria | 0.652 | 64.855 |
| American Samoa | 0.712 | 53.197 |
| Andorra | 0.894 | 91.748 |
| Angola | 0.470 | 39.158 |
| Antigua and Barbuda | 0.743 | 59.630 |
| Argentina | 0.708 | 61.158 |
| Armenia | 0.689 | 62.435 |
| Australia | 0.839 | 89.423 |
| Austria | 0.849 | 86.370 |
| Azerbaijan | 0.683 | 48.181 |
| Bahamas | 0.796 | 60.565 |
| Bahrain | 0.751 | 70.577 |
| Bangladesh | 0.483 | 53.883 |
| Barbados | 0.742 | 61.207 |
| Belarus | 0.745 | 70.462 |
| Belgium | 0.851 | 87.301 |
| Belize | 0.603 | 54.280 |
| Benin | 0.352 | 44.624 |
| Bermuda | 0.813 | 77.549 |
| Bhutan | 0.455 | 51.301 |
| Bolivia | 0.566 | 52.399 |
| Bosnia and Herzegovina | 0.718 | 64.184 |
| Botswana | 0.634 | 57.518 |
| Brazil | 0.640 | 64.828 |
| Brunei | 0.823 | 65.532 |
| Bulgaria | 0.764 | 62.557 |
| Burkina Faso | 0.257 | 41.797 |
| Burundi | 0.284 | 49.940 |
| Cambodia | 0.469 | 57.079 |
| Cameroon | 0.490 | 42.290 |
| Canada | 0.873 | 90.302 |
| Cape Verde | 0.525 | 62.188 |
| Central African Republic | 0.274 | 22.300 |
| Chad | 0.238 | 31.372 |
| Chile | 0.759 | 74.350 |
| China | 0.686 | 69.712 |
| Colombia | 0.633 | 74.397 |
| Comoros | 0.455 | 48.139 |
| Congo | 0.568 | 43.904 |
| Cook Islands | 0.764 | 62.228 |
| Costa Rica | 0.680 | 79.015 |
| Cote d'Ivoire | 0.408 | 43.040 |
| Croatia | 0.794 | 78.935 |
| Cuba | 0.668 | 72.592 |
| Cyprus | 0.841 | 79.602 |
| Czech Republic | 0.828 | 81.944 |
| Democratic Republic of the Congo | 0.382 | 45.169 |
| Denmark | 0.890 | 84.140 |
| Djibouti | 0.459 | 45.287 |
| Dominica | 0.729 | 51.809 |
| Dominican Republic | 0.592 | 52.500 |
| Ecuador | 0.640 | 64.454 |
| Egypt | 0.658 | 54.797 |
| El Salvador | 0.573 | 61.678 |
| Equatorial Guinea | 0.685 | 49.994 |
| Eritrea | 0.396 | 42.275 |
| Estonia | 0.835 | 82.039 |
| Eswatini | 0.577 | 53.397 |
| Ethiopia | 0.343 | 46.522 |
| Federated States of Micronesia | 0.580 | 34.474 |
| Fiji | 0.664 | 45.176 |
| Finland | 0.856 | 91.349 |
| France | 0.834 | 90.766 |
| Gabon | 0.656 | 53.050 |
| Gambia | 0.399 | 48.065 |
| Georgia | 0.702 | 55.953 |
| Germany | 0.898 | 86.249 |
| Ghana | 0.557 | 49.139 |
| Greece | 0.794 | 80.140 |
| Greenland | 0.761 | 68.725 |
| Grenada | 0.669 | 50.484 |
| Guam | 0.813 | 63.773 |
| Guatemala | 0.526 | 52.099 |
| Guinea | 0.325 | 32.335 |
| Guinea-Bissau | 0.355 | 35.707 |
| Guyana | 0.618 | 40.622 |
| Haiti | 0.432 | 35.812 |
| Honduras | 0.496 | 54.284 |
| Hungary | 0.791 | 72.028 |
| Iceland | 0.869 | 95.307 |
| India | 0.566 | 46.826 |
| Indonesia | 0.660 | 48.728 |
| Iran | 0.670 | 69.515 |
| Iraq | 0.671 | 57.725 |
| Ireland | 0.867 | 90.346 |
| Israel | 0.803 | 81.385 |
| Italy | 0.801 | 88.895 |
| Jamaica | 0.684 | 56.840 |
| Japan | 0.870 | 96.341 |
| Jordan | 0.731 | 69.967 |
| Kazakhstan | 0.723 | 59.237 |
| Kenya | 0.508 | 51.647 |
| Kiribati | 0.527 | 35.736 |
| Kuwait | 0.851 | 81.833 |
| Kyrgyzstan | 0.596 | 52.952 |
| Lao | 0.490 | 43.855 |
| Latvia | 0.820 | 69.788 |
| Lebanon | 0.708 | 74.533 |
| Lesotho | 0.507 | 38.737 |
| Liberia | 0.370 | 47.600 |
| Libya | 0.709 | 66.328 |
| Lithuania | 0.843 | 70.352 |
| Luxembourg | 0.895 | 91.455 |
| Macedonia | 0.744 | 60.746 |
| Madagascar | 0.396 | 39.691 |
| Malawi | 0.384 | 55.521 |
| Malaysia | 0.737 | 66.574 |
| Maldives | 0.562 | 66.857 |
| Mali | 0.263 | 40.661 |
| Malta | 0.801 | 82.881 |
| Marshall Islands | 0.544 | 44.004 |
| Mauritania | 0.496 | 53.278 |
| Mauritius | 0.705 | 55.809 |
| Mexico | 0.649 | 61.437 |
| Moldova | 0.696 | 62.194 |
| Monaco | 0.902 | 91.353 |
| Mongolia | 0.606 | 47.907 |
| Montenegro | 0.791 | 65.959 |
| Morocco | 0.548 | 58.032 |
| Mozambique | 0.307 | 44.044 |
| Myanmar | 0.521 | 46.954 |
| Namibia | 0.612 | 62.169 |
| Nauru | 0.618 | 42.002 |
| Nepal | 0.422 | 47.280 |
| Netherlands | 0.883 | 89.588 |
| New Zealand | 0.840 | 82.978 |
| Nicaragua | 0.517 | 57.159 |
| Niger | 0.162 | 35.026 |
| Nigeria | 0.515 | 38.339 |
| Niue | 0.711 | 49.010 |
| North Korea | 0.558 | 52.838 |
| Northern Mariana Islands | 0.771 | 60.411 |
| Norway | 0.913 | 94.241 |
| Oman | 0.783 | 71.221 |
| Pakistan | 0.449 | 39.168 |
| Palau | 0.738 | 45.067 |
| Palestine | 0.588 | 61.234 |
| Panama | 0.686 | 71.155 |
| Papua New Guinea | 0.394 | 37.767 |
| Paraguay | 0.638 | 63.351 |
| Peru | 0.648 | 75.759 |
| Philippines | 0.623 | 54.712 |
| Poland | 0.802 | 72.656 |
| Portugal | 0.743 | 83.533 |
| Puerto Rico | 0.814 | 75.531 |
| Qatar | 0.830 | 80.403 |
| Romania | 0.760 | 69.585 |
| Russia | 0.805 | 68.974 |
| Rwanda | 0.429 | 59.359 |
| Saint Kitts and Nevis | 0.746 | 52.885 |
| Saint Lucia | 0.670 | 59.140 |
| Saint Vincent and the Grenadines | 0.627 | 49.494 |
| Samoa | 0.641 | 49.796 |
| San Marino | 0.884 | 92.716 |
| Sao Tome and Principe | 0.502 | 54.755 |
| Saudi Arabia | 0.805 | 64.196 |
| Senegal | 0.389 | 49.610 |
| Serbia | 0.767 | 63.349 |
| Seychelles | 0.724 | 61.517 |
| Sierra Leone | 0.347 | 42.120 |
| Singapore | 0.861 | 92.440 |
| Slovakia | 0.812 | 77.983 |
| Slovenia | 0.840 | 89.834 |
| Solomon Islands | 0.407 | 39.333 |
| Somalia | 0.081 | 23.940 |
| South Africa | 0.678 | 59.727 |
| South Korea | 0.878 | 89.162 |
| South Sudan | 0.363 | 41.694 |
| Spain | 0.767 | 90.006 |
| Sri Lanka | 0.690 | 65.563 |
| Sudan | 0.515 | 51.835 |
| Suriname | 0.636 | 50.134 |
| Sweden | 0.872 | 90.361 |
| Switzerland | 0.929 | 93.498 |
| Syrian Arab Republic | 0.619 | 57.565 |
| Taiwan (Province of China) | 0.868 | 79.081 |
| Tajikistan | 0.539 | 47.878 |
| Thailand | 0.423 | 55.249 |
| Timor-Leste | 0.687 | 71.600 |
| Togo | 0.514 | 45.954 |
| Tokelau | 0.417 | 42.809 |
| Tonga | 0.626 | 52.643 |
| Trinidad and Tobago | 0.636 | 52.417 |
| Tunisia | 0.757 | 55.517 |
| Turkey | 0.672 | 68.106 |
| Turkmenistan | 0.748 | 69.210 |
| Tuvalu | 0.670 | 44.013 |
| Uganda | 0.589 | 39.570 |
| Ukraine | 0.404 | 52.748 |
| United Arab Emirates | 0.736 | 56.752 |
| United Kingdom | 0.880 | 63.357 |
| Tanzania | 0.847 | 87.900 |
| United States of America | 0.859 | 82.138 |
| United States Virgin Islands | 0.799 | 53.715 |
| Uruguay | 0.697 | 68.530 |
| Uzbekistan | 0.631 | 42.185 |
| Vanuatu | 0.485 | 34.084 |
| Venezuela | 0.607 | 60.968 |
| Viet Nam | 0.617 | 59.707 |
| Yemen | 0.412 | 49.046 |
| Zambia | 0.505 | 52.694 |
| Zimbabwe | 0.476 | 54.461 |

SDI: socio-demographic index; UHC: universal health coverage.

**Table S2**. The incident cases and ASIRs of prostate cancer in 1990 and 2019 and their temporal trends from 1990 to 2019 at the national level.

| Countries | 1990 | |  | 2019 | |  | 1990-2019 | |
| --- | --- | --- | --- | --- | --- | --- | --- | --- |
|  | Incident cases  No. x 10^3^ (95% UI) | ASIR per 100, 000  No. (95% UI) |  | Incident cases  No. x 10^3^ (95% UI) | ASIR per 100, 000  No. (95% UI) |  | Percentage change  of incident cases (%) | EAPC  No. (95% CI) |
| Afghanistan | 0.36 (0.27, 0.49) | 11.46 (8.33, 15.74) |  | 0.59 (0.44, 0.74) | 13.46 (10.03, 17.53) |  | 62.81 | 0.59 (0.50, 0.69) |
| Albania | 0.19 (0.15, 0.32) | 24.81 (18.46, 41.56) |  | 0.79 (0.49, 1.42) | 38.31 (23.80, 68.00) |  | 309.06 | 1.56 (1.47, 1.65) |
| Algeria | 0.68 (0.50, 0.89) | 13.62 (10.22, 17.58) |  | 2.84 (2.10, 3.96) | 18.01 (13.43, 25.01) |  | 317.8 | 0.72 (0.62, 0.83) |
| American Samoa | 0.00 (0.00, 0.01) | 54.19 (45.10, 69.62) |  | 0.01 (0.01, 0.01) | 62.89 (50.75, 74.46) |  | 171.91 | 0.79 (0.63, 0.94) |
| Andorra | 0.01 (0.01, 0.02) | 53.57 (40.23, 74.37) |  | 0.05 (0.04, 0.08) | 75.13 (54.24, 109.87) |  | 274.23 | 1.12 (0.90, 1.34) |
| Angola | 0.28 (0.19, 0.37) | 23.40 (15.12, 31.45) |  | 1.05 (0.73, 1.35) | 32.15 (22.47, 41.57) |  | 269.62 | 1.07 (1.04, 1.10) |
| Antigua and Barbuda | 0.03 (0.02, 0.04) | 130.26 (109.01, 174.55) |  | 0.08 (0.06, 0.09) | 172.34 (130.59, 215.87) |  | 153.12 | 0.90 (0.51, 1.29) |
| Argentina | 4.64 (4.05, 6.57) | 35.34 (30.82, 50.64) |  | 11.58 (8.56, 16.40) | 49.80 (36.95, 70.33) |  | 149.44 | 0.91 (0.57, 1.25) |
| Armenia | 0.15 (0.11, 0.19) | 14.51 (11.92, 19.42) |  | 0.56 (0.32, 0.70) | 32.31 (18.64, 40.63) |  | 285.65 | 3.02 (2.90, 3.14) |
| Australia | 6.71 (5.14, 8.32) | 80.07 (61.23, 99.04) |  | 21.42 (15.47, 31.97) | 107.10 (77.34, 159.65) |  | 219.3 | 0.51 (0.10, 0.92) |
| Austria | 3.27 (2.45, 4.06) | 72.85 (54.97, 90.88) |  | 6.95 (5.03, 11.18) | 88.68 (64.30, 142.74) |  | 112.21 | 0.27 (-0.05, 0.59) |
| Azerbaijan | 0.24 (0.21, 0.31) | 14.47 (12.24, 19.13) |  | 0.72 (0.56, 0.97) | 21.12 (16.29, 28.63) |  | 197.08 | 1.63 (1.47, 1.78) |
| Bahamas | 0.07 (0.06, 0.09) | 116.57 (96.39, 160.34) |  | 0.22 (0.17, 0.29) | 140.57 (109.20, 190.88) |  | 227.51 | 0.61 (0.41, 0.81) |
| Bahrain | 0.02 (0.01, 0.02) | 23.06 (17.16, 28.77) |  | 0.17 (0.12, 0.24) | 33.78 (25.29, 44.98) |  | 974.75 | 1.31 (1.03, 1.59) |
| Bangladesh | 1.66 (1.17, 2.19) | 7.83 (5.47, 10.40) |  | 5.47 (3.09, 8.43) | 8.87 (5.12, 13.61) |  | 229.85 | 0.28 (0.12, 0.43) |
| Barbados | 0.15 (0.12, 0.19) | 115.94 (92.35, 149.30) |  | 0.43 (0.31, 0.53) | 188.87 (137.07, 232.88) |  | 186.3 | 0.94 (0.60, 1.29) |
| Belarus | 1.36 (1.13, 2.33) | 30.82 (25.51, 53.35) |  | 4.40 (3.21, 6.64) | 73.48 (54.14, 110.84) |  | 222.73 | 3.04 (2.86, 3.22) |
| Belgium | 4.49 (3.30, 5.33) | 72.20 (52.11, 84.01) |  | 8.11 (5.70, 14.11) | 78.35 (54.90, 136.27) |  | 80.58 | 0.03 (-0.31, 0.38) |
| Belize | 0.02 (0.01, 0.03) | 39.49 (31.60, 66.36) |  | 0.09 (0.07, 0.11) | 68.27 (54.07, 90.02) |  | 399.96 | 1.46 (0.91, 2.02) |
| Benin | 0.16 (0.13, 0.22) | 19.12 (15.51, 25.86) |  | 0.55 (0.33, 0.74) | 32.32 (19.42, 42.72) |  | 242.48 | 2.11 (1.92, 2.30) |
| Bermuda | 0.03 (0.02, 0.03) | 111.57 (76.69, 135.99) |  | 0.11 (0.08, 0.14) | 183.27 (141.15, 240.47) |  | 288.44 | 1.68 (1.49, 1.88) |
| Bhutan | 0.01 (0.00, 0.01) | 7.06 (4.13, 10.48) |  | 0.03 (0.02, 0.05) | 11.83 (6.57, 18.83) |  | 459.19 | 1.98 (1.89, 2.07) |
| Bolivia | 0.37 (0.29, 0.45) | 30.95 (24.74, 38.50) |  | 2.00 (1.45, 2.63) | 54.47 (39.97, 70.22) |  | 448.02 | 1.90 (1.81, 1.99) |
| Bosnia and Herzegovina | 0.24 (0.20, 0.30) | 17.24 (14.88, 22.23) |  | 0.89 (0.60, 1.19) | 33.93 (22.90, 45.10) |  | 275.01 | 2.85 (2.51, 3.19) |
| Botswana | 0.09 (0.07, 0.11) | 44.96 (33.98, 57.05) |  | 0.32 (0.22, 0.42) | 70.87 (49.16, 91.07) |  | 266.69 | 1.07 (0.65, 1.49) |
| Brazil | 13.05 (11.62, 19.94) | 37.04 (32.52, 56.11) |  | 55.69 (48.42, 82.97) | 55.03 (47.74, 81.83) |  | 326.93 | 1.22 (0.88, 1.56) |
| Brunei | 0.01 (0.01, 0.01) | 23.86 (19.11, 30.15) |  | 0.03 (0.02, 0.04) | 39.69 (25.92, 51.07) |  | 369.49 | 2.66 (2.27, 3.05) |
| Bulgaria | 1.14 (0.98, 1.83) | 20.77 (17.85, 33.55) |  | 2.44 (1.73, 3.17) | 38.17 (27.07, 49.28) |  | 114.77 | 2.94 (2.64, 3.24) |
| Burkina Faso | 0.30 (0.23, 0.40) | 18.00 (14.17, 24.60) |  | 0.97 (0.60, 1.29) | 30.86 (18.94, 40.46) |  | 227.37 | 2.22 (2.09, 2.34) |
| Burundi | 0.23 (0.12, 0.31) | 25.30 (13.33, 34.13) |  | 0.48 (0.29, 0.68) | 26.43 (16.50, 37.05) |  | 108.02 | 0.14 (0.07, 0.21) |
| Cambodia | 0.19 (0.14, 0.24) | 13.49 (10.12, 16.87) |  | 0.89 (0.63, 1.11) | 23.21 (16.61, 28.87) |  | 359.14 | 1.99 (1.87, 2.10) |
| Cameroon | 0.34 (0.27, 0.47) | 20.79 (16.13, 28.59) |  | 1.65 (0.89, 2.47) | 38.48 (21.07, 57.28) |  | 381.64 | 2.33 (2.17, 2.48) |
| Canada | 11.45 (8.52, 13.56) | 80.47 (59.73, 94.79) |  | 23.29 (16.34, 35.62) | 71.40 (50.15, 109.16) |  | 103.36 | -1.14 (-1.52, -0.77) |
| Cape Verde | 0.02 (0.02, 0.04) | 21.91 (16.98, 33.87) |  | 0.15 (0.10, 0.18) | 94.35 (62.83, 116.02) |  | 558.82 | 3.63 (2.86, 4.42) |
| Central African Republic | 0.09 (0.06, 0.12) | 26.53 (17.79, 36.07) |  | 0.15 (0.10, 0.20) | 27.41 (18.83, 36.84) |  | 68.81 | 0.14 (0.08, 0.19) |
| Chad | 0.18 (0.14, 0.26) | 15.25 (11.33, 21.70) |  | 0.66 (0.41, 0.89) | 27.77 (17.47, 37.17) |  | 260 | 2.42 (2.23, 2.62) |
| Chile | 1.28 (1.05, 1.67) | 32.23 (26.57, 42.36) |  | 6.40 (4.55, 8.76) | 60.31 (42.83, 82.56) |  | 399.87 | 2.14 (1.85, 2.43) |
| China | 26.44 (20.10, 31.92) | 8.88 (7.14, 10.86) |  | 153.45 (118.40, 204.94) | 17.34 (13.62, 22.72) |  | 480.37 | 2.54 (2.44, 2.64) |
| Colombia | 2.91 (2.33, 3.80) | 38.79 (31.27, 51.00) |  | 14.73 (10.35, 23.06) | 62.38 (44.05, 97.39) |  | 405.9 | 1.09 (0.76, 1.43) |
| Comoros | 0.02 (0.01, 0.03) | 25.85 (14.26, 36.04) |  | 0.06 (0.04, 0.08) | 30.89 (20.32, 41.29) |  | 145.01 | 0.58 (0.50, 0.65) |
| Congo | 0.10 (0.07, 0.12) | 30.52 (21.84, 37.65) |  | 0.28 (0.20, 0.36) | 33.98 (23.59, 42.78) |  | 183.75 | 0.33 (0.16, 0.50) |
| Cook Islands | 0.01 (0.00, 0.01) | 99.72 (82.26, 121.97) |  | 0.01 (0.01, 0.02) | 110.47 (88.54, 133.33) |  | 138.42 | 0.19 (0.12, 0.27) |
| Costa Rica | 0.36 (0.30, 0.49) | 45.23 (37.26, 61.11) |  | 2.09 (1.31, 2.88) | 90.08 (56.34, 122.71) |  | 483.15 | 2.36 (2.08, 2.64) |
| Cote d'Ivoire | 0.29 (0.23, 0.39) | 20.34 (16.18, 27.35) |  | 1.25 (0.71, 1.80) | 32.64 (18.73, 46.12) |  | 333.07 | 1.88 (1.72, 2.04) |
| Croatia | 1.03 (0.80, 1.29) | 44.86 (34.26, 56.48) |  | 2.55 (1.79, 3.39) | 66.54 (46.58, 88.22) |  | 148.6 | 1.81 (1.60, 2.03) |
| Cuba | 3.12 (2.56, 4.04) | 62.13 (51.54, 80.62) |  | 10.01 (5.99, 12.85) | 111.54 (67.03, 143.68) |  | 221.25 | 2.02 (1.92, 2.12) |
| Cyprus | 0.15 (0.12, 0.19) | 39.21 (32.03, 51.80) |  | 0.81 (0.63, 1.06) | 85.14 (67.26, 110.30) |  | 443.57 | 2.92 (2.44, 3.41) |
| Czech Republic | 2.18 (1.71, 2.94) | 40.08 (31.68, 54.00) |  | 6.23 (4.48, 8.15) | 65.11 (47.09, 84.19) |  | 185.62 | 1.71 (1.25, 2.17) |
| Democratic Republic of the Congo | 1.20 (0.85, 1.63) | 23.87 (16.44, 32.62) |  | 2.84 (1.93, 3.80) | 27.32 (18.36, 36.69) |  | 135.42 | 0.39 (0.33, 0.45) |
| Denmark | 1.87 (1.44, 2.27) | 52.90 (41.03, 64.47) |  | 4.54 (3.23, 6.04) | 81.63 (58.46, 108.53) |  | 142.35 | 2.26 (1.76, 2.76) |
| Djibouti | 0.01 (0.01, 0.02) | 27.19 (15.77, 37.86) |  | 0.09 (0.06, 0.12) | 36.16 (23.07, 49.74) |  | 576.72 | 0.99 (0.94, 1.05) |
| Dominica | 0.05 (0.04, 0.06) | 153.07 (129.99, 199.97) |  | 0.08 (0.06, 0.10) | 195.69 (144.17, 244.11) |  | 80.15 | 0.72 (0.47, 0.97) |
| Dominican Republic | 0.59 (0.40, 1.08) | 37.59 (25.44, 67.66) |  | 3.03 (1.95, 4.98) | 73.23 (47.60, 120.72) |  | 409.47 | 2.44 (1.85, 3.02) |
| Ecuador | 0.61 (0.53, 0.79) | 26.81 (23.11, 34.64) |  | 3.21 (2.38, 4.30) | 48.48 (35.96, 64.30) |  | 427.99 | 2.23 (1.94, 2.51) |
| Egypt | 0.86 (0.73, 1.06) | 7.23 (6.14, 9.29) |  | 3.80 (2.62, 5.43) | 12.01 (8.52, 16.97) |  | 343.8 | 1.53 (1.34, 1.72) |
| El Salvador | 0.25 (0.21, 0.36) | 19.72 (16.13, 28.61) |  | 1.32 (0.93, 1.77) | 52.23 (36.66, 70.39) |  | 426.01 | 2.92 (2.35, 3.49) |
| Equatorial Guinea | 0.01 (0.01, 0.02) | 23.03 (13.98, 32.11) |  | 0.06 (0.04, 0.08) | 39.21 (26.04, 51.23) |  | 308.68 | 2.02 (1.91, 2.13) |
| Eritrea | 0.06 (0.03, 0.09) | 23.98 (9.48, 34.39) |  | 0.23 (0.13, 0.31) | 29.73 (16.42, 40.34) |  | 259.53 | 0.46 (0.22, 0.71) |
| Estonia | 0.32 (0.23, 0.37) | 45.82 (33.18, 53.36) |  | 1.20 (0.65, 1.60) | 119.18 (65.00, 159.57) |  | 277.28 | 4.31 (3.92, 4.71) |
| Eswatini | 0.04 (0.03, 0.05) | 42.09 (32.99, 51.75) |  | 0.09 (0.07, 0.12) | 57.24 (40.59, 72.75) |  | 151.36 | 1.14 (0.95, 1.33) |
| Ethiopia | 0.87 (0.61, 1.25) | 10.69 (7.24, 14.94) |  | 2.57 (1.35, 4.30) | 14.52 (7.70, 23.87) |  | 195.82 | 1.13 (0.97, 1.29) |
| Federated States of Micronesia | 0.00 (0.00, 0.01) | 28.38 (21.15, 41.20) |  | 0.01 (0.01, 0.01) | 38.46 (27.74, 50.07) |  | 78.69 | 1.10 (1.00, 1.19) |
| Fiji | 0.03 (0.02, 0.04) | 23.83 (12.91, 33.78) |  | 0.07 (0.03, 0.11) | 29.59 (13.72, 42.21) |  | 151.86 | 0.96 (0.79, 1.13) |
| Finland | 1.60 (1.21, 1.93) | 61.36 (46.71, 73.84) |  | 6.10 (4.38, 8.92) | 107.45 (77.12, 156.79) |  | 282.04 | 1.50 (0.97, 2.03) |
| France | 22.90 (16.04, 26.54) | 67.57 (46.98, 77.92) |  | 44.73 (31.59, 74.05) | 73.62 (51.88, 121.61) |  | 95.34 | 0.08 (-0.27, 0.44) |
| Gabon | 0.06 (0.04, 0.07) | 30.95 (21.79, 38.54) |  | 0.15 (0.10, 0.21) | 42.72 (27.72, 56.49) |  | 161.82 | 1.04 (0.98, 1.10) |
| Gambia | 0.01 (0.01, 0.02) | 8.37 (6.59, 11.34) |  | 0.04 (0.03, 0.06) | 11.45 (8.44, 15.34) |  | 284.68 | 0.98 (0.87, 1.09) |
| Georgia | 0.44 (0.35, 0.57) | 19.96 (16.35, 26.99) |  | 0.81 (0.47, 1.02) | 33.57 (19.69, 41.86) |  | 83.81 | 2.94 (2.34, 3.54) |
| Germany | 27.80 (20.86, 34.38) | 59.63 (45.22, 74.05) |  | 75.38 (53.64, 104.00) | 87.61 (61.70, 120.88) |  | 171.18 | 0.92 (0.57, 1.28) |
| Ghana | 0.95 (0.68, 1.26) | 43.28 (31.32, 55.30) |  | 2.72 (2.15, 3.54) | 49.24 (39.65, 64.27) |  | 185.04 | 0.07 (-0.14, 0.28) |
| Greece | 3.43 (2.84, 4.68) | 49.17 (40.68, 66.95) |  | 6.93 (5.15, 10.40) | 62.24 (45.54, 95.65) |  | 101.85 | 0.40 (0.03, 0.76) |
| Greenland | 0.00 (0.00, 0.00) | 18.27 (14.63, 26.80) |  | 0.01 (0.01, 0.01) | 23.74 (18.68, 29.85) |  | 263.72 | 0.85 (0.74, 0.95) |
| Grenada | 0.03 (0.03, 0.05) | 106.12 (88.55, 149.49) |  | 0.09 (0.06, 0.10) | 181.85 (122.16, 215.10) |  | 157.11 | 1.58 (0.81, 2.36) |
| Guam | 0.01 (0.01, 0.01) | 31.82 (25.95, 40.93) |  | 0.02 (0.02, 0.03) | 28.82 (23.02, 36.77) |  | 185.03 | -0.34 (-0.53, -0.15) |
| Guatemala | 0.27 (0.19, 0.63) | 19.80 (13.84, 44.93) |  | 2.25 (1.66, 3.14) | 50.26 (37.24, 67.24) |  | 718.23 | 3.06 (2.28, 3.84) |
| Guinea | 0.38 (0.22, 0.50) | 26.93 (15.90, 35.26) |  | 0.83 (0.44, 1.26) | 35.74 (19.00, 53.79) |  | 116.76 | 1.21 (1.06, 1.36) |
| Guinea-Bissau | 0.03 (0.02, 0.05) | 20.66 (15.43, 28.96) |  | 0.08 (0.04, 0.12) | 34.30 (19.02, 49.81) |  | 147.44 | 2.12 (1.97, 2.28) |
| Guyana | 0.10 (0.08, 0.15) | 68.82 (56.02, 98.79) |  | 0.21 (0.16, 0.30) | 88.74 (66.92, 124.03) |  | 107.16 | 0.57 (0.39, 0.76) |
| Haiti | 0.64 (0.45, 0.83) | 55.79 (39.04, 73.03) |  | 1.66 (1.15, 2.34) | 65.02 (45.03, 89.33) |  | 160.67 | 0.51 (0.47, 0.54) |
| Honduras | 0.17 (0.13, 0.24) | 19.76 (14.21, 27.67) |  | 1.02 (0.73, 1.51) | 39.72 (28.39, 58.69) |  | 497.36 | 2.93 (2.62, 3.25) |
| Hungary | 2.03 (1.61, 2.61) | 35.46 (27.76, 45.07) |  | 3.80 (2.89, 5.04) | 47.66 (36.59, 62.89) |  | 87.03 | 0.73 (0.46, 0.99) |
| Iceland | 0.11 (0.08, 0.13) | 80.78 (61.60, 95.55) |  | 0.24 (0.18, 0.32) | 88.90 (68.39, 117.32) |  | 124.53 | 0.20 (-0.20, 0.59) |
| India | 11.57 (8.56, 13.60) | 7.52 (5.58, 8.88) |  | 41.60 (33.78, 53.92) | 8.91 (7.24, 11.62) |  | 259.65 | 0.45 (0.36, 0.54) |
| Indonesia | 3.55 (2.77, 4.27) | 10.32 (8.12, 12.49) |  | 16.13 (11.41, 20.22) | 20.98 (15.07, 26.28) |  | 354.54 | 2.52 (2.45, 2.59) |
| Iran | 1.78 (1.35, 2.19) | 15.84 (12.20, 19.63) |  | 10.42 (7.30, 12.19) | 30.01 (21.34, 34.71) |  | 486.03 | 2.01 (1.80, 2.22) |
| Iraq | 0.36 (0.25, 0.50) | 10.89 (7.67, 15.16) |  | 1.92 (1.43, 2.47) | 19.94 (14.95, 26.31) |  | 436.58 | 1.98 (1.74, 2.23) |
| Ireland | 1.12 (0.80, 1.34) | 60.01 (43.80, 72.50) |  | 3.15 (2.22, 4.56) | 87.67 (61.87, 126.62) |  | 182.33 | 1.20 (0.79, 1.61) |
| Israel | 0.72 (0.60, 0.97) | 32.05 (26.65, 43.24) |  | 2.24 (1.54, 3.86) | 41.95 (28.96, 72.56) |  | 211.39 | 0.50 (0.07, 0.93) |
| Italy | 20.39 (16.62, 26.78) | 52.86 (43.71, 69.59) |  | 41.89 (30.61, 63.05) | 66.95 (48.64, 102.23) |  | 105.51 | 0.62 (0.24, 1.01) |
| Jamaica | 0.49 (0.39, 0.62) | 59.29 (47.41, 74.39) |  | 1.76 (0.96, 2.34) | 126.82 (69.64, 167.06) |  | 256.99 | 2.63 (1.91, 3.34) |
| Japan | 13.47 (11.07, 18.57) | 20.64 (16.90, 28.32) |  | 56.35 (41.96, 77.76) | 32.76 (24.32, 45.47) |  | 318.25 | 2.10 (1.70, 2.51) |
| Jordan | 0.07 (0.06, 0.10) | 14.03 (10.92, 17.92) |  | 0.74 (0.52, 1.00) | 24.51 (17.11, 32.42) |  | 902.43 | 2.22 (1.96, 2.47) |
| Kazakhstan | 0.57 (0.42, 0.72) | 14.45 (10.73, 18.66) |  | 1.30 (1.00, 1.73) | 20.97 (16.15, 27.88) |  | 127.08 | 1.97 (1.63, 2.31) |
| Kenya | 0.56 (0.29, 1.00) | 16.80 (8.81, 29.75) |  | 2.35 (1.54, 3.42) | 29.88 (20.21, 41.54) |  | 321.69 | 2.32 (2.10, 2.54) |
| Kiribati | 0.00 (0.00, 0.00) | 17.58 (12.92, 24.52) |  | 0.00 (0.00, 0.00) | 16.74 (12.53, 22.71) |  | 56.7 | -0.26 (-0.43, -0.09) |
| Kuwait | 0.05 (0.04, 0.07) | 16.06 (12.36, 20.44) |  | 0.43 (0.31, 0.60) | 31.18 (23.00, 42.87) |  | 705.8 | 2.40 (2.12, 2.68) |
| Kyrgyzstan | 0.12 (0.06, 0.15) | 11.83 (5.84, 14.03) |  | 0.18 (0.13, 0.22) | 11.28 (7.95, 13.85) |  | 46.53 | -0.40 (-0.70, -0.10) |
| Lao | 0.09 (0.06, 0.11) | 11.82 (8.40, 15.10) |  | 0.25 (0.18, 0.32) | 16.03 (11.53, 19.86) |  | 191.1 | 0.95 (0.86, 1.03) |
| Latvia | 0.43 (0.34, 0.55) | 35.79 (27.88, 45.31) |  | 1.10 (0.65, 1.47) | 75.15 (44.49, 99.39) |  | 154.46 | 3.47 (3.04, 3.89) |
| Lebanon | 0.29 (0.23, 0.37) | 30.39 (23.09, 38.27) |  | 1.72 (1.11, 2.42) | 73.79 (47.89, 103.42) |  | 485.26 | 3.60 (3.43, 3.78) |
| Lesotho | 0.12 (0.09, 0.16) | 37.45 (27.88, 48.32) |  | 0.22 (0.16, 0.28) | 58.31 (43.67, 71.77) |  | 83.91 | 1.53 (1.35, 1.70) |
| Liberia | 0.10 (0.07, 0.13) | 18.52 (14.39, 25.96) |  | 0.24 (0.14, 0.33) | 30.38 (18.47, 41.96) |  | 147.68 | 2.16 (1.93, 2.40) |
| Libya | 0.11 (0.07, 0.15) | 12.96 (8.17, 17.57) |  | 0.45 (0.33, 0.61) | 19.56 (14.20, 26.08) |  | 308.28 | 1.64 (1.46, 1.82) |
| Lithuania | 0.80 (0.65, 1.09) | 48.34 (38.80, 64.80) |  | 2.26 (1.26, 2.90) | 105.69 (58.95, 135.55) |  | 182.15 | 3.51 (2.89, 4.14) |
| Luxembourg | 0.13 (0.09, 0.16) | 62.22 (42.40, 72.32) |  | 0.30 (0.22, 0.51) | 65.36 (47.78, 111.23) |  | 125.23 | -0.06 (-0.37, 0.25) |
| Macedonia | 0.16 (0.12, 0.26) | 20.07 (14.31, 33.77) |  | 0.62 (0.46, 0.83) | 41.71 (31.54, 55.42) |  | 288.13 | 2.85 (2.64, 3.07) |
| Madagascar | 0.49 (0.28, 0.66) | 22.32 (12.66, 29.95) |  | 0.90 (0.53, 1.30) | 23.32 (13.86, 33.07) |  | 83.9 | -0.05 (-0.19, 0.09) |
| Malawi | 0.21 (0.15, 0.32) | 14.63 (10.56, 22.50) |  | 0.52 (0.40, 0.73) | 20.00 (15.75, 28.50) |  | 148.67 | 1.30 (1.16, 1.45) |
| Malaysia | 0.42 (0.32, 0.51) | 11.44 (8.68, 14.08) |  | 2.39 (1.66, 3.16) | 20.17 (14.04, 26.65) |  | 466.43 | 1.66 (1.49, 1.83) |
| Maldives | 0.00 (0.00, 0.00) | 8.14 (5.96, 11.88) |  | 0.02 (0.02, 0.03) | 16.40 (12.54, 21.89) |  | 623.28 | 2.51 (2.43, 2.60) |
| Mali | 0.16 (0.13, 0.21) | 10.33 (8.31, 13.00) |  | 0.50 (0.38, 0.64) | 13.75 (10.56, 17.78) |  | 201.85 | 1.02 (0.95, 1.09) |
| Malta | 0.08 (0.06, 0.10) | 42.73 (33.62, 55.58) |  | 0.24 (0.18, 0.38) | 53.29 (40.80, 83.66) |  | 206.54 | 0.75 (0.49, 1.00) |
| Marshall Islands | 0.00 (0.00, 0.00) | 32.41 (22.42, 46.38) |  | 0.00 (0.00, 0.00) | 32.57 (24.58, 42.02) |  | 102.89 | 0.28 (0.04, 0.53) |
| Mauritania | 0.08 (0.06, 0.11) | 19.90 (14.94, 27.40) |  | 0.29 (0.18, 0.38) | 32.37 (20.25, 42.38) |  | 273.31 | 2.01 (1.86, 2.16) |
| Mauritius | 0.04 (0.04, 0.07) | 16.77 (14.14, 24.94) |  | 0.20 (0.13, 0.26) | 27.68 (18.44, 35.36) |  | 349.07 | 1.09 (0.67, 1.52) |
| Mexico | 5.58 (4.56, 7.27) | 30.57 (25.31, 40.40) |  | 27.10 (20.60, 36.02) | 52.35 (40.04, 70.09) |  | 385.3 | 1.49 (1.20, 1.78) |
| Moldova | 0.31 (0.27, 0.44) | 18.84 (16.16, 26.77) |  | 0.87 (0.59, 1.07) | 36.38 (25.01, 45.35) |  | 174.85 | 3.34 (2.77, 3.91) |
| Monaco | 0.02 (0.02, 0.03) | 65.44 (49.51, 86.83) |  | 0.04 (0.03, 0.05) | 86.17 (66.99, 119.58) |  | 90.94 | 1.09 (0.90, 1.28) |
| Mongolia | 0.03 (0.02, 0.04) | 8.13 (6.42, 11.22) |  | 0.08 (0.06, 0.11) | 10.53 (8.31, 13.94) |  | 155.78 | 0.80 (0.71, 0.88) |
| Montenegro | 0.09 (0.07, 0.12) | 36.38 (29.26, 46.56) |  | 0.25 (0.19, 0.32) | 56.21 (43.19, 71.38) |  | 176.07 | 1.83 (1.66, 2.01) |
| Morocco | 0.48 (0.36, 0.61) | 8.66 (6.31, 11.38) |  | 2.13 (1.52, 2.79) | 15.46 (11.12, 20.20) |  | 340.94 | 1.68 (1.29, 2.06) |
| Mozambique | 0.18 (0.13, 0.28) | 8.70 (6.63, 12.91) |  | 0.45 (0.31, 0.77) | 13.06 (9.26, 20.46) |  | 154.02 | 1.57 (1.50, 1.64) |
| Myanmar | 0.94 (0.70, 1.20) | 11.65 (8.85, 14.61) |  | 2.83 (2.03, 3.55) | 17.54 (12.68, 21.74) |  | 200.19 | 1.45 (1.38, 1.51) |
| Namibia | 0.07 (0.05, 0.10) | 25.57 (18.01, 38.33) |  | 0.26 (0.18, 0.34) | 52.51 (36.30, 66.25) |  | 262.83 | 2.93 (2.77, 3.10) |
| Nauru | 0.00 (0.00, 0.00) | 33.70 (25.80, 45.27) |  | 0.00 (0.00, 0.00) | 46.06 (31.09, 65.41) |  | 19.76 | 0.97 (0.90, 1.03) |
| Nepal | 0.21 (0.13, 0.30) | 6.29 (3.76, 8.88) |  | 0.87 (0.51, 1.32) | 9.80 (5.88, 14.72) |  | 310.98 | 1.55 (1.40, 1.71) |
| Netherlands | 5.80 (4.41, 7.26) | 70.39 (53.27, 87.72) |  | 16.37 (11.51, 22.21) | 99.92 (70.46, 134.84) |  | 182.16 | 1.00 (0.75, 1.25) |
| New Zealand | 2.04 (1.46, 2.48) | 116.09 (83.30, 140.13) |  | 4.28 (3.10, 6.44) | 114.78 (83.50, 173.43) |  | 109.8 | -0.66 (-0.89, -0.42) |
| Nicaragua | 0.21 (0.17, 0.28) | 36.24 (29.16, 49.27) |  | 1.41 (1.06, 1.88) | 80.33 (60.81, 106.94) |  | 569.58 | 2.85 (2.71, 2.98) |
| Niger | 0.16 (0.12, 0.21) | 15.36 (11.83, 21.20) |  | 0.75 (0.47, 1.07) | 26.93 (16.71, 37.72) |  | 383.62 | 2.36 (2.19, 2.54) |
| Nigeria | 6.24 (3.00, 9.42) | 37.85 (18.92, 55.51) |  | 17.66 (7.84, 26.69) | 54.79 (24.85, 81.17) |  | 182.82 | 1.61 (1.44, 1.79) |
| Niue | 0.00 (0.00, 0.00) | 35.14 (28.74, 46.54) |  | 0.00 (0.00, 0.00) | 55.56 (41.49, 67.99) |  | 62.3 | 1.80 (1.69, 1.92) |
| North Korea | 0.53 (0.38, 0.70) | 12.21 (8.55, 16.18) |  | 1.51 (1.13, 1.85) | 13.90 (10.43, 17.01) |  | 185.92 | 0.59 (0.43, 0.74) |
| Northern Mariana Islands | 0.00 (0.00, 0.00) | 32.62 (25.26, 48.20) |  | 0.01 (0.01, 0.01) | 52.82 (42.56, 63.98) |  | 443.39 | 1.76 (1.47, 2.06) |
| Norway | 2.17 (1.59, 2.55) | 70.99 (52.13, 83.49) |  | 4.82 (3.56, 6.75) | 104.82 (77.10, 147.67) |  | 122.15 | 1.38 (0.96, 1.81) |
| Oman | 0.04 (0.03, 0.06) | 15.79 (10.61, 21.96) |  | 0.25 (0.18, 0.34) | 29.64 (23.69, 39.13) |  | 488.43 | 2.17 (1.86, 2.48) |
| Pakistan | 2.76 (1.99, 3.76) | 9.93 (7.19, 13.78) |  | 5.97 (4.20, 7.97) | 13.50 (9.87, 17.73) |  | 116.08 | 1.08 (0.96, 1.20) |
| Palau | 0.00 (0.00, 0.00) | 48.74 (37.56, 65.54) |  | 0.01 (0.00, 0.01) | 60.37 (46.66, 78.93) |  | 157.43 | 0.69 (0.64, 0.74) |
| Palestine | 0.08 (0.06, 0.11) | 23.72 (16.18, 32.88) |  | 0.29 (0.20, 0.38) | 31.48 (20.46, 40.59) |  | 260.68 | 0.81 (0.64, 0.99) |
| Panama | 0.28 (0.23, 0.38) | 39.84 (33.09, 53.45) |  | 1.51 (1.01, 2.07) | 76.43 (51.45, 105.01) |  | 432.61 | 1.63 (1.15, 2.11) |
| Papua New Guinea | 0.11 (0.07, 0.16) | 16.96 (10.57, 25.34) |  | 0.39 (0.26, 0.54) | 23.81 (16.03, 32.32) |  | 258.64 | 1.24 (1.18, 1.29) |
| Paraguay | 0.22 (0.18, 0.29) | 23.45 (19.16, 31.13) |  | 1.25 (0.78, 1.72) | 51.21 (32.34, 70.18) |  | 469.12 | 2.44 (2.08, 2.81) |
| Peru | 1.32 (1.07, 1.77) | 26.03 (20.98, 35.18) |  | 6.88 (4.89, 9.62) | 46.03 (32.67, 64.43) |  | 421.61 | 2.33 (2.15, 2.50) |
| Philippines | 2.20 (1.67, 2.67) | 19.47 (14.87, 23.45) |  | 6.39 (4.31, 8.85) | 22.46 (15.28, 30.46) |  | 190.75 | 0.26 (0.03, 0.49) |
| Poland | 3.61 (3.17, 5.19) | 22.06 (19.56, 32.46) |  | 12.48 (7.91, 16.09) | 41.98 (26.58, 54.11) |  | 245.89 | 2.39 (2.07, 2.72) |
| Portugal | 3.16 (2.43, 3.89) | 54.24 (41.09, 65.83) |  | 8.45 (6.07, 13.12) | 81.03 (57.86, 126.05) |  | 167.04 | 0.96 (0.69, 1.24) |
| Puerto Rico | 1.15 (0.84, 1.38) | 69.02 (49.99, 81.72) |  | 2.78 (2.01, 4.26) | 84.46 (60.78, 129.90) |  | 141.92 | 0.54 (0.40, 0.68) |
| Qatar | 0.01 (0.01, 0.01) | 19.61 (14.66, 26.18) |  | 0.22 (0.14, 0.36) | 42.35 (29.92, 58.12) |  | 2575.28 | 3.23 (2.93, 3.53) |
| Romania | 2.09 (1.79, 2.99) | 18.05 (15.62, 26.59) |  | 6.86 (4.46, 8.66) | 42.28 (27.38, 53.20) |  | 228.58 | 3.10 (2.96, 3.24) |
| Russia | 13.02 (11.35, 19.44) | 21.89 (19.08, 33.45) |  | 45.09 (29.27, 57.03) | 49.07 (31.92, 61.61) |  | 246.29 | 3.47 (3.21, 3.74) |
| Rwanda | 0.29 (0.15, 0.40) | 27.31 (14.40, 37.57) |  | 0.66 (0.43, 0.90) | 34.25 (22.38, 45.96) |  | 132.33 | 0.46 (0.27, 0.66) |
| Saint Kitts and Nevis | 0.02 (0.02, 0.03) | 134.82 (110.54, 179.24) |  | 0.07 (0.05, 0.08) | 235.26 (184.24, 287.38) |  | 189.3 | 1.32 (1.05, 1.59) |
| Saint Lucia | 0.04 (0.03, 0.05) | 109.69 (92.27, 145.11) |  | 0.14 (0.11, 0.19) | 146.97 (118.26, 200.59) |  | 266.17 | 0.46 (0.23, 0.69) |
| Saint Vincent and the Grenadines | 0.03 (0.02, 0.04) | 95.27 (79.94, 143.24) |  | 0.08 (0.07, 0.11) | 129.92 (102.99, 160.75) |  | 201.11 | 0.66 (0.43, 0.89) |
| Samoa | 0.01 (0.01, 0.01) | 20.42 (15.82, 24.84) |  | 0.01 (0.01, 0.01) | 19.92 (15.34, 24.08) |  | 66.47 | -0.14 (-0.27, 0.00) |
| San Marino | 0.01 (0.01, 0.01) | 65.99 (52.98, 82.98) |  | 0.03 (0.02, 0.04) | 90.30 (62.71, 138.15) |  | 181.74 | 1.09 (1.00, 1.18) |
| Sao Tome and Principe | 0.00 (0.00, 0.01) | 16.24 (12.83, 21.37) |  | 0.01 (0.01, 0.01) | 27.01 (20.24, 35.77) |  | 170.9 | 2.02 (1.86, 2.19) |
| Saudi Arabia | 0.34 (0.20, 0.53) | 12.86 (7.51, 20.47) |  | 2.30 (1.66, 3.46) | 23.41 (17.23, 34.96) |  | 574.65 | 1.60 (1.36, 1.84) |
| Senegal | 0.27 (0.20, 0.38) | 20.40 (15.70, 28.47) |  | 1.07 (0.67, 1.43) | 36.35 (22.40, 47.88) |  | 290.68 | 2.39 (2.18, 2.60) |
| Serbia | 1.03 (0.83, 1.30) | 22.00 (17.75, 28.11) |  | 3.26 (2.28, 4.29) | 43.13 (30.45, 55.92) |  | 216.5 | 2.87 (2.61, 3.13) |
| Seychelles | 0.01 (0.01, 0.02) | 47.70 (34.45, 75.12) |  | 0.04 (0.03, 0.05) | 96.89 (71.33, 119.68) |  | 298.29 | 2.19 (1.80, 2.58) |
| Sierra Leone | 0.16 (0.12, 0.22) | 17.71 (13.79, 24.89) |  | 0.42 (0.26, 0.59) | 29.58 (18.32, 40.87) |  | 169.9 | 2.21 (2.03, 2.40) |
| Singapore | 0.13 (0.10, 0.17) | 16.29 (12.84, 21.76) |  | 0.99 (0.70, 1.35) | 28.21 (20.04, 38.04) |  | 651.07 | 2.04 (1.78, 2.30) |
| Slovakia | 0.74 (0.63, 0.91) | 30.44 (26.18, 38.02) |  | 2.16 (1.39, 2.93) | 56.75 (36.65, 75.96) |  | 193.49 | 2.55 (2.36, 2.73) |
| Slovenia | 0.35 (0.25, 0.50) | 38.26 (27.81, 54.05) |  | 1.49 (0.97, 2.08) | 77.91 (50.55, 107.94) |  | 328.54 | 2.92 (2.57, 3.27) |
| Solomon Islands | 0.01 (0.01, 0.02) | 23.48 (15.95, 34.20) |  | 0.03 (0.03, 0.04) | 31.31 (24.06, 39.19) |  | 168.6 | 0.99 (0.91, 1.07) |
| Somalia | 0.20 (0.10, 0.29) | 24.08 (12.72, 35.07) |  | 0.48 (0.29, 0.72) | 24.52 (14.67, 36.49) |  | 145.99 | 0.14 (0.08, 0.20) |
| South Africa | 2.63 (1.98, 3.43) | 36.54 (27.59, 46.73) |  | 7.78 (6.29, 9.37) | 50.82 (40.40, 59.81) |  | 195.69 | 1.18 (1.00, 1.36) |
| South Korea | 1.02 (0.88, 1.38) | 11.83 (10.18, 16.55) |  | 10.61 (7.92, 14.19) | 27.87 (20.98, 36.35) |  | 944.75 | 3.46 (3.04, 3.88) |
| South Sudan | 0.26 (0.15, 0.37) | 23.83 (13.44, 33.50) |  | 0.39 (0.25, 0.56) | 24.83 (15.67, 34.88) |  | 52.25 | 0.14 (0.11, 0.18) |
| Spain | 9.59 (7.13, 11.54) | 42.11 (31.24, 50.85) |  | 25.60 (18.62, 39.87) | 60.05 (43.53, 93.66) |  | 166.95 | 0.88 (0.45, 1.32) |
| Sri Lanka | 0.42 (0.36, 0.54) | 9.48 (7.97, 12.17) |  | 1.58 (1.11, 2.20) | 14.92 (10.72, 20.47) |  | 273.77 | 2.03 (1.85, 2.21) |
| Sudan | 0.39 (0.27, 0.54) | 9.50 (6.64, 13.16) |  | 1.29 (0.89, 1.75) | 14.87 (10.37, 19.86) |  | 231.96 | 1.44 (1.34, 1.54) |
| Suriname | 0.04 (0.04, 0.06) | 41.90 (34.72, 58.67) |  | 0.16 (0.12, 0.20) | 65.34 (48.41, 81.63) |  | 260.93 | 1.52 (1.33, 1.70) |
| Sweden | 5.63 (4.15, 6.66) | 81.85 (60.28, 96.72) |  | 9.78 (7.39, 13.30) | 97.09 (72.44, 132.05) |  | 73.75 | 0.23 (-0.14, 0.61) |
| Switzerland | 4.37 (2.73, 4.95) | 100.31 (62.54, 113.63) |  | 7.35 (5.16, 12.08) | 91.34 (63.70, 151.37) |  | 68.46 | -0.45 (-0.68, -0.22) |
| Syrian Arab Republic | 0.28 (0.19, 0.43) | 11.72 (7.78, 18.76) |  | 1.17 (0.80, 1.86) | 19.51 (13.34, 31.39) |  | 310.29 | 1.75 (1.59, 1.91) |
| Taiwan (Province of China) | 1.12 (0.96, 1.57) | 15.05 (13.19, 21.32) |  | 7.01 (4.73, 9.40) | 38.30 (25.95, 51.10) |  | 526.29 | 3.47 (3.14, 3.81) |
| Tajikistan | 0.10 (0.08, 0.12) | 8.76 (7.09, 10.55) |  | 0.19 (0.14, 0.27) | 11.94 (7.95, 17.30) |  | 94.65 | 1.29 (1.13, 1.45) |
| Thailand | 1.28 (0.76, 1.82) | 29.21 (17.16, 40.33) |  | 3.27 (2.05, 4.54) | 34.03 (21.58, 46.67) |  | 154.67 | 0.50 (0.45, 0.54) |
| Timor-Leste | 1.55 (1.08, 1.89) | 11.78 (8.58, 14.22) |  | 7.81 (5.35, 10.88) | 17.49 (12.10, 24.24) |  | 404.76 | 1.17 (1.04, 1.30) |
| Togo | 0.01 (0.01, 0.01) | 9.10 (5.90, 12.53) |  | 0.06 (0.04, 0.08) | 16.96 (11.33, 22.23) |  | 595.69 | 2.48 (2.30, 2.66) |
| Tokelau | 0.09 (0.07, 0.12) | 20.01 (15.88, 27.18) |  | 0.38 (0.21, 0.55) | 35.84 (20.44, 50.03) |  | 333.66 | 2.25 (2.09, 2.40) |
| Tonga | 0.00 (0.00, 0.00) | 23.00 (16.43, 32.88) |  | 0.00 (0.00, 0.00) | 31.81 (24.61, 40.09) |  | 35.06 | 1.19 (1.15, 1.24) |
| Trinidad and Tobago | 0.01 (0.01, 0.01) | 34.85 (27.57, 42.79) |  | 0.01 (0.01, 0.02) | 43.63 (32.83, 53.43) |  | 88.96 | 0.65 (0.44, 0.85) |
| Tunisia | 0.30 (0.21, 0.34) | 81.95 (59.92, 93.19) |  | 0.83 (0.55, 1.15) | 97.94 (65.33, 134.16) |  | 179.61 | 0.24 (0.07, 0.41) |
| Turkey | 0.27 (0.20, 0.35) | 11.64 (8.62, 15.34) |  | 1.27 (0.88, 1.80) | 21.29 (14.91, 30.45) |  | 374.7 | 2.11 (2.04, 2.18) |
| Turkmenistan | 2.87 (2.00, 3.66) | 19.64 (13.71, 24.93) |  | 14.00 (9.79, 18.45) | 35.06 (24.63, 46.36) |  | 388.6 | 2.45 (1.99, 2.92) |
| Tuvalu | 0.06 (0.05, 0.10) | 9.44 (7.78, 16.30) |  | 0.18 (0.13, 0.32) | 12.23 (8.89, 22.27) |  | 196.1 | 1.06 (0.92, 1.20) |
| Uganda | 0.00 (0.00, 0.00) | 29.46 (22.25, 41.32) |  | 0.00 (0.00, 0.00) | 35.57 (27.12, 46.60) |  | 116.78 | 0.50 (0.42, 0.58) |
| Ukraine | 1.05 (0.81, 1.47) | 40.59 (31.75, 56.87) |  | 2.98 (2.36, 3.70) | 62.50 (49.94, 76.16) |  | 184.72 | 1.45 (1.26, 1.64) |
| United Arab Emirates | 5.74 (4.96, 8.41) | 24.41 (21.15, 37.50) |  | 9.75 (7.49, 14.49) | 34.32 (26.43, 51.96) |  | 69.86 | 1.07 (0.87, 1.27) |
| United Kingdom | 0.03 (0.02, 0.05) | 17.01 (10.26, 29.45) |  | 0.62 (0.32, 1.20) | 24.20 (13.69, 46.92) |  | 1824.7 | 0.75 (0.34, 1.15) |
| Tanzania | 22.15 (16.58, 26.63) | 58.31 (44.20, 70.91) |  | 51.12 (38.00, 70.02) | 86.04 (63.95, 118.02) |  | 130.75 | 1.25 (1.06, 1.44) |
| United States of America | 181.82 (124.71, 201.31) | 131.93 (91.12, 146.23) |  | 308.58 (240.78, 460.98) | 118.25 (92.22, 175.43) |  | 69.72 | -0.84 (-1.00, -0.69) |
| United States Virgin Islands | 0.04 (0.03, 0.06) | 119.09 (90.51, 167.51) |  | 0.17 (0.14, 0.22) | 202.78 (163.47, 254.64) |  | 338.19 | 2.45 (2.10, 2.80) |
| Uruguay | 0.78 (0.66, 1.07) | 47.17 (40.05, 64.39) |  | 1.55 (1.13, 2.07) | 66.90 (48.89, 89.58) |  | 97.1 | 1.12 (0.70, 1.54) |
| Uzbekistan | 0.30 (0.21, 0.36) | 7.64 (5.32, 9.25) |  | 0.74 (0.57, 1.05) | 13.22 (8.57, 16.26) |  | 145.76 | 2.14 (1.88, 2.39) |
| Vanuatu | 0.01 (0.00, 0.01) | 22.87 (15.49, 33.40) |  | 0.02 (0.01, 0.03) | 28.78 (21.24, 37.80) |  | 231.22 | 0.79 (0.74, 0.85) |
| Venezuela | 1.78 (1.54, 2.58) | 44.07 (38.25, 64.67) |  | 13.71 (8.88, 19.09) | 104.68 (68.87, 143.93) |  | 668.01 | 2.67 (2.04, 3.31) |
| Viet Nam | 1.29 (0.98, 1.76) | 9.27 (7.04, 12.79) |  | 5.81 (4.35, 8.49) | 18.20 (13.98, 26.51) |  | 349.58 | 2.46 (2.41, 2.51) |
| Yemen | 0.17 (0.11, 0.24) | 10.27 (6.47, 14.68) |  | 0.79 (0.57, 1.07) | 14.74 (10.84, 19.58) |  | 366.63 | 1.62 (1.49, 1.75) |
| Zambia | 0.35 (0.20, 0.46) | 28.89 (16.53, 37.70) |  | 0.87 (0.51, 1.20) | 35.80 (20.95, 49.21) |  | 151.91 | 0.75 (0.68, 0.83) |
| Zimbabwe | 0.75 (0.60, 1.02) | 50.94 (41.66, 68.20) |  | 1.43 (1.06, 1.75) | 65.30 (49.12, 79.57) |  | 91.28 | 0.84 (0.57, 1.11) |

ASIR: age-standardized incidence rate; CI: confidence interval; EAPC: estimated annual percentage change; UI: uncertainty interval.

**Table S3**. The deaths and ASMRs of prostate cancer in 1990 and 2019 and their temporal trends from 1990 to 2019 at the national level.

| Countries | 1990 | |  | 2019 | |  | 1990-2019 | |
| --- | --- | --- | --- | --- | --- | --- | --- | --- |
|  | Death  No. x 10^3^ (95% UI) | ASMR per 100, 000  No. (95% UI) |  | Death  No. x 10^3^ (95% UI) | ASMR per 100, 000  No. (95% UI) |  | Percentage change  of deaths (%) | EAPC  No. (95% CI) |
| Afghanistan | 0.35 (0.26, 0.49) | 12.20 (8.63, 16.88) |  | 0.51 (0.38, 0.66) | 13.06 (9.68, 17.10) |  | 43.76 | 0.26 (0.21, 0.32) |
| Albania | 0.14 (0.11, 0.24) | 21.06 (15.62, 36.63) |  | 0.39 (0.24, 0.67) | 20.42 (12.93, 35.23) |  | 172.27 | -0.10 (-0.23, 0.02) |
| Algeria | 0.47 (0.35, 0.61) | 11.29 (8.76, 14.55) |  | 1.28 (0.96, 1.74) | 9.74 (7.40, 13.34) |  | 173.46 | -0.67 (-0.71, -0.62) |
| American Samoa | 0.00 (0.00, 0.00) | 47.10 (38.99, 60.67) |  | 0.01 (0.01, 0.01) | 47.25 (38.12, 55.20) |  | 144.16 | 0.24 (0.11, 0.36) |
| Andorra | 0.00 (0.00, 0.01) | 24.99 (19.16, 32.95) |  | 0.01 (0.01, 0.02) | 21.45 (16.11, 29.58) |  | 196.41 | -0.58 (-0.63, -0.53) |
| Angola | 0.29 (0.18, 0.38) | 26.15 (16.15, 35.14) |  | 0.91 (0.63, 1.17) | 32.59 (22.32, 42.30) |  | 217.81 | 0.75 (0.71, 0.79) |
| Antigua and Barbuda | 0.02 (0.01, 0.02) | 78.60 (65.83, 105.14) |  | 0.03 (0.02, 0.04) | 83.76 (61.50, 103.14) |  | 84.47 | 0.25 (-0.11, 0.61) |
| Argentina | 3.51 (3.11, 5.04) | 29.08 (25.54, 42.08) |  | 6.37 (5.38, 8.97) | 29.02 (24.28, 41.15) |  | 81.6 | -0.22 (-0.47, 0.04) |
| Armenia | 0.10 (0.08, 0.13) | 11.05 (9.38, 15.33) |  | 0.28 (0.16, 0.35) | 17.77 (10.22, 21.69) |  | 189.86 | 1.75 (1.62, 1.87) |
| Australia | 2.21 (1.70, 2.70) | 29.89 (22.78, 36.32) |  | 4.54 (3.71, 6.43) | 22.21 (18.10, 31.35) |  | 105.02 | -1.48 (-1.64, -1.32) |
| Austria | 1.15 (0.90, 1.42) | 28.00 (21.78, 34.45) |  | 1.69 (1.41, 2.67) | 20.42 (16.94, 31.83) |  | 46.61 | -1.41 (-1.58, -1.24) |
| Azerbaijan | 0.19 (0.16, 0.25) | 12.31 (10.28, 16.62) |  | 0.41 (0.32, 0.55) | 14.78 (11.33, 20.51) |  | 116.05 | 0.92 (0.80, 1.04) |
| Bahamas | 0.04 (0.03, 0.05) | 72.02 (60.44, 101.74) |  | 0.10 (0.08, 0.14) | 74.09 (58.93, 103.14) |  | 180.16 | -0.01 (-0.21, 0.19) |
| Bahrain | 0.01 (0.01, 0.01) | 18.23 (13.52, 22.50) |  | 0.04 (0.03, 0.05) | 16.12 (11.75, 20.78) |  | 323.51 | -0.46 (-0.74, -0.18) |
| Bangladesh | 1.70 (1.19, 2.26) | 8.48 (5.84, 11.27) |  | 4.43 (2.58, 6.73) | 7.75 (4.56, 11.70) |  | 160.4 | -0.44 (-0.62, -0.25) |
| Barbados | 0.08 (0.07, 0.11) | 67.15 (55.23, 86.57) |  | 0.17 (0.13, 0.21) | 81.75 (60.35, 99.86) |  | 108.11 | 0.01 (-0.33, 0.36) |
| Belarus | 0.61 (0.52, 1.08) | 15.41 (12.94, 27.77) |  | 1.04 (0.78, 1.62) | 19.79 (14.85, 31.19) |  | 70.71 | 0.62 (0.43, 0.81) |
| Belgium | 2.11 (1.52, 2.39) | 38.36 (26.82, 42.56) |  | 2.53 (2.08, 4.15) | 23.19 (19.15, 37.78) |  | 19.97 | -2.16 (-2.28, -2.04) |
| Belize | 0.01 (0.01, 0.02) | 28.26 (22.17, 47.77) |  | 0.05 (0.04, 0.06) | 39.90 (31.87, 52.52) |  | 291.25 | 0.81 (0.22, 1.41) |
| Benin | 0.17 (0.14, 0.23) | 20.61 (16.71, 27.91) |  | 0.50 (0.31, 0.67) | 32.39 (19.47, 42.16) |  | 200.45 | 1.86 (1.67, 2.04) |
| Bermuda | 0.01 (0.01, 0.01) | 52.64 (36.11, 62.35) |  | 0.03 (0.02, 0.03) | 49.04 (36.67, 61.03) |  | 144.23 | -0.26 (-0.37, -0.16) |
| Bhutan | 0.01 (0.00, 0.01) | 7.78 (4.58, 11.55) |  | 0.02 (0.01, 0.04) | 10.16 (5.79, 15.97) |  | 351.88 | 1.04 (0.98, 1.10) |
| Bolivia | 0.34 (0.27, 0.42) | 30.89 (24.91, 38.10) |  | 1.30 (0.96, 1.66) | 39.25 (28.77, 49.96) |  | 285.93 | 0.80 (0.74, 0.86) |
| Bosnia and Herzegovina | 0.17 (0.15, 0.22) | 14.24 (12.31, 18.38) |  | 0.46 (0.31, 0.61) | 19.53 (13.22, 25.30) |  | 167.84 | 1.30 (1.03, 1.57) |
| Botswana | 0.08 (0.06, 0.10) | 43.96 (33.29, 55.15) |  | 0.19 (0.13, 0.25) | 53.73 (37.20, 68.46) |  | 156.26 | 0.22 (-0.22, 0.66) |
| Brazil | 8.18 (7.20, 12.35) | 26.93 (23.20, 40.66) |  | 23.34 (19.86, 34.32) | 25.48 (21.45, 37.25) |  | 185.41 | -0.28 (-0.52, -0.04) |
| Brunei | 0.00 (0.00, 0.01) | 19.87 (15.18, 25.43) |  | 0.01 (0.01, 0.02) | 26.50 (16.26, 34.40) |  | 216.34 | 1.93 (1.46, 2.40) |
| Bulgaria | 0.74 (0.64, 1.21) | 14.98 (12.89, 24.75) |  | 1.33 (0.96, 1.69) | 21.79 (15.87, 27.79) |  | 80.77 | 2.07 (1.77, 2.37) |
| Burkina Faso | 0.29 (0.23, 0.40) | 19.42 (15.30, 26.46) |  | 0.87 (0.54, 1.14) | 30.59 (18.87, 39.82) |  | 195.04 | 1.93 (1.82, 2.05) |
| Burundi | 0.23 (0.12, 0.32) | 27.18 (14.40, 36.36) |  | 0.42 (0.26, 0.60) | 26.15 (16.35, 36.46) |  | 80.16 | -0.19 (-0.25, -0.14) |
| Cambodia | 0.19 (0.14, 0.24) | 14.34 (10.71, 17.85) |  | 0.66 (0.47, 0.82) | 19.62 (14.12, 24.27) |  | 250.65 | 1.18 (1.11, 1.25) |
| Cameroon | 0.34 (0.26, 0.47) | 22.28 (17.33, 30.70) |  | 1.38 (0.76, 2.05) | 36.54 (20.11, 54.23) |  | 306.26 | 1.90 (1.74, 2.07) |
| Canada | 4.09 (2.78, 4.48) | 32.94 (22.17, 36.17) |  | 6.52 (5.42, 9.20) | 20.21 (16.78, 28.36) |  | 59.51 | -2.30 (-2.53, -2.07) |
| Cape Verde | 0.02 (0.02, 0.03) | 21.46 (16.64, 32.05) |  | 0.11 (0.07, 0.13) | 70.28 (45.76, 87.12) |  | 383.58 | 2.53 (1.71, 3.37) |
| Central African Republic | 0.09 (0.06, 0.12) | 29.24 (19.58, 39.86) |  | 0.15 (0.10, 0.20) | 29.44 (20.00, 39.95) |  | 61.12 | 0.01 (-0.03, 0.05) |
| Chad | 0.19 (0.14, 0.27) | 16.65 (12.22, 23.61) |  | 0.64 (0.40, 0.86) | 29.01 (18.22, 38.55) |  | 235.12 | 2.27 (2.06, 2.49) |
| Chile | 0.92 (0.76, 1.22) | 25.22 (21.09, 33.24) |  | 2.89 (2.11, 3.37) | 29.04 (21.02, 33.94) |  | 214.52 | 0.31 (0.04, 0.59) |
| China | 20.38 (15.82, 24.68) | 8.22 (6.62, 10.28) |  | 54.39 (42.90, 71.31) | 7.79 (6.22, 9.94) |  | 166.85 | -0.22 (-0.27, -0.17) |
| Colombia | 1.52 (1.24, 2.00) | 22.77 (18.68, 30.30) |  | 4.46 (3.23, 6.99) | 18.75 (13.62, 29.32) |  | 193.89 | -1.20 (-1.45, -0.95) |
| Comoros | 0.02 (0.01, 0.03) | 27.87 (15.60, 38.25) |  | 0.05 (0.03, 0.07) | 29.34 (19.23, 38.93) |  | 114.58 | 0.10 (0.02, 0.17) |
| Congo | 0.10 (0.07, 0.12) | 33.65 (24.25, 41.69) |  | 0.25 (0.17, 0.32) | 34.38 (23.69, 42.91) |  | 149.83 | 0.01 (-0.14, 0.16) |
| Cook Islands | 0.00 (0.00, 0.00) | 70.90 (59.49, 85.56) |  | 0.01 (0.01, 0.01) | 56.76 (46.44, 68.82) |  | 86.48 | -0.84 (-0.90, -0.77) |
| Costa Rica | 0.15 (0.12, 0.19) | 19.85 (15.84, 25.78) |  | 0.57 (0.34, 0.76) | 25.71 (15.54, 34.08) |  | 288.1 | 0.63 (0.28, 0.97) |
| Cote d'Ivoire | 0.27 (0.22, 0.37) | 21.71 (17.37, 29.23) |  | 1.10 (0.63, 1.57) | 32.65 (18.84, 45.94) |  | 300.79 | 1.67 (1.51, 1.84) |
| Croatia | 0.51 (0.39, 0.63) | 25.48 (19.26, 31.28) |  | 0.91 (0.63, 1.16) | 25.08 (17.23, 31.55) |  | 79.62 | 0.30 (0.12, 0.48) |
| Cuba | 1.48 (1.23, 1.94) | 30.90 (25.81, 40.70) |  | 3.40 (2.00, 4.30) | 37.34 (21.91, 47.32) |  | 128.94 | 0.62 (0.55, 0.70) |
| Cyprus | 0.07 (0.06, 0.09) | 21.53 (17.49, 29.18) |  | 0.19 (0.15, 0.23) | 23.18 (17.58, 27.88) |  | 169.49 | 0.25 (-0.15, 0.66) |
| Czech Republic | 1.10 (0.88, 1.45) | 22.44 (18.10, 30.00) |  | 1.89 (1.39, 2.41) | 21.25 (15.64, 26.91) |  | 72.08 | -0.30 (-0.59, -0.01) |
| Democratic Republic of the Congo | 1.21 (0.84, 1.64) | 26.68 (18.17, 36.48) |  | 2.57 (1.75, 3.45) | 28.30 (18.90, 38.34) |  | 113 | 0.14 (0.10, 0.18) |
| Denmark | 1.24 (0.99, 1.56) | 36.44 (29.18, 46.22) |  | 1.71 (1.36, 2.24) | 31.35 (25.16, 41.30) |  | 37.33 | -0.46 (-0.66, -0.26) |
| Djibouti | 0.01 (0.01, 0.02) | 28.35 (16.48, 39.04) |  | 0.07 (0.04, 0.09) | 32.68 (20.89, 44.07) |  | 464.21 | 0.46 (0.42, 0.51) |
| Dominica | 0.03 (0.02, 0.04) | 100.43 (86.17, 129.49) |  | 0.05 (0.04, 0.06) | 126.28 (93.14, 156.53) |  | 75.42 | 0.70 (0.51, 0.89) |
| Dominican Republic | 0.46 (0.31, 0.81) | 31.76 (21.47, 55.99) |  | 1.69 (1.15, 2.76) | 43.56 (29.98, 70.93) |  | 267.93 | 1.42 (0.83, 2.02) |
| Ecuador | 0.52 (0.45, 0.66) | 24.07 (20.70, 30.91) |  | 1.68 (1.24, 2.21) | 28.08 (20.22, 36.67) |  | 223.91 | 0.81 (0.58, 1.04) |
| Egypt | 0.66 (0.56, 0.85) | 6.46 (5.41, 8.73) |  | 1.81 (1.29, 2.51) | 7.01 (5.06, 9.66) |  | 174.39 | 0.23 (0.06, 0.39) |
| El Salvador | 0.17 (0.14, 0.24) | 14.15 (11.60, 20.22) |  | 0.56 (0.40, 0.72) | 21.10 (15.31, 27.32) |  | 225.66 | 0.92 (0.47, 1.36) |
| Equatorial Guinea | 0.01 (0.01, 0.02) | 25.98 (15.56, 36.66) |  | 0.05 (0.03, 0.06) | 36.68 (24.41, 47.63) |  | 221.25 | 1.29 (1.21, 1.37) |
| Eritrea | 0.06 (0.03, 0.09) | 25.36 (10.01, 36.30) |  | 0.19 (0.11, 0.26) | 28.71 (15.81, 38.85) |  | 210.74 | 0.12 (-0.13, 0.37) |
| Estonia | 0.13 (0.09, 0.15) | 20.78 (15.17, 24.10) |  | 0.28 (0.14, 0.36) | 29.00 (14.31, 37.63) |  | 120.42 | 1.47 (1.21, 1.72) |
| Eswatini | 0.03 (0.03, 0.04) | 42.54 (33.71, 52.07) |  | 0.07 (0.05, 0.10) | 52.83 (37.54, 66.42) |  | 117.75 | 0.87 (0.60, 1.14) |
| Ethiopia | 0.86 (0.60, 1.24) | 11.40 (7.69, 15.81) |  | 2.29 (1.21, 3.74) | 13.83 (7.51, 22.04) |  | 166.38 | 0.73 (0.63, 0.83) |
| Federated States of Micronesia | 0.00 (0.00, 0.01) | 28.90 (21.31, 42.09) |  | 0.01 (0.00, 0.01) | 33.44 (25.07, 41.80) |  | 40.15 | 0.55 (0.46, 0.64) |
| Fiji | 0.02 (0.01, 0.03) | 22.79 (12.55, 32.15) |  | 0.05 (0.02, 0.07) | 25.89 (12.19, 36.17) |  | 117.15 | 0.70 (0.52, 0.88) |
| Finland | 0.68 (0.52, 0.82) | 28.93 (22.34, 35.20) |  | 1.29 (1.06, 1.83) | 22.60 (18.43, 32.05) |  | 91 | -1.32 (-1.50, -1.15) |
| France | 11.46 (7.77, 12.71) | 36.60 (24.80, 40.38) |  | 14.50 (11.66, 22.03) | 21.72 (17.55, 33.33) |  | 26.56 | -2.17 (-2.35, -1.99) |
| Gabon | 0.05 (0.04, 0.07) | 32.57 (23.03, 40.38) |  | 0.12 (0.08, 0.16) | 39.45 (25.69, 50.77) |  | 115.71 | 0.57 (0.53, 0.62) |
| Gambia | 0.01 (0.01, 0.01) | 8.86 (7.07, 11.94) |  | 0.04 (0.03, 0.05) | 11.22 (8.33, 15.15) |  | 262.42 | 0.73 (0.63, 0.82) |
| Georgia | 0.26 (0.22, 0.36) | 13.60 (11.54, 19.76) |  | 0.46 (0.29, 0.56) | 19.57 (12.35, 23.84) |  | 76.21 | 2.53 (1.92, 3.14) |
| Germany | 11.64 (8.90, 14.16) | 27.09 (20.81, 33.17) |  | 20.15 (15.71, 25.91) | 21.89 (16.74, 27.79) |  | 73.06 | -1.12 (-1.29, -0.95) |
| Ghana | 0.90 (0.64, 1.17) | 44.89 (32.82, 57.20) |  | 2.13 (1.72, 2.75) | 44.17 (35.72, 57.50) |  | 137.85 | -0.36 (-0.55, -0.17) |
| Greece | 1.49 (1.25, 1.98) | 23.26 (19.24, 30.60) |  | 2.68 (2.20, 3.71) | 19.89 (16.57, 27.98) |  | 80.16 | -1.09 (-1.39, -0.78) |
| Greenland | 0.00 (0.00, 0.00) | 14.69 (11.48, 22.15) |  | 0.00 (0.00, 0.00) | 15.00 (11.94, 18.93) |  | 175.33 | 0.03 (-0.06, 0.13) |
| Grenada | 0.02 (0.02, 0.03) | 74.98 (64.07, 107.73) |  | 0.04 (0.02, 0.04) | 98.79 (64.85, 114.11) |  | 54.74 | 0.89 (0.02, 1.77) |
| Guam | 0.00 (0.00, 0.01) | 23.29 (19.05, 29.40) |  | 0.01 (0.01, 0.02) | 17.45 (13.80, 21.82) |  | 189.16 | -0.78 (-1.15, -0.40) |
| Guatemala | 0.22 (0.15, 0.49) | 18.04 (12.86, 40.17) |  | 1.25 (0.94, 1.68) | 32.49 (24.01, 40.95) |  | 476.34 | 1.66 (0.93, 2.39) |
| Guinea | 0.40 (0.23, 0.52) | 29.74 (17.34, 39.06) |  | 0.80 (0.42, 1.20) | 36.79 (19.59, 55.00) |  | 99.98 | 0.97 (0.81, 1.13) |
| Guinea-Bissau | 0.03 (0.02, 0.05) | 22.19 (16.78, 30.43) |  | 0.07 (0.04, 0.11) | 34.78 (19.47, 50.16) |  | 122.66 | 1.91 (1.75, 2.06) |
| Guyana | 0.08 (0.06, 0.11) | 56.81 (46.49, 83.75) |  | 0.13 (0.10, 0.18) | 63.02 (48.44, 86.32) |  | 68.5 | 0.21 (0.04, 0.37) |
| Haiti | 0.57 (0.40, 0.75) | 56.63 (39.17, 74.96) |  | 1.38 (0.96, 1.88) | 60.04 (42.04, 81.78) |  | 140.77 | 0.20 (0.18, 0.22) |
| Honduras | 0.13 (0.09, 0.19) | 16.76 (11.56, 23.86) |  | 0.52 (0.38, 0.77) | 22.87 (16.41, 33.87) |  | 291.35 | 1.65 (1.31, 2.00) |
| Hungary | 1.27 (1.01, 1.62) | 24.59 (19.10, 30.79) |  | 1.57 (1.20, 2.05) | 20.99 (16.11, 27.47) |  | 23.46 | -0.90 (-1.09, -0.70) |
| Iceland | 0.04 (0.03, 0.05) | 30.76 (23.80, 37.09) |  | 0.06 (0.05, 0.08) | 23.36 (18.14, 29.57) |  | 62 | -1.07 (-1.31, -0.84) |
| India | 10.87 (7.94, 12.81) | 8.03 (5.85, 9.45) |  | 32.11 (25.98, 42.13) | 7.75 (6.30, 10.23) |  | 195.45 | -0.29 (-0.40, -0.18) |
| Indonesia | 3.19 (2.48, 3.87) | 10.32 (8.16, 12.59) |  | 11.41 (8.00, 14.26) | 17.48 (12.39, 21.85) |  | 257.32 | 1.92 (1.82, 2.01) |
| Iran | 0.92 (0.73, 1.15) | 11.43 (8.97, 14.38) |  | 3.92 (2.91, 4.44) | 12.83 (9.58, 14.55) |  | 326.87 | 0.51 (0.30, 0.73) |
| Iraq | 0.27 (0.19, 0.38) | 9.02 (6.43, 12.81) |  | 0.86 (0.65, 1.16) | 11.61 (8.81, 15.87) |  | 222.03 | 0.63 (0.48, 0.77) |
| Ireland | 0.50 (0.38, 0.61) | 30.46 (23.31, 37.79) |  | 0.80 (0.64, 1.07) | 23.55 (19.03, 31.44) |  | 58.4 | -1.27 (-1.51, -1.04) |
| Israel | 0.42 (0.36, 0.57) | 20.09 (16.61, 27.09) |  | 0.76 (0.62, 1.28) | 14.20 (11.46, 23.78) |  | 80.42 | -1.74 (-2.01, -1.48) |
| Italy | 7.08 (6.08, 9.56) | 20.36 (17.54, 27.83) |  | 11.67 (9.58, 16.86) | 16.43 (13.54, 23.70) |  | 64.81 | -1.02 (-1.22, -0.81) |
| Jamaica | 0.28 (0.23, 0.37) | 34.78 (28.87, 45.79) |  | 0.85 (0.49, 1.08) | 61.27 (35.12, 77.87) |  | 199.31 | 1.90 (1.31, 2.49) |
| Japan | 5.84 (4.91, 7.94) | 9.80 (8.20, 13.14) |  | 16.41 (12.23, 20.57) | 8.60 (6.50, 10.88) |  | 180.82 | -0.50 (-0.59, -0.41) |
| Jordan | 0.05 (0.04, 0.06) | 11.05 (8.72, 13.97) |  | 0.26 (0.18, 0.33) | 11.49 (8.14, 14.88) |  | 452.44 | 0.31 (0.05, 0.58) |
| Kazakhstan | 0.39 (0.29, 0.50) | 11.15 (8.32, 14.58) |  | 0.60 (0.47, 0.81) | 11.96 (9.46, 16.50) |  | 53.78 | 0.67 (0.52, 0.82) |
| Kenya | 0.53 (0.28, 0.93) | 17.13 (9.13, 29.39) |  | 1.89 (1.25, 2.70) | 27.46 (18.89, 37.64) |  | 254.85 | 1.98 (1.76, 2.20) |
| Kiribati | 0.00 (0.00, 0.00) | 18.46 (13.35, 26.19) |  | 0.00 (0.00, 0.00) | 16.74 (12.56, 23.26) |  | 39.55 | -0.40 (-0.58, -0.22) |
| Kuwait | 0.02 (0.01, 0.02) | 7.61 (5.80, 9.93) |  | 0.11 (0.08, 0.14) | 10.23 (7.45, 13.48) |  | 551.7 | 0.94 (0.58, 1.30) |
| Kyrgyzstan | 0.09 (0.05, 0.11) | 9.67 (4.87, 11.30) |  | 0.11 (0.08, 0.14) | 8.16 (5.62, 9.97) |  | 19.6 | -0.83 (-1.05, -0.61) |
| Lao | 0.09 (0.06, 0.11) | 12.81 (9.07, 16.31) |  | 0.21 (0.15, 0.26) | 14.93 (10.84, 18.17) |  | 144.2 | 0.41 (0.36, 0.45) |
| Latvia | 0.22 (0.17, 0.27) | 19.78 (15.26, 24.82) |  | 0.40 (0.23, 0.51) | 27.97 (15.65, 35.96) |  | 83.05 | 1.82 (1.43, 2.20) |
| Lebanon | 0.18 (0.14, 0.23) | 22.82 (16.74, 28.88) |  | 0.59 (0.36, 0.86) | 25.66 (15.98, 37.75) |  | 222.16 | 0.70 (0.57, 0.84) |
| Lesotho | 0.11 (0.08, 0.14) | 38.57 (28.64, 49.30) |  | 0.19 (0.14, 0.23) | 56.31 (41.92, 69.06) |  | 68.96 | 1.31 (1.07, 1.56) |
| Liberia | 0.10 (0.08, 0.14) | 20.12 (15.71, 28.14) |  | 0.22 (0.13, 0.30) | 30.24 (18.52, 40.98) |  | 122.35 | 1.80 (1.61, 2.00) |
| Libya | 0.07 (0.05, 0.10) | 9.55 (6.18, 13.08) |  | 0.21 (0.16, 0.27) | 10.19 (7.62, 13.27) |  | 185.12 | 0.29 (0.21, 0.37) |
| Lithuania | 0.27 (0.22, 0.36) | 17.48 (13.77, 22.75) |  | 0.55 (0.29, 0.69) | 26.11 (13.83, 32.76) |  | 101.55 | 1.79 (1.26, 2.33) |
| Luxembourg | 0.06 (0.04, 0.07) | 32.85 (22.22, 37.37) |  | 0.08 (0.07, 0.13) | 19.09 (14.96, 30.02) |  | 35.73 | -2.11 (-2.21, -2.01) |
| Macedonia | 0.11 (0.08, 0.19) | 15.70 (10.87, 27.44) |  | 0.29 (0.22, 0.38) | 22.68 (17.68, 30.55) |  | 152.78 | 1.53 (1.31, 1.74) |
| Madagascar | 0.49 (0.28, 0.66) | 23.93 (13.67, 32.00) |  | 0.78 (0.47, 1.11) | 22.75 (13.43, 31.76) |  | 57.45 | -0.38 (-0.52, -0.24) |
| Malawi | 0.20 (0.14, 0.31) | 15.06 (10.95, 23.16) |  | 0.43 (0.34, 0.61) | 18.47 (14.59, 26.54) |  | 117.96 | 0.86 (0.75, 0.98) |
| Malaysia | 0.34 (0.26, 0.41) | 9.72 (7.43, 11.96) |  | 1.15 (0.80, 1.52) | 11.17 (7.74, 14.69) |  | 243.77 | 0.02 (-0.23, 0.26) |
| Maldives | 0.00 (0.00, 0.00) | 7.83 (5.75, 11.58) |  | 0.01 (0.01, 0.01) | 8.31 (6.35, 11.21) |  | 303.54 | 0.06 (-0.04, 0.17) |
| Mali | 0.16 (0.13, 0.20) | 11.15 (9.00, 14.22) |  | 0.45 (0.35, 0.58) | 13.53 (10.64, 17.27) |  | 174.95 | 0.70 (0.66, 0.75) |
| Malta | 0.03 (0.02, 0.04) | 19.61 (15.37, 25.26) |  | 0.06 (0.05, 0.09) | 13.39 (10.80, 20.18) |  | 80.84 | -1.41 (-1.55, -1.26) |
| Marshall Islands | 0.00 (0.00, 0.00) | 32.96 (22.48, 47.79) |  | 0.00 (0.00, 0.00) | 30.64 (23.01, 39.48) |  | 75.78 | 0.01 (-0.21, 0.23) |
| Mauritania | 0.08 (0.06, 0.11) | 21.19 (16.06, 29.36) |  | 0.26 (0.16, 0.33) | 30.23 (18.92, 39.36) |  | 229.54 | 1.55 (1.39, 1.70) |
| Mauritius | 0.03 (0.02, 0.04) | 12.50 (10.54, 18.41) |  | 0.10 (0.07, 0.12) | 15.36 (10.53, 19.44) |  | 240.56 | 0.27 (-0.07, 0.61) |
| Mexico | 3.17 (2.59, 4.26) | 19.18 (15.93, 25.96) |  | 9.26 (7.08, 12.68) | 19.37 (14.77, 26.70) |  | 191.97 | -0.17 (-0.29, -0.06) |
| Moldova | 0.17 (0.15, 0.24) | 11.37 (9.92, 16.82) |  | 0.31 (0.23, 0.38) | 13.89 (10.37, 17.62) |  | 84.88 | 1.39 (0.96, 1.83) |
| Monaco | 0.01 (0.01, 0.01) | 26.43 (20.58, 35.17) |  | 0.01 (0.01, 0.02) | 23.83 (19.05, 32.55) |  | 43.56 | -0.29 (-0.36, -0.22) |
| Mongolia | 0.03 (0.02, 0.04) | 8.15 (6.47, 11.00) |  | 0.05 (0.04, 0.07) | 8.74 (6.93, 11.19) |  | 88.48 | 0.00 (-0.16, 0.17) |
| Montenegro | 0.05 (0.04, 0.06) | 22.03 (18.30, 28.12) |  | 0.10 (0.08, 0.13) | 26.69 (21.06, 32.88) |  | 108.81 | 0.81 (0.72, 0.89) |
| Morocco | 0.39 (0.28, 0.51) | 7.91 (5.56, 10.68) |  | 1.18 (0.87, 1.56) | 10.27 (7.44, 13.75) |  | 204.87 | 0.60 (0.14, 1.07) |
| Mozambique | 0.19 (0.14, 0.29) | 9.70 (7.48, 14.07) |  | 0.40 (0.28, 0.66) | 13.06 (9.50, 19.90) |  | 117.68 | 1.17 (1.10, 1.23) |
| Myanmar | 0.90 (0.67, 1.12) | 12.30 (9.34, 15.17) |  | 2.18 (1.59, 2.72) | 15.14 (11.13, 18.71) |  | 141.28 | 0.76 (0.73, 0.79) |
| Namibia | 0.07 (0.05, 0.10) | 26.01 (18.02, 39.94) |  | 0.19 (0.13, 0.24) | 42.49 (30.53, 52.21) |  | 184.01 | 2.08 (1.95, 2.22) |
| Nauru | 0.00 (0.00, 0.00) | 29.48 (22.87, 39.20) |  | 0.00 (0.00, 0.00) | 34.41 (24.14, 46.44) |  | -9.47 | 0.58 (0.43, 0.72) |
| Nepal | 0.22 (0.13, 0.31) | 7.04 (4.15, 9.90) |  | 0.74 (0.44, 1.11) | 9.27 (5.61, 13.66) |  | 241.57 | 0.97 (0.82, 1.12) |
| Netherlands | 2.29 (1.72, 2.82) | 30.88 (22.97, 37.62) |  | 4.11 (3.14, 4.96) | 26.32 (20.00, 31.52) |  | 79.34 | -0.99 (-1.14, -0.83) |
| New Zealand | 0.49 (0.37, 0.58) | 31.64 (24.37, 38.34) |  | 0.87 (0.72, 1.23) | 22.93 (19.11, 32.46) |  | 79.13 | -1.61 (-1.79, -1.43) |
| Nicaragua | 0.13 (0.11, 0.18) | 26.96 (21.86, 37.18) |  | 0.48 (0.38, 0.63) | 32.99 (26.16, 43.23) |  | 261.07 | 0.58 (0.35, 0.80) |
| Niger | 0.15 (0.12, 0.21) | 16.83 (12.82, 23.02) |  | 0.69 (0.43, 0.98) | 27.73 (17.25, 38.54) |  | 347.72 | 2.13 (1.95, 2.30) |
| Nigeria | 6.00 (2.78, 8.97) | 39.86 (19.34, 57.68) |  | 15.37 (7.13, 23.07) | 51.66 (24.33, 76.20) |  | 156.31 | 1.17 (1.04, 1.30) |
| Niue | 0.00 (0.00, 0.00) | 28.34 (22.95, 37.86) |  | 0.00 (0.00, 0.00) | 32.52 (24.55, 39.58) |  | 13.41 | 0.62 (0.57, 0.67) |
| North Korea | 0.32 (0.23, 0.42) | 9.19 (6.29, 12.42) |  | 0.85 (0.65, 1.03) | 9.33 (7.15, 11.46) |  | 167.46 | 0.20 (0.09, 0.31) |
| Northern Mariana Islands | 0.00 (0.00, 0.00) | 22.04 (17.05, 32.61) |  | 0.00 (0.00, 0.01) | 28.00 (22.65, 33.18) |  | 329.9 | 1.20 (0.97, 1.43) |
| Norway | 1.07 (0.80, 1.27) | 36.39 (27.31, 43.57) |  | 1.37 (1.10, 1.77) | 30.02 (23.96, 38.80) |  | 28.2 | -0.90 (-1.08, -0.72) |
| Oman | 0.02 (0.01, 0.03) | 11.46 (7.70, 15.79) |  | 0.05 (0.04, 0.07) | 12.54 (9.98, 16.03) |  | 162.58 | 0.48 (0.35, 0.61) |
| Pakistan | 2.84 (2.05, 3.91) | 10.63 (7.66, 14.85) |  | 4.92 (3.61, 6.48) | 12.54 (9.50, 16.49) |  | 73.3 | 0.55 (0.41, 0.70) |
| Palau | 0.00 (0.00, 0.00) | 36.24 (28.00, 49.52) |  | 0.00 (0.00, 0.00) | 34.72 (27.55, 45.86) |  | 80.35 | -0.08 (-0.15, -0.01) |
| Palestine | 0.06 (0.04, 0.08) | 18.84 (12.74, 26.49) |  | 0.12 (0.08, 0.16) | 18.57 (11.56, 23.86) |  | 112.34 | -0.15 (-0.28, -0.01) |
| Panama | 0.13 (0.11, 0.19) | 19.94 (16.71, 28.42) |  | 0.49 (0.33, 0.64) | 25.39 (16.98, 33.36) |  | 268.85 | 0.47 (-0.00, 0.95) |
| Papua New Guinea | 0.10 (0.06, 0.14) | 17.62 (11.07, 26.35) |  | 0.33 (0.22, 0.45) | 23.32 (15.73, 31.48) |  | 234.93 | 1.08 (1.02, 1.15) |
| Paraguay | 0.15 (0.13, 0.20) | 17.66 (14.44, 23.46) |  | 0.56 (0.37, 0.76) | 25.44 (16.70, 34.22) |  | 267.82 | 1.18 (0.77, 1.60) |
| Peru | 1.07 (0.87, 1.47) | 21.98 (17.76, 30.25) |  | 3.29 (2.43, 4.41) | 21.82 (16.10, 29.35) |  | 206.01 | 0.21 (0.06, 0.37) |
| Philippines | 1.78 (1.37, 2.12) | 17.67 (13.72, 20.97) |  | 4.25 (2.86, 5.61) | 17.17 (11.70, 22.46) |  | 138.45 | -0.20 (-0.41, 0.01) |
| Poland | 2.86 (2.54, 4.17) | 19.06 (16.81, 28.68) |  | 6.54 (4.15, 8.26) | 24.00 (15.28, 30.13) |  | 128.65 | 0.67 (0.36, 0.98) |
| Portugal | 1.57 (1.21, 1.92) | 31.86 (23.86, 37.94) |  | 2.68 (2.23, 3.89) | 23.43 (19.55, 33.96) |  | 70.61 | -1.56 (-1.77, -1.35) |
| Puerto Rico | 0.51 (0.37, 0.61) | 33.37 (24.23, 38.91) |  | 0.89 (0.66, 1.25) | 25.10 (18.66, 35.63) |  | 72.98 | -1.41 (-1.55, -1.27) |
| Qatar | 0.00 (0.00, 0.00) | 15.11 (11.07, 19.87) |  | 0.03 (0.02, 0.05) | 19.19 (13.68, 26.05) |  | 797.11 | 1.02 (0.55, 1.49) |
| Romania | 1.31 (1.16, 1.97) | 12.46 (10.96, 19.39) |  | 2.63 (1.85, 3.21) | 16.54 (11.76, 20.23) |  | 100.13 | 0.88 (0.75, 1.01) |
| Russia | 5.45 (4.78, 8.49) | 11.69 (10.22, 18.60) |  | 12.93 (8.32, 15.93) | 16.64 (10.72, 20.39) |  | 137.33 | 1.48 (1.34, 1.61) |
| Rwanda | 0.29 (0.15, 0.40) | 29.23 (15.38, 39.97) |  | 0.54 (0.35, 0.73) | 31.74 (20.65, 42.46) |  | 88.26 | -0.13 (-0.34, 0.09) |
| Saint Kitts and Nevis | 0.01 (0.01, 0.02) | 82.05 (68.59, 108.69) |  | 0.02 (0.02, 0.02) | 97.13 (75.78, 116.04) |  | 66.21 | 0.64 (0.52, 0.77) |
| Saint Lucia | 0.02 (0.02, 0.03) | 81.06 (68.18, 106.49) |  | 0.07 (0.05, 0.09) | 80.21 (64.91, 104.89) |  | 179.78 | -0.64 (-0.89, -0.39) |
| Saint Vincent and the Grenadines | 0.02 (0.01, 0.03) | 67.37 (56.82, 99.44) |  | 0.05 (0.04, 0.06) | 82.60 (62.55, 99.98) |  | 174.67 | 0.41 (0.18, 0.63) |
| Samoa | 0.01 (0.00, 0.01) | 18.09 (14.41, 22.07) |  | 0.01 (0.01, 0.01) | 15.02 (11.84, 18.06) |  | 46.41 | -0.57 (-0.77, -0.37) |
| San Marino | 0.00 (0.00, 0.00) | 30.00 (23.48, 37.14) |  | 0.01 (0.01, 0.01) | 26.50 (17.93, 38.87) |  | 127.09 | -0.51 (-0.55, -0.46) |
| Sao Tome and Principe | 0.00 (0.00, 0.01) | 17.63 (14.06, 22.83) |  | 0.01 (0.01, 0.01) | 24.04 (18.05, 31.73) |  | 121.4 | 1.39 (1.22, 1.55) |
| Saudi Arabia | 0.23 (0.13, 0.36) | 10.42 (6.04, 16.99) |  | 0.48 (0.36, 0.72) | 8.95 (6.80, 13.44) |  | 112.7 | -1.04 (-1.29, -0.79) |
| Senegal | 0.28 (0.21, 0.38) | 21.93 (17.09, 30.48) |  | 0.97 (0.60, 1.28) | 35.88 (22.14, 46.96) |  | 253.01 | 2.09 (1.89, 2.28) |
| Serbia | 0.70 (0.56, 0.90) | 16.59 (13.25, 21.74) |  | 1.50 (1.07, 1.89) | 21.64 (15.89, 27.17) |  | 114.41 | 1.39 (1.12, 1.66) |
| Seychelles | 0.01 (0.01, 0.01) | 40.07 (28.90, 63.21) |  | 0.02 (0.02, 0.03) | 57.90 (42.10, 70.01) |  | 155.05 | 1.05 (0.70, 1.39) |
| Sierra Leone | 0.16 (0.13, 0.22) | 19.12 (14.85, 26.74) |  | 0.38 (0.24, 0.53) | 29.48 (18.36, 39.82) |  | 139.11 | 1.94 (1.76, 2.13) |
| Singapore | 0.07 (0.05, 0.09) | 9.84 (8.19, 13.47) |  | 0.25 (0.19, 0.32) | 8.43 (6.35, 10.60) |  | 275.52 | -0.63 (-0.82, -0.44) |
| Slovakia | 0.40 (0.35, 0.50) | 17.60 (15.37, 22.59) |  | 0.69 (0.46, 0.91) | 20.54 (13.61, 26.58) |  | 74.32 | 0.73 (0.56, 0.89) |
| Slovenia | 0.20 (0.14, 0.26) | 23.66 (17.35, 31.02) |  | 0.52 (0.33, 0.68) | 28.41 (17.67, 36.85) |  | 165.86 | 0.67 (0.37, 0.97) |
| Solomon Islands | 0.01 (0.01, 0.02) | 23.02 (15.27, 33.78) |  | 0.02 (0.02, 0.03) | 27.32 (21.04, 34.03) |  | 132.59 | 0.67 (0.62, 0.71) |
| Somalia | 0.20 (0.10, 0.29) | 25.83 (13.51, 37.24) |  | 0.45 (0.26, 0.67) | 25.08 (14.97, 37.14) |  | 128.58 | -0.07 (-0.13, -0.02) |
| South Africa | 2.34 (1.79, 3.02) | 35.30 (26.96, 44.53) |  | 5.56 (4.35, 6.38) | 42.24 (32.15, 47.93) |  | 137.22 | 0.64 (0.41, 0.87) |
| South Korea | 0.63 (0.55, 0.84) | 8.89 (7.65, 12.60) |  | 2.71 (2.09, 3.34) | 8.76 (6.65, 10.42) |  | 331.7 | 0.14 (-0.06, 0.33) |
| South Sudan | 0.26 (0.15, 0.37) | 25.36 (14.21, 35.16) |  | 0.37 (0.24, 0.52) | 25.26 (15.94, 34.88) |  | 44.59 | -0.04 (-0.07, -0.00) |
| Spain | 5.30 (3.96, 6.26) | 25.39 (19.02, 30.00) |  | 8.41 (6.96, 12.14) | 17.90 (14.85, 25.69) |  | 58.46 | -1.60 (-1.74, -1.45) |
| Sri Lanka | 0.32 (0.27, 0.41) | 8.06 (6.78, 10.55) |  | 0.66 (0.48, 0.89) | 7.39 (5.52, 9.95) |  | 103.98 | 0.17 (0.03, 0.32) |
| Sudan | 0.34 (0.24, 0.48) | 9.32 (6.49, 12.98) |  | 0.87 (0.61, 1.17) | 11.40 (8.06, 15.30) |  | 152.23 | 0.68 (0.61, 0.74) |
| Suriname | 0.03 (0.03, 0.05) | 34.43 (28.10, 48.58) |  | 0.10 (0.07, 0.12) | 43.24 (32.40, 53.49) |  | 188.13 | 0.83 (0.60, 1.06) |
| Sweden | 2.30 (1.80, 2.86) | 34.74 (26.93, 43.10) |  | 3.03 (2.53, 4.05) | 27.80 (23.11, 36.99) |  | 31.3 | -1.15 (-1.40, -0.91) |
| Switzerland | 1.65 (1.03, 1.82) | 39.76 (24.79, 43.83) |  | 1.91 (1.57, 2.97) | 22.47 (18.53, 34.87) |  | 16.09 | -2.05 (-2.19, -1.92) |
| Syrian Arab Republic | 0.19 (0.13, 0.32) | 9.14 (5.72, 15.38) |  | 0.45 (0.31, 0.73) | 9.72 (6.69, 15.75) |  | 131.82 | 0.12 (-0.02, 0.26) |
| Taiwan (Province of China) | 0.50 (0.44, 0.71) | 8.13 (7.03, 11.68) |  | 1.96 (1.26, 2.54) | 10.94 (7.01, 14.15) |  | 294.63 | 1.23 (1.14, 1.32) |
| Tajikistan | 0.08 (0.07, 0.10) | 7.73 (6.37, 9.38) |  | 0.14 (0.09, 0.20) | 10.76 (6.95, 15.32) |  | 66.59 | 1.27 (1.18, 1.37) |
| Thailand | 1.23 (0.73, 1.72) | 30.18 (17.82, 41.20) |  | 2.75 (1.74, 3.79) | 31.39 (19.88, 43.00) |  | 124.57 | 0.10 (0.06, 0.14) |
| Timor-Leste | 1.13 (0.82, 1.36) | 9.85 (7.35, 11.83) |  | 3.57 (2.55, 4.73) | 8.59 (6.18, 11.32) |  | 216.49 | -0.73 (-0.86, -0.60) |
| Togo | 0.01 (0.01, 0.01) | 9.69 (6.30, 13.25) |  | 0.05 (0.03, 0.06) | 15.27 (10.30, 19.72) |  | 481.68 | 1.83 (1.69, 1.97) |
| Tokelau | 0.08 (0.07, 0.11) | 20.60 (16.35, 27.97) |  | 0.31 (0.18, 0.44) | 34.09 (19.44, 46.81) |  | 273.21 | 1.96 (1.80, 2.12) |
| Tonga | 0.00 (0.00, 0.00) | 22.00 (15.51, 31.59) |  | 0.00 (0.00, 0.00) | 23.53 (18.32, 29.32) |  | -0.55 | 0.29 (0.24, 0.34) |
| Trinidad and Tobago | 0.01 (0.00, 0.01) | 32.21 (25.79, 39.25) |  | 0.01 (0.01, 0.01) | 34.77 (26.34, 42.14) |  | 72.53 | 0.15 (-0.02, 0.33) |
| Tunisia | 0.18 (0.13, 0.20) | 55.87 (42.22, 65.51) |  | 0.40 (0.27, 0.53) | 53.20 (36.17, 69.11) |  | 125.98 | -0.56 (-0.77, -0.35) |
| Turkey | 0.17 (0.12, 0.22) | 8.78 (6.45, 11.63) |  | 0.46 (0.33, 0.65) | 9.13 (6.57, 12.71) |  | 174.69 | 0.25 (0.19, 0.30) |
| Turkmenistan | 2.10 (1.49, 2.66) | 16.17 (11.44, 20.50) |  | 5.19 (3.70, 6.59) | 14.71 (10.52, 18.66) |  | 146.72 | -0.15 (-0.68, 0.39) |
| Tuvalu | 0.05 (0.04, 0.08) | 8.34 (6.86, 14.53) |  | 0.10 (0.08, 0.18) | 8.61 (6.34, 15.17) |  | 118.63 | 0.09 (-0.00, 0.19) |
| Uganda | 0.00 (0.00, 0.00) | 28.94 (21.62, 40.87) |  | 0.00 (0.00, 0.00) | 30.02 (23.20, 38.76) |  | 93.86 | 0.03 (-0.05, 0.11) |
| Ukraine | 1.02 (0.79, 1.42) | 41.49 (32.65, 57.41) |  | 2.45 (1.94, 3.02) | 57.20 (45.87, 69.91) |  | 141.71 | 1.05 (0.88, 1.21) |
| United Arab Emirates | 3.68 (3.22, 5.50) | 16.99 (14.84, 26.56) |  | 4.95 (3.75, 7.73) | 18.24 (13.93, 29.28) |  | 34.42 | -0.18 (-0.40, 0.05) |
| United Kingdom | 0.01 (0.01, 0.02) | 12.91 (7.71, 22.40) |  | 0.13 (0.07, 0.24) | 12.81 (7.36, 24.71) |  | 768.57 | -0.39 (-0.71, -0.07) |
| Tanzania | 10.53 (8.03, 12.99) | 30.52 (23.48, 37.83) |  | 15.97 (12.53, 20.47) | 26.15 (20.39, 33.03) |  | 51.68 | -0.80 (-0.90, -0.70) |
| United States of America | 36.24 (25.66, 40.65) | 28.30 (20.18, 31.73) |  | 48.32 (41.35, 70.59) | 18.94 (16.17, 27.60) |  | 33.36 | -1.78 (-1.95, -1.61) |
| United States Virgin Islands | 0.02 (0.01, 0.03) | 71.83 (56.01, 101.06) |  | 0.06 (0.05, 0.08) | 89.54 (74.36, 111.00) |  | 232.45 | 1.15 (0.94, 1.37) |
| Uruguay | 0.55 (0.48, 0.76) | 35.57 (30.86, 49.22) |  | 0.84 (0.69, 1.07) | 35.95 (29.41, 45.50) |  | 52.56 | -0.11 (-0.43, 0.21) |
| Uzbekistan | 0.24 (0.17, 0.29) | 6.51 (4.60, 8.00) |  | 0.41 (0.31, 0.52) | 10.49 (5.79, 12.85) |  | 70.16 | 1.83 (1.45, 2.20) |
| Vanuatu | 0.01 (0.00, 0.01) | 23.77 (16.05, 34.60) |  | 0.02 (0.01, 0.02) | 28.08 (20.76, 36.72) |  | 207.94 | 0.63 (0.58, 0.69) |
| Venezuela | 0.92 (0.80, 1.40) | 25.28 (21.60, 39.72) |  | 4.09 (2.81, 5.44) | 35.41 (24.72, 47.19) |  | 346.94 | 0.60 (0.14, 1.07) |
| Viet Nam | 1.10 (0.83, 1.52) | 8.70 (6.54, 12.06) |  | 2.91 (2.27, 4.20) | 10.88 (8.52, 15.59) |  | 164.09 | 0.87 (0.83, 0.91) |
| Yemen | 0.13 (0.09, 0.19) | 9.96 (6.08, 14.51) |  | 0.54 (0.39, 0.71) | 11.92 (8.62, 15.95) |  | 301.41 | 0.86 (0.77, 0.96) |
| Zambia | 0.35 (0.20, 0.46) | 30.91 (17.62, 40.44) |  | 0.70 (0.42, 0.96) | 32.21 (18.77, 43.87) |  | 102.36 | 0.17 (0.07, 0.27) |
| Zimbabwe | 0.64 (0.52, 0.87) | 49.23 (40.75, 65.78) |  | 1.21 (0.91, 1.47) | 61.39 (46.24, 74.72) |  | 90.49 | 0.73 (0.36, 1.10) |

ASMR: age-standardized mortality rate; CI: confidence interval; EAPC: estimated annual percentage change; UI: uncertainty interval.

**Table S4**. The DALYs and ASDRs of prostate cancer in 1990 and 2019 and their temporal trends from 1990 to 2019 at the national level.

| Countries | 1990 | |  | 2019 | |  | 1990-2019 | |
| --- | --- | --- | --- | --- | --- | --- | --- | --- |
|  | DALYs  No. x 10^3^ (95% UI) | ASDR per 100, 000  No. (95% UI) |  | DALYs  No. x 10^3^ (95% UI) | ASDR per 100, 000  No. (95% UI) |  | Percentage change  of DALYs (%) | EAPC  No. (95% CI) |
| Afghanistan | 6.60 (4.95, 8.97) | 197.31 (145.04, 272.04) |  | 9.39 (7.11, 11.93) | 209.19 (157.12, 272.23) |  | 42.35 | 0.23 (0.18, 0.29) |
| Albania | 2.73 (2.11, 4.37) | 334.84 (252.69, 557.05) |  | 6.64 (4.10, 11.75) | 326.06 (205.45, 566.01) |  | 142.98 | -0.10 (-0.24, 0.03) |
| Algeria | 8.75 (6.41, 11.39) | 172.60 (130.05, 222.25) |  | 22.20 (16.36, 30.44) | 149.00 (111.13, 203.20) |  | 153.86 | -0.67 (-0.73, -0.62) |
| American Samoa | 0.06 (0.05, 0.08) | 730.63 (611.74, 943.23) |  | 0.14 (0.12, 0.17) | 731.78 (593.20, 859.78) |  | 124.4 | 0.23 (0.10, 0.35) |
| Andorra | 0.09 (0.07, 0.13) | 377.32 (287.35, 508.38) |  | 0.23 (0.17, 0.33) | 337.56 (254.12, 477.30) |  | 149.44 | -0.45 (-0.49, -0.42) |
| Angola | 6.01 (3.88, 7.95) | 423.85 (263.60, 563.48) |  | 18.44 (12.83, 23.82) | 510.30 (355.42, 655.20) |  | 207.03 | 0.61 (0.58, 0.65) |
| Antigua and Barbuda | 0.28 (0.24, 0.38) | 1248.23 (1054.49, 1666.55) |  | 0.54 (0.41, 0.67) | 1274.82 (974.44, 1581.02) |  | 89.07 | 0.01 (-0.34, 0.36) |
| Argentina | 62.98 (55.44, 89.12) | 469.68 (413.73, 670.73) |  | 106.60 (91.05, 150.68) | 461.76 (393.67, 651.60) |  | 69.25 | -0.28 (-0.54, -0.01) |
| Armenia | 2.08 (1.66, 2.64) | 200.60 (165.17, 267.18) |  | 5.33 (3.17, 6.65) | 311.76 (183.91, 385.82) |  | 156.39 | 1.58 (1.45, 1.70) |
| Australia | 41.13 (31.82, 50.66) | 494.92 (381.62, 605.83) |  | 78.47 (64.11, 112.13) | 388.14 (317.20, 552.13) |  | 90.77 | -1.27 (-1.45, -1.09) |
| Austria | 20.05 (15.42, 24.45) | 453.13 (347.99, 554.65) |  | 27.80 (22.98, 44.10) | 341.57 (283.20, 537.45) |  | 38.66 | -1.28 (-1.43, -1.13) |
| Azerbaijan | 4.02 (3.45, 5.07) | 224.84 (192.07, 292.73) |  | 8.83 (6.84, 11.75) | 254.37 (197.15, 342.70) |  | 119.5 | 0.61 (0.51, 0.72) |
| Bahamas | 0.68 (0.57, 0.92) | 1177.71 (990.85, 1622.14) |  | 1.82 (1.40, 2.45) | 1189.13 (926.03, 1608.43) |  | 168.42 | -0.02 (-0.14, 0.09) |
| Bahrain | 0.18 (0.14, 0.22) | 279.85 (209.45, 344.96) |  | 0.83 (0.61, 1.15) | 239.28 (181.32, 310.22) |  | 367.32 | -0.67 (-0.93, -0.40) |
| Bangladesh | 30.75 (21.89, 40.21) | 136.28 (96.76, 179.49) |  | 74.56 (42.73, 115.07) | 118.34 (68.94, 181.71) |  | 142.46 | -0.60 (-0.75, -0.46) |
| Barbados | 1.38 (1.12, 1.76) | 1071.30 (874.80, 1375.69) |  | 2.93 (2.15, 3.59) | 1316.94 (971.98, 1609.04) |  | 112.25 | -0.01 (-0.32, 0.31) |
| Belarus | 12.72 (10.94, 21.66) | 289.03 (247.04, 502.17) |  | 22.96 (17.05, 34.26) | 396.44 (294.75, 599.37) |  | 80.52 | 0.86 (0.68, 1.04) |
| Belgium | 34.93 (25.55, 40.76) | 571.94 (410.15, 657.99) |  | 39.20 (32.04, 65.87) | 368.84 (301.64, 619.18) |  | 12.23 | -1.86 (-2.04, -1.68) |
| Belize | 0.20 (0.16, 0.33) | 450.85 (359.37, 753.41) |  | 0.80 (0.63, 1.04) | 648.05 (509.12, 846.12) |  | 311.08 | 0.82 (0.24, 1.41) |
| Benin | 2.99 (2.43, 4.06) | 337.34 (275.07, 456.28) |  | 9.37 (5.63, 12.71) | 511.55 (307.20, 685.72) |  | 213.32 | 1.69 (1.52, 1.86) |
| Bermuda | 0.21 (0.14, 0.25) | 847.06 (583.76, 1015.53) |  | 0.46 (0.36, 0.60) | 795.19 (618.19, 1030.56) |  | 124.69 | -0.26 (-0.35, -0.18) |
| Bhutan | 0.11 (0.06, 0.16) | 121.24 (70.99, 180.94) |  | 0.38 (0.21, 0.62) | 152.05 (84.39, 242.77) |  | 265.69 | 0.91 (0.84, 0.97) |
| Bolivia | 6.18 (4.90, 7.67) | 488.87 (391.49, 607.36) |  | 22.45 (16.49, 29.00) | 605.05 (448.06, 775.97) |  | 263.45 | 0.69 (0.64, 0.75) |
| Bosnia and Herzegovina | 3.26 (2.86, 4.18) | 228.90 (200.20, 294.48) |  | 8.04 (5.52, 10.55) | 312.01 (214.02, 407.75) |  | 146.69 | 1.26 (1.04, 1.49) |
| Botswana | 1.60 (1.20, 2.09) | 746.88 (563.83, 956.62) |  | 4.18 (2.88, 5.42) | 900.94 (625.44, 1141.53) |  | 162.27 | 0.11 (-0.37, 0.60) |
| Brazil | 155.63 (139.63, 236.96) | 436.16 (386.63, 660.91) |  | 413.23 (359.72, 605.64) | 417.31 (361.07, 611.63) |  | 165.51 | -0.29 (-0.55, -0.03) |
| Brunei | 0.09 (0.07, 0.11) | 292.33 (235.06, 372.55) |  | 0.27 (0.20, 0.33) | 356.24 (234.42, 453.31) |  | 205.75 | 1.52 (1.12, 1.93) |
| Bulgaria | 14.30 (12.43, 22.45) | 256.57 (222.53, 411.76) |  | 23.33 (16.39, 29.89) | 367.48 (260.21, 468.39) |  | 63.11 | 2.03 (1.72, 2.34) |
| Burkina Faso | 5.74 (4.45, 7.81) | 318.18 (249.40, 432.06) |  | 16.64 (10.30, 22.10) | 494.85 (305.45, 652.63) |  | 190.05 | 1.84 (1.75, 1.93) |
| Burundi | 4.74 (2.49, 6.54) | 489.59 (257.77, 667.78) |  | 8.99 (5.49, 12.88) | 458.45 (285.39, 645.81) |  | 89.94 | -0.32 (-0.38, -0.27) |
| Cambodia | 3.70 (2.76, 4.63) | 235.26 (177.01, 294.47) |  | 12.37 (8.74, 15.40) | 310.54 (220.71, 384.03) |  | 234.06 | 1.05 (0.98, 1.12) |
| Cameroon | 6.57 (5.06, 8.91) | 364.85 (282.43, 498.84) |  | 27.10 (14.53, 41.03) | 590.98 (324.03, 879.34) |  | 312.27 | 1.85 (1.69, 2.01) |
| Canada | 71.68 (50.51, 81.44) | 524.67 (368.69, 592.40) |  | 106.89 (90.20, 157.89) | 327.60 (276.26, 482.34) |  | 49.11 | -2.35 (-2.63, -2.07) |
| Cape Verde | 0.35 (0.27, 0.55) | 342.92 (263.33, 535.00) |  | 1.73 (1.16, 2.11) | 1101.89 (737.43, 1338.25) |  | 390.91 | 2.45 (1.61, 3.30) |
| Central African Republic | 2.03 (1.40, 2.65) | 495.62 (341.40, 657.86) |  | 3.30 (2.19, 4.40) | 492.05 (336.50, 658.41) |  | 62.62 | -0.05 (-0.09, -0.00) |
| Chad | 3.53 (2.64, 4.96) | 274.51 (205.93, 391.72) |  | 12.04 (7.47, 16.39) | 470.38 (294.07, 632.84) |  | 241.1 | 2.20 (1.98, 2.41) |
| Chile | 16.16 (13.27, 21.32) | 399.79 (329.79, 530.45) |  | 45.85 (34.23, 54.66) | 440.00 (328.06, 522.70) |  | 183.78 | 0.13 (-0.08, 0.35) |
| China | 403.11 (306.18, 488.11) | 125.93 (99.53, 151.84) |  | 1002.59 (794.01, 1322.63) | 118.94 (95.05, 154.14) |  | 148.71 | -0.24 (-0.27, -0.20) |
| Colombia | 28.10 (22.86, 36.25) | 378.98 (308.31, 493.87) |  | 75.32 (54.13, 116.51) | 319.93 (230.02, 493.84) |  | 168.01 | -1.12 (-1.37, -0.88) |
| Comoros | 0.48 (0.25, 0.68) | 479.62 (261.78, 677.60) |  | 0.99 (0.65, 1.32) | 501.21 (327.43, 665.41) |  | 108.36 | 0.06 (-0.04, 0.15) |
| Congo | 2.04 (1.43, 2.51) | 545.47 (390.27, 672.44) |  | 4.82 (3.33, 6.17) | 528.00 (364.57, 666.09) |  | 136.32 | -0.21 (-0.37, -0.04) |
| Cook Islands | 0.06 (0.05, 0.08) | 1111.36 (931.69, 1347.61) |  | 0.11 (0.09, 0.13) | 889.20 (732.92, 1077.03) |  | 68.4 | -0.85 (-0.91, -0.79) |
| Costa Rica | 2.44 (2.03, 3.31) | 313.87 (258.94, 422.24) |  | 9.50 (5.96, 12.77) | 419.20 (263.77, 561.38) |  | 288.67 | 0.82 (0.54, 1.09) |
| Cote d'Ivoire | 5.74 (4.45, 7.78) | 355.13 (280.54, 473.37) |  | 21.73 (12.38, 31.19) | 518.99 (296.91, 737.02) |  | 278.34 | 1.52 (1.38, 1.67) |
| Croatia | 9.31 (7.16, 11.67) | 409.71 (311.03, 511.92) |  | 15.35 (10.75, 19.79) | 403.35 (282.73, 518.28) |  | 64.97 | 0.24 (0.07, 0.40) |
| Cuba | 24.68 (20.69, 32.72) | 492.24 (413.66, 656.62) |  | 56.06 (33.33, 72.32) | 622.74 (369.43, 804.08) |  | 127.12 | 0.79 (0.72, 0.86) |
| Cyprus | 1.27 (1.04, 1.67) | 343.86 (280.67, 455.27) |  | 3.31 (2.73, 4.13) | 366.70 (300.65, 445.41) |  | 160.53 | 0.19 (-0.12, 0.49) |
| Czech Republic | 20.18 (16.14, 26.61) | 375.63 (303.78, 498.69) |  | 33.70 (24.90, 43.80) | 359.77 (264.87, 463.53) |  | 67.03 | -0.19 (-0.47, 0.10) |
| Democratic Republic of the Congo | 24.35 (17.36, 32.76) | 422.46 (292.94, 569.30) |  | 51.53 (35.16, 69.01) | 444.72 (300.58, 597.26) |  | 111.67 | 0.10 (0.05, 0.15) |
| Denmark | 20.87 (15.91, 25.18) | 588.45 (451.57, 711.13) |  | 27.70 (21.62, 35.46) | 498.03 (389.86, 634.90) |  | 32.68 | -0.47 (-0.70, -0.23) |
| Djibouti | 0.27 (0.16, 0.39) | 496.49 (288.39, 697.17) |  | 1.47 (0.93, 2.10) | 566.23 (362.09, 782.74) |  | 444.9 | 0.42 (0.37, 0.47) |
| Dominica | 0.48 (0.41, 0.63) | 1591.53 (1358.78, 2061.22) |  | 0.82 (0.61, 1.02) | 1923.95 (1428.24, 2394.31) |  | 69.94 | 0.52 (0.37, 0.66) |
| Dominican Republic | 7.91 (5.44, 14.10) | 486.84 (333.94, 865.05) |  | 28.83 (18.90, 47.68) | 700.05 (464.76, 1151.43) |  | 264.37 | 1.50 (0.99, 2.02) |
| Ecuador | 8.41 (7.38, 10.91) | 361.16 (315.01, 465.91) |  | 26.70 (20.05, 36.24) | 406.53 (302.93, 548.65) |  | 217.39 | 0.60 (0.40, 0.81) |
| Egypt | 12.77 (10.83, 15.91) | 105.13 (89.53, 135.24) |  | 35.59 (25.03, 49.72) | 119.06 (85.66, 166.01) |  | 178.73 | 0.35 (0.23, 0.48) |
| El Salvador | 2.93 (2.42, 4.21) | 230.57 (190.20, 331.95) |  | 8.69 (6.27, 11.39) | 341.09 (245.75, 448.29) |  | 196.78 | 0.88 (0.45, 1.30) |
| Equatorial Guinea | 0.30 (0.19, 0.40) | 429.50 (266.52, 587.22) |  | 0.84 (0.56, 1.14) | 552.30 (368.73, 729.86) |  | 181.06 | 0.94 (0.81, 1.07) |
| Eritrea | 1.49 (0.62, 2.17) | 476.03 (192.22, 681.84) |  | 4.39 (2.43, 6.01) | 512.76 (285.06, 698.76) |  | 193.85 | -0.05 (-0.30, 0.21) |
| Estonia | 2.62 (1.94, 3.07) | 381.33 (279.73, 454.09) |  | 5.22 (2.74, 6.88) | 524.94 (274.30, 689.75) |  | 99.67 | 1.46 (1.21, 1.72) |
| Eswatini | 0.72 (0.56, 0.91) | 717.84 (563.05, 890.99) |  | 1.59 (1.13, 2.07) | 884.34 (628.98, 1127.47) |  | 120.62 | 0.86 (0.56, 1.16) |
| Ethiopia | 18.97 (13.54, 27.47) | 206.50 (143.58, 292.76) |  | 43.41 (22.75, 72.73) | 235.34 (124.85, 388.12) |  | 128.82 | 0.52 (0.40, 0.64) |
| Federated States of Micronesia | 0.08 (0.06, 0.12) | 457.87 (345.42, 660.21) |  | 0.12 (0.08, 0.17) | 518.04 (368.65, 671.30) |  | 43.7 | 0.48 (0.38, 0.58) |
| Fiji | 0.46 (0.24, 0.65) | 349.83 (189.77, 495.98) |  | 1.00 (0.44, 1.46) | 386.23 (178.32, 551.31) |  | 116.65 | 0.60 (0.42, 0.78) |
| Finland | 12.11 (9.18, 14.59) | 465.78 (353.68, 559.35) |  | 22.39 (18.30, 31.90) | 391.69 (319.41, 553.75) |  | 84.85 | -1.06 (-1.26, -0.86) |
| France | 185.19 (130.02, 208.37) | 550.79 (383.67, 614.58) |  | 215.94 (175.63, 342.89) | 341.66 (278.15, 544.92) |  | 16.61 | -1.98 (-2.20, -1.76) |
| Gabon | 1.08 (0.76, 1.39) | 523.02 (371.39, 653.74) |  | 2.28 (1.45, 3.10) | 612.03 (397.27, 808.53) |  | 110.76 | 0.46 (0.41, 0.51) |
| Gambia | 0.21 (0.16, 0.29) | 142.74 (111.75, 193.88) |  | 0.71 (0.52, 0.96) | 179.98 (131.49, 240.74) |  | 234.64 | 0.71 (0.59, 0.82) |
| Georgia | 5.65 (4.51, 7.25) | 250.51 (205.67, 337.79) |  | 8.91 (5.17, 11.00) | 371.50 (216.82, 457.45) |  | 57.89 | 2.67 (2.04, 3.30) |
| Germany | 200.12 (151.72, 246.32) | 433.93 (331.94, 532.36) |  | 331.70 (254.78, 426.68) | 369.00 (279.87, 463.71) |  | 65.75 | -0.89 (-1.04, -0.75) |
| Ghana | 18.14 (12.76, 24.21) | 742.04 (534.53, 968.99) |  | 42.63 (33.83, 55.26) | 732.11 (587.95, 941.93) |  | 135 | -0.33 (-0.53, -0.14) |
| Greece | 24.83 (20.84, 33.11) | 360.23 (301.22, 477.85) |  | 37.84 (31.72, 54.46) | 313.99 (263.78, 461.76) |  | 52.39 | -0.96 (-1.21, -0.72) |
| Greenland | 0.03 (0.02, 0.04) | 241.60 (193.74, 348.81) |  | 0.08 (0.06, 0.10) | 239.99 (188.36, 302.91) |  | 157.02 | -0.05 (-0.13, 0.02) |
| Grenada | 0.38 (0.33, 0.54) | 1222.82 (1037.82, 1719.22) |  | 0.73 (0.49, 0.85) | 1596.00 (1055.78, 1834.34) |  | 91.18 | 0.87 (0.12, 1.62) |
| Guam | 0.10 (0.08, 0.12) | 348.47 (283.76, 443.53) |  | 0.23 (0.19, 0.30) | 276.59 (220.01, 351.16) |  | 143.8 | -0.64 (-0.94, -0.33) |
| Guatemala | 3.92 (2.69, 8.83) | 271.81 (189.59, 609.30) |  | 20.38 (15.20, 28.08) | 459.15 (342.51, 610.09) |  | 419.76 | 1.52 (0.88, 2.17) |
| Guinea | 7.23 (4.29, 9.48) | 477.82 (281.67, 624.56) |  | 14.47 (7.61, 22.30) | 592.84 (313.73, 902.64) |  | 100.08 | 1.00 (0.84, 1.15) |
| Guinea-Bissau | 0.66 (0.47, 0.98) | 383.70 (281.33, 547.25) |  | 1.50 (0.81, 2.29) | 582.19 (320.29, 863.33) |  | 126.23 | 1.80 (1.65, 1.95) |
| Guyana | 1.42 (1.14, 2.07) | 919.95 (747.33, 1318.75) |  | 2.43 (1.80, 3.42) | 999.51 (754.19, 1392.38) |  | 70.75 | 0.20 (0.06, 0.35) |
| Haiti | 11.44 (8.13, 14.85) | 907.20 (636.69, 1184.79) |  | 25.36 (17.33, 35.66) | 943.94 (653.49, 1292.24) |  | 121.66 | 0.14 (0.10, 0.18) |
| Honduras | 2.27 (1.67, 3.12) | 258.26 (187.36, 358.38) |  | 9.19 (6.77, 13.40) | 363.43 (267.02, 533.77) |  | 304.7 | 1.73 (1.39, 2.07) |
| Hungary | 22.79 (18.37, 29.19) | 395.79 (316.30, 504.60) |  | 27.75 (21.09, 36.17) | 353.55 (268.42, 461.05) |  | 21.76 | -0.69 (-0.89, -0.50) |
| Iceland | 0.66 (0.50, 0.80) | 501.98 (381.28, 609.03) |  | 1.03 (0.82, 1.32) | 382.88 (306.63, 491.16) |  | 56.87 | -1.07 (-1.31, -0.84) |
| India | 220.76 (160.74, 260.31) | 126.26 (93.17, 148.31) |  | 580.13 (465.81, 761.35) | 120.60 (97.64, 158.10) |  | 162.78 | -0.30 (-0.38, -0.21) |
| Indonesia | 62.39 (48.44, 75.45) | 167.19 (130.16, 202.83) |  | 215.90 (149.90, 270.17) | 270.69 (190.54, 337.75) |  | 246.07 | 1.75 (1.66, 1.83) |
| Iran | 19.29 (15.18, 23.62) | 181.86 (142.80, 227.31) |  | 67.73 (50.16, 76.61) | 208.08 (155.18, 235.68) |  | 251.1 | 0.53 (0.33, 0.74) |
| Iraq | 4.74 (3.34, 6.74) | 143.75 (101.38, 205.60) |  | 16.16 (12.16, 20.98) | 183.27 (138.15, 241.88) |  | 241.1 | 0.59 (0.45, 0.74) |
| Ireland | 8.87 (6.43, 10.46) | 485.93 (359.76, 583.05) |  | 13.37 (10.94, 18.51) | 378.32 (308.56, 523.05) |  | 50.7 | -1.21 (-1.47, -0.96) |
| Israel | 6.86 (5.81, 9.24) | 305.42 (258.09, 410.15) |  | 12.10 (9.81, 20.17) | 226.40 (184.59, 374.86) |  | 76.38 | -1.54 (-1.83, -1.25) |
| Italy | 126.96 (106.08, 169.28) | 337.30 (282.81, 451.69) |  | 179.81 (150.10, 265.65) | 267.02 (224.46, 395.26) |  | 41.63 | -1.07 (-1.26, -0.88) |
| Jamaica | 4.75 (3.82, 6.05) | 569.46 (460.33, 725.79) |  | 14.09 (7.84, 18.29) | 1019.47 (567.38, 1321.70) |  | 196.52 | 1.98 (1.38, 2.59) |
| Japan | 103.13 (86.64, 140.27) | 154.27 (128.39, 208.91) |  | 245.08 (191.46, 321.32) | 139.35 (110.52, 185.53) |  | 137.64 | -0.33 (-0.44, -0.23) |
| Jordan | 0.88 (0.68, 1.12) | 174.70 (136.42, 221.35) |  | 4.85 (3.42, 6.32) | 181.09 (126.84, 234.84) |  | 450.84 | 0.23 (0.03, 0.42) |
| Kazakhstan | 8.49 (6.32, 10.60) | 204.62 (150.51, 260.57) |  | 13.08 (10.18, 17.39) | 214.25 (168.16, 287.97) |  | 54.12 | 0.62 (0.46, 0.78) |
| Kenya | 10.39 (5.37, 18.56) | 294.00 (153.52, 517.35) |  | 39.15 (25.39, 57.79) | 466.49 (312.18, 661.12) |  | 276.69 | 1.94 (1.72, 2.17) |
| Kiribati | 0.04 (0.03, 0.05) | 312.20 (234.47, 423.85) |  | 0.06 (0.04, 0.07) | 266.72 (198.61, 352.40) |  | 40.27 | -0.62 (-0.83, -0.41) |
| Kuwait | 0.36 (0.28, 0.46) | 129.35 (98.97, 164.72) |  | 1.93 (1.42, 2.60) | 164.72 (121.39, 218.92) |  | 429.68 | 0.79 (0.48, 1.11) |
| Kyrgyzstan | 1.96 (0.95, 2.33) | 180.73 (88.42, 212.74) |  | 2.18 (1.62, 2.73) | 134.24 (97.03, 164.81) |  | 10.89 | -1.36 (-1.61, -1.11) |
| Lao | 1.74 (1.22, 2.25) | 212.11 (149.62, 272.37) |  | 3.93 (2.81, 4.94) | 234.84 (168.89, 291.21) |  | 125.05 | 0.23 (0.18, 0.28) |
| Latvia | 4.27 (3.37, 5.38) | 354.39 (278.29, 445.53) |  | 7.26 (4.18, 9.42) | 495.80 (286.41, 643.31) |  | 70.01 | 1.73 (1.35, 2.11) |
| Lebanon | 3.28 (2.50, 4.10) | 350.49 (263.89, 436.65) |  | 9.81 (6.23, 13.91) | 416.55 (266.45, 591.22) |  | 198.95 | 0.93 (0.79, 1.07) |
| Lesotho | 2.34 (1.70, 3.07) | 654.21 (483.16, 841.82) |  | 4.03 (2.98, 5.10) | 966.72 (725.95, 1196.29) |  | 72.32 | 1.36 (1.10, 1.63) |
| Liberia | 1.81 (1.41, 2.52) | 326.59 (256.11, 456.25) |  | 3.90 (2.38, 5.55) | 474.11 (287.48, 663.42) |  | 115.26 | 1.67 (1.49, 1.85) |
| Libya | 1.29 (0.83, 1.74) | 155.29 (100.01, 210.89) |  | 3.73 (2.74, 4.88) | 170.03 (126.11, 221.75) |  | 188.51 | 0.33 (0.25, 0.41) |
| Lithuania | 5.35 (4.32, 7.05) | 325.74 (260.59, 427.83) |  | 10.51 (5.86, 13.29) | 491.04 (273.69, 622.73) |  | 96.43 | 1.92 (1.35, 2.49) |
| Luxembourg | 1.07 (0.73, 1.23) | 507.37 (343.23, 580.09) |  | 1.37 (1.07, 2.25) | 301.58 (235.44, 492.88) |  | 28.32 | -2.04 (-2.15, -1.94) |
| Macedonia | 2.14 (1.59, 3.52) | 264.43 (192.03, 443.70) |  | 5.40 (4.14, 7.18) | 374.61 (289.31, 492.65) |  | 152.03 | 1.41 (1.23, 1.58) |
| Madagascar | 9.70 (5.60, 13.13) | 413.87 (237.75, 555.79) |  | 16.76 (9.98, 24.32) | 397.34 (237.96, 562.54) |  | 72.77 | -0.36 (-0.51, -0.20) |
| Malawi | 4.16 (2.93, 6.42) | 265.68 (190.48, 410.59) |  | 9.22 (7.17, 12.96) | 333.00 (262.22, 474.67) |  | 121.52 | 0.95 (0.82, 1.09) |
| Malaysia | 6.06 (4.70, 7.32) | 159.90 (123.02, 192.89) |  | 21.07 (14.54, 27.75) | 180.62 (124.53, 239.73) |  | 247.38 | 0.08 (-0.08, 0.25) |
| Maldives | 0.05 (0.04, 0.07) | 122.26 (90.12, 177.97) |  | 0.17 (0.13, 0.23) | 126.80 (98.01, 167.66) |  | 241.81 | -0.06 (-0.14, 0.02) |
| Mali | 3.28 (2.59, 4.07) | 185.82 (148.39, 232.99) |  | 8.51 (6.51, 11.13) | 223.62 (171.67, 289.02) |  | 159.87 | 0.66 (0.61, 0.71) |
| Malta | 0.56 (0.44, 0.72) | 312.61 (246.41, 403.68) |  | 0.98 (0.80, 1.50) | 223.49 (180.93, 340.35) |  | 76.9 | -1.24 (-1.40, -1.09) |
| Marshall Islands | 0.03 (0.02, 0.05) | 525.80 (367.78, 752.19) |  | 0.06 (0.04, 0.08) | 472.51 (352.35, 611.62) |  | 80.2 | -0.07 (-0.32, 0.17) |
| Mauritania | 1.49 (1.11, 2.06) | 350.19 (263.36, 483.28) |  | 4.31 (2.69, 5.70) | 464.35 (291.28, 606.30) |  | 189.31 | 1.32 (1.16, 1.49) |
| Mauritius | 0.56 (0.47, 0.81) | 203.36 (173.21, 296.88) |  | 1.75 (1.19, 2.24) | 244.77 (165.73, 310.77) |  | 213.53 | 0.18 (-0.15, 0.52) |
| Mexico | 55.96 (45.92, 73.16) | 308.07 (255.73, 404.41) |  | 161.55 (122.76, 219.51) | 319.46 (244.06, 432.48) |  | 188.67 | -0.11 (-0.23, 0.01) |
| Moldova | 3.59 (3.08, 4.91) | 213.55 (185.95, 303.02) |  | 6.56 (4.52, 7.98) | 279.09 (195.33, 341.51) |  | 82.55 | 1.82 (1.33, 2.32) |
| Monaco | 0.13 (0.10, 0.18) | 420.52 (324.07, 560.58) |  | 0.18 (0.14, 0.25) | 384.81 (305.86, 533.60) |  | 36.97 | -0.24 (-0.31, -0.17) |
| Mongolia | 0.56 (0.43, 0.80) | 136.66 (107.10, 191.49) |  | 1.14 (0.87, 1.57) | 144.03 (113.39, 194.38) |  | 104.45 | -0.06 (-0.20, 0.09) |
| Montenegro | 0.90 (0.73, 1.16) | 362.83 (294.27, 469.26) |  | 1.88 (1.46, 2.36) | 437.59 (340.80, 547.17) |  | 109.81 | 0.78 (0.71, 0.85) |
| Morocco | 7.22 (5.41, 9.07) | 125.11 (91.99, 161.31) |  | 21.44 (15.51, 27.94) | 164.03 (120.38, 215.16) |  | 197.12 | 0.67 (0.33, 1.01) |
| Mozambique | 3.69 (2.58, 6.06) | 159.53 (117.18, 247.02) |  | 8.35 (5.55, 14.36) | 218.14 (152.16, 351.29) |  | 126.4 | 1.26 (1.19, 1.33) |
| Myanmar | 17.97 (13.01, 22.50) | 201.75 (149.35, 248.68) |  | 40.08 (28.68, 50.85) | 239.61 (173.09, 300.27) |  | 123 | 0.62 (0.60, 0.65) |
| Namibia | 1.42 (1.02, 2.00) | 463.39 (329.56, 675.11) |  | 3.81 (2.55, 4.91) | 740.17 (505.66, 933.96) |  | 168.36 | 2.00 (1.86, 2.14) |
| Nauru | 0.01 (0.00, 0.01) | 469.86 (358.40, 628.98) |  | 0.01 (0.00, 0.01) | 545.07 (369.50, 778.25) |  | -1.04 | 0.53 (0.38, 0.69) |
| Nepal | 4.18 (2.55, 5.95) | 110.82 (66.19, 157.16) |  | 12.90 (7.66, 19.62) | 139.88 (83.76, 211.44) |  | 208.38 | 0.84 (0.68, 1.00) |
| Netherlands | 39.00 (29.63, 48.31) | 484.11 (364.83, 593.96) |  | 69.06 (52.59, 84.88) | 425.82 (327.38, 520.63) |  | 77.09 | -0.85 (-1.02, -0.68) |
| New Zealand | 9.40 (6.91, 11.29) | 555.33 (412.41, 672.41) |  | 15.19 (12.66, 21.64) | 402.52 (336.58, 573.91) |  | 61.53 | -1.60 (-1.76, -1.43) |
| Nicaragua | 2.36 (1.96, 3.19) | 410.91 (338.69, 561.79) |  | 8.58 (6.60, 11.21) | 518.19 (403.75, 680.39) |  | 262.93 | 0.65 (0.47, 0.84) |
| Niger | 3.22 (2.48, 4.43) | 277.43 (213.71, 384.86) |  | 13.45 (8.37, 19.37) | 442.08 (275.70, 624.33) |  | 318.05 | 1.97 (1.82, 2.13) |
| Nigeria | 117.21 (53.12, 176.16) | 643.36 (298.53, 956.61) |  | 272.44 (123.95, 417.16) | 815.76 (376.40, 1234.16) |  | 132.43 | 1.09 (0.97, 1.22) |
| Niue | 0.00 (0.00, 0.01) | 446.51 (364.31, 585.65) |  | 0.00 (0.00, 0.01) | 512.75 (387.32, 620.81) |  | 16.12 | 0.61 (0.56, 0.66) |
| North Korea | 6.95 (4.93, 9.17) | 153.27 (109.10, 203.74) |  | 17.09 (13.01, 20.75) | 154.82 (118.01, 186.62) |  | 145.97 | 0.16 (0.07, 0.25) |
| Northern Mariana Islands | 0.02 (0.02, 0.03) | 343.79 (270.40, 503.24) |  | 0.09 (0.07, 0.11) | 439.47 (356.21, 518.02) |  | 295.87 | 1.22 (0.99, 1.44) |
| Norway | 17.63 (13.02, 20.79) | 579.48 (428.65, 682.75) |  | 21.94 (17.86, 29.33) | 477.42 (388.85, 638.80) |  | 24.44 | -0.89 (-1.09, -0.69) |
| Oman | 0.45 (0.30, 0.63) | 182.09 (121.85, 255.38) |  | 1.23 (0.97, 1.65) | 195.22 (158.49, 248.09) |  | 172.84 | 0.40 (0.27, 0.52) |
| Pakistan | 50.40 (36.07, 67.44) | 173.57 (125.50, 235.69) |  | 95.91 (68.73, 127.16) | 205.11 (150.59, 268.11) |  | 90.31 | 0.58 (0.43, 0.73) |
| Palau | 0.02 (0.02, 0.03) | 581.13 (450.39, 775.49) |  | 0.05 (0.04, 0.06) | 558.53 (442.88, 734.49) |  | 90.27 | -0.09 (-0.16, -0.02) |
| Palestine | 1.00 (0.68, 1.38) | 290.65 (197.54, 402.56) |  | 2.35 (1.59, 2.97) | 282.29 (184.39, 358.84) |  | 135.8 | -0.18 (-0.29, -0.06) |
| Panama | 2.36 (2.00, 3.23) | 336.15 (286.81, 462.93) |  | 8.03 (5.37, 10.75) | 411.70 (275.72, 550.04) |  | 240.2 | 0.38 (-0.02, 0.79) |
| Papua New Guinea | 2.05 (1.25, 3.08) | 281.20 (174.21, 419.96) |  | 6.56 (4.46, 9.09) | 362.53 (245.00, 496.53) |  | 220.14 | 0.99 (0.93, 1.04) |
| Paraguay | 2.66 (2.20, 3.49) | 281.50 (233.01, 370.70) |  | 9.91 (6.45, 13.59) | 415.30 (268.96, 568.11) |  | 272.42 | 1.21 (0.83, 1.60) |
| Peru | 18.12 (14.81, 24.49) | 349.48 (283.82, 473.70) |  | 51.88 (37.35, 71.11) | 345.80 (248.21, 473.45) |  | 186.32 | 0.19 (0.05, 0.33) |
| Philippines | 33.99 (25.68, 40.46) | 285.27 (217.73, 339.26) |  | 83.15 (55.80, 111.34) | 284.74 (191.38, 376.36) |  | 144.62 | -0.20 (-0.40, 0.00) |
| Poland | 53.60 (47.27, 75.71) | 320.66 (284.53, 463.45) |  | 112.70 (70.84, 142.25) | 387.69 (244.89, 488.63) |  | 110.26 | 0.53 (0.26, 0.81) |
| Portugal | 27.39 (21.13, 33.61) | 482.66 (366.57, 589.28) |  | 40.97 (34.20, 60.99) | 372.39 (311.22, 553.29) |  | 49.56 | -1.38 (-1.58, -1.18) |
| Puerto Rico | 8.58 (6.40, 10.28) | 519.07 (384.25, 617.96) |  | 13.67 (10.15, 20.56) | 407.34 (302.11, 611.15) |  | 59.36 | -1.20 (-1.32, -1.07) |
| Qatar | 0.08 (0.06, 0.12) | 226.66 (170.24, 300.16) |  | 0.85 (0.56, 1.28) | 266.92 (193.09, 362.39) |  | 908.59 | 0.78 (0.46, 1.10) |
| Romania | 25.24 (22.08, 35.86) | 215.70 (190.76, 313.64) |  | 47.18 (32.02, 58.13) | 292.78 (198.45, 360.23) |  | 86.97 | 1.00 (0.88, 1.11) |
| Russia | 122.02 (106.21, 184.43) | 217.67 (189.81, 335.91) |  | 272.50 (175.14, 335.13) | 315.98 (206.47, 387.35) |  | 123.34 | 1.52 (1.37, 1.66) |
| Rwanda | 6.03 (3.19, 8.53) | 522.99 (276.27, 731.11) |  | 11.21 (7.26, 15.31) | 542.25 (354.93, 726.63) |  | 85.96 | -0.40 (-0.66, -0.14) |
| Saint Kitts and Nevis | 0.22 (0.18, 0.29) | 1298.11 (1070.93, 1713.65) |  | 0.42 (0.34, 0.52) | 1607.79 (1280.37, 1962.70) |  | 93.79 | 0.38 (0.22, 0.55) |
| Saint Lucia | 0.42 (0.36, 0.57) | 1214.43 (1034.72, 1616.43) |  | 1.14 (0.92, 1.53) | 1221.84 (990.29, 1633.55) |  | 168.74 | -0.49 (-0.70, -0.28) |
| Saint Vincent and the Grenadines | 0.31 (0.26, 0.46) | 1036.05 (872.87, 1541.07) |  | 0.81 (0.63, 1.01) | 1249.42 (972.18, 1548.15) |  | 162.52 | 0.26 (0.03, 0.50) |
| Samoa | 0.11 (0.08, 0.13) | 295.50 (225.58, 357.99) |  | 0.15 (0.11, 0.18) | 240.22 (184.47, 290.50) |  | 34.4 | -0.66 (-0.86, -0.46) |
| San Marino | 0.06 (0.05, 0.08) | 447.15 (357.56, 559.99) |  | 0.13 (0.09, 0.20) | 412.00 (270.59, 633.37) |  | 103.02 | -0.36 (-0.40, -0.31) |
| Sao Tome and Principe | 0.07 (0.05, 0.10) | 268.16 (211.60, 360.73) |  | 0.16 (0.12, 0.21) | 384.23 (288.74, 507.77) |  | 120.68 | 1.45 (1.34, 1.56) |
| Saudi Arabia | 4.33 (2.61, 6.79) | 164.78 (98.82, 260.34) |  | 11.35 (8.49, 16.85) | 148.35 (111.71, 222.26) |  | 161.98 | -0.86 (-1.09, -0.62) |
| Senegal | 5.20 (3.83, 7.28) | 359.69 (271.52, 500.91) |  | 17.70 (11.09, 23.78) | 571.85 (354.97, 762.55) |  | 240.38 | 1.98 (1.78, 2.19) |
| Serbia | 12.98 (10.42, 16.43) | 275.30 (220.65, 352.51) |  | 26.25 (18.73, 33.46) | 352.16 (255.41, 447.80) |  | 102.28 | 1.21 (0.98, 1.45) |
| Seychelles | 0.15 (0.11, 0.24) | 660.85 (478.33, 1034.82) |  | 0.41 (0.31, 0.51) | 941.24 (700.41, 1138.93) |  | 171.35 | 0.97 (0.64, 1.30) |
| Sierra Leone | 2.91 (2.24, 4.06) | 314.50 (243.55, 437.64) |  | 7.14 (4.44, 10.02) | 474.43 (293.27, 655.20) |  | 145.57 | 1.86 (1.68, 2.03) |
| Singapore | 1.28 (0.99, 1.64) | 155.70 (125.66, 206.98) |  | 4.58 (3.52, 5.99) | 136.30 (103.74, 176.18) |  | 257.35 | -0.56 (-0.71, -0.41) |
| Slovakia | 7.51 (6.40, 9.21) | 306.76 (264.97, 379.99) |  | 13.05 (8.64, 17.46) | 349.33 (231.30, 460.28) |  | 73.75 | 0.63 (0.49, 0.77) |
| Slovenia | 3.36 (2.47, 4.62) | 377.18 (279.22, 513.11) |  | 8.54 (5.56, 11.31) | 447.57 (291.02, 588.71) |  | 154.39 | 0.64 (0.32, 0.96) |
| Solomon Islands | 0.23 (0.17, 0.34) | 389.60 (270.60, 562.20) |  | 0.53 (0.39, 0.68) | 448.60 (337.98, 564.07) |  | 124.02 | 0.56 (0.50, 0.61) |
| Somalia | 4.29 (2.29, 6.35) | 468.00 (249.39, 685.05) |  | 10.13 (5.89, 15.26) | 453.08 (268.76, 676.10) |  | 136.15 | -0.10 (-0.16, -0.04) |
| South Africa | 42.56 (31.94, 56.54) | 560.36 (421.69, 732.21) |  | 104.78 (84.45, 122.93) | 666.08 (522.63, 764.61) |  | 146.2 | 0.63 (0.38, 0.88) |
| South Korea | 12.99 (11.31, 17.12) | 140.81 (123.42, 191.53) |  | 48.81 (38.72, 62.26) | 134.94 (105.49, 168.11) |  | 275.66 | 0.12 (-0.06, 0.30) |
| South Sudan | 5.27 (3.01, 7.61) | 444.61 (252.14, 633.06) |  | 7.35 (4.60, 10.57) | 433.63 (273.26, 614.13) |  | 39.5 | -0.12 (-0.15, -0.09) |
| Spain | 89.39 (66.59, 106.62) | 391.83 (290.97, 465.04) |  | 125.14 (104.65, 185.47) | 281.48 (236.10, 419.38) |  | 39.99 | -1.52 (-1.68, -1.36) |
| Sri Lanka | 5.91 (4.96, 7.42) | 127.67 (107.69, 162.15) |  | 12.36 (8.91, 16.86) | 119.75 (87.62, 160.97) |  | 108.94 | 0.20 (0.07, 0.32) |
| Sudan | 6.44 (4.57, 8.89) | 149.27 (105.90, 208.21) |  | 15.39 (10.62, 20.88) | 180.23 (124.69, 243.36) |  | 138.95 | 0.62 (0.55, 0.69) |
| Suriname | 0.56 (0.48, 0.77) | 522.68 (437.07, 722.36) |  | 1.67 (1.24, 2.09) | 679.24 (507.49, 846.60) |  | 196.07 | 0.92 (0.69, 1.16) |
| Sweden | 38.90 (29.12, 46.75) | 567.01 (423.96, 677.43) |  | 47.80 (39.96, 63.31) | 455.05 (381.47, 595.61) |  | 22.86 | -1.10 (-1.34, -0.85) |
| Switzerland | 27.59 (17.42, 30.50) | 635.62 (401.12, 702.78) |  | 30.35 (24.54, 48.35) | 367.80 (296.84, 584.96) |  | 10.01 | -1.98 (-2.06, -1.89) |
| Syrian Arab Republic | 3.62 (2.44, 5.60) | 151.37 (99.07, 242.83) |  | 8.71 (6.00, 13.82) | 159.01 (109.84, 254.73) |  | 140.49 | -0.00 (-0.17, 0.17) |
| Taiwan (Province of China) | 10.10 (8.82, 14.17) | 136.34 (119.26, 193.11) |  | 33.54 (22.37, 44.20) | 185.30 (122.90, 241.61) |  | 232.08 | 1.26 (1.16, 1.37) |
| Tajikistan | 1.63 (1.30, 1.93) | 143.36 (115.75, 169.77) |  | 2.81 (2.09, 4.25) | 168.56 (113.55, 247.93) |  | 73.15 | 0.73 (0.60, 0.87) |
| Thailand | 25.23 (15.01, 35.49) | 527.58 (315.28, 731.69) |  | 54.78 (34.46, 75.94) | 543.76 (344.90, 751.20) |  | 117.15 | 0.08 (0.04, 0.11) |
| Timor-Leste | 22.47 (15.54, 27.16) | 162.09 (116.26, 194.56) |  | 62.55 (43.75, 85.56) | 141.81 (99.98, 192.55) |  | 178.4 | -0.77 (-0.93, -0.62) |
| Togo | 0.16 (0.10, 0.23) | 156.94 (100.89, 217.41) |  | 0.85 (0.55, 1.12) | 239.94 (159.06, 313.27) |  | 427.16 | 1.70 (1.56, 1.85) |
| Tokelau | 1.64 (1.29, 2.23) | 339.56 (267.37, 459.66) |  | 6.48 (3.62, 9.33) | 554.59 (314.75, 780.31) |  | 294.52 | 1.89 (1.74, 2.05) |
| Tonga | 0.00 (0.00, 0.00) | 338.04 (240.43, 484.39) |  | 0.00 (0.00, 0.00) | 355.07 (276.83, 445.28) |  | 1.1 | 0.23 (0.19, 0.28) |
| Trinidad and Tobago | 0.11 (0.09, 0.14) | 493.78 (390.97, 607.52) |  | 0.18 (0.14, 0.22) | 536.75 (407.81, 653.08) |  | 57.72 | 0.20 (0.02, 0.37) |
| Tunisia | 3.27 (2.30, 3.70) | 894.89 (646.17, 1016.13) |  | 7.08 (4.66, 9.52) | 847.97 (562.92, 1131.40) |  | 116.38 | -0.61 (-0.82, -0.41) |
| Turkey | 3.08 (2.28, 4.04) | 134.91 (99.75, 177.86) |  | 8.22 (5.84, 11.75) | 146.83 (104.47, 208.21) |  | 167.31 | 0.34 (0.30, 0.39) |
| Turkmenistan | 38.14 (26.98, 48.16) | 265.04 (188.97, 334.86) |  | 89.47 (63.88, 116.48) | 236.42 (168.68, 303.50) |  | 134.56 | -0.24 (-0.66, 0.18) |
| Tuvalu | 1.06 (0.91, 1.70) | 147.95 (123.29, 251.34) |  | 2.39 (1.79, 3.99) | 153.59 (113.77, 270.81) |  | 124.39 | 0.17 (0.06, 0.29) |
| Uganda | 0.01 (0.01, 0.02) | 464.62 (354.92, 643.06) |  | 0.02 (0.01, 0.02) | 459.11 (353.18, 600.12) |  | 65.55 | -0.12 (-0.22, -0.03) |
| Ukraine | 20.80 (15.90, 29.29) | 739.56 (570.04, 1036.70) |  | 50.92 (39.56, 63.41) | 1003.66 (788.84, 1234.96) |  | 144.8 | 0.96 (0.78, 1.14) |
| United Arab Emirates | 77.58 (67.06, 110.72) | 317.45 (278.61, 468.98) |  | 104.01 (80.15, 151.17) | 363.53 (280.57, 536.43) |  | 34.07 | 0.07 (-0.15, 0.29) |
| United Kingdom | 0.38 (0.24, 0.62) | 213.70 (128.27, 368.26) |  | 4.00 (2.15, 7.47) | 215.04 (125.12, 415.27) |  | 959.22 | -0.33 (-0.64, -0.01) |
| Tanzania | 181.19 (135.85, 218.41) | 479.45 (362.84, 586.63) |  | 248.19 (204.32, 325.41) | 409.51 (337.27, 535.35) |  | 36.98 | -0.82 (-0.91, -0.73) |
| United States of America | 710.32 (490.60, 793.17) | 525.41 (363.95, 586.15) |  | 926.63 (801.16, 1360.98) | 358.66 (310.15, 526.40) |  | 30.45 | -1.70 (-1.86, -1.54) |
| United States Virgin Islands | 0.37 (0.28, 0.53) | 1133.74 (868.36, 1596.65) |  | 1.22 (1.00, 1.53) | 1483.09 (1217.12, 1838.39) |  | 229.88 | 1.42 (1.13, 1.70) |
| Uruguay | 9.51 (8.16, 13.04) | 567.20 (487.79, 777.02) |  | 13.27 (10.86, 16.85) | 571.65 (467.49, 725.82) |  | 39.54 | -0.12 (-0.46, 0.21) |
| Uzbekistan | 4.65 (3.33, 5.59) | 112.58 (79.58, 135.85) |  | 9.11 (7.06, 12.81) | 159.36 (103.69, 192.30) |  | 96.21 | 1.40 (1.11, 1.68) |
| Vanuatu | 0.11 (0.08, 0.16) | 366.78 (252.37, 534.83) |  | 0.33 (0.24, 0.44) | 432.17 (317.62, 571.27) |  | 203.79 | 0.59 (0.54, 0.64) |
| Venezuela | 17.18 (15.24, 24.94) | 428.70 (377.65, 640.14) |  | 77.07 (50.48, 103.62) | 613.58 (410.99, 821.08) |  | 348.63 | 0.79 (0.36, 1.22) |
| Viet Nam | 20.75 (15.65, 28.67) | 141.07 (106.39, 193.27) |  | 54.79 (41.44, 78.42) | 173.05 (133.96, 248.57) |  | 164.03 | 0.78 (0.75, 0.81) |
| Yemen | 2.80 (1.83, 4.00) | 160.03 (101.38, 232.19) |  | 10.18 (7.40, 13.69) | 190.60 (139.40, 253.99) |  | 263.35 | 0.85 (0.75, 0.94) |
| Zambia | 6.98 (4.08, 9.22) | 537.78 (312.80, 703.20) |  | 14.58 (8.63, 20.11) | 566.14 (334.59, 780.28) |  | 108.97 | 0.18 (0.05, 0.31) |
| Zimbabwe | 13.18 (10.63, 18.24) | 811.95 (664.10, 1104.21) |  | 25.01 (18.60, 30.86) | 1049.25 (786.92, 1272.86) |  | 89.82 | 0.88 (0.49, 1.27) |

ASDR: age-standardized DAYLs rate; CI: confidence interval; DALYs: disability-adjusted life years; EAPC: estimated annual percentage change; UI: uncertainty interval.
